# Causal relationship between obesity, lifestyle factors and risk of benign prostatic hyperplasia: a univariable and multivariable Mendelian randomization study

**DOI:** 10.1186/s12967-022-03722-y

**Published:** 2022-10-29

**Authors:** Yong-Bo Wang, Lan Yang, Yu-Qing Deng, Si-Yu Yan, Li-Sha Luo, Ping Chen, Xian-Tao Zeng

**Affiliations:** 1 Center for Evidence-Based and Translational Medicine, Zhongnan Hospital of Wuhan University, Wuhan, 430071 China; 2 Division of Medical Affairs, Zhongnan Hospital of Wuhan University, Wuhan, 430071 China; 3 Department of Urology, The Central Hospital of Wuhan, Tongji Medical College, Huazhong University of Science and Technology, Wuhan, 430014 China; 4 Department of Urology, Zhongnan Hospital of Wuhan University, No. 169, Donghu Road, Wuchang District, Wuhan, 430071 China

**Keywords:** Obesity, Lifestyle factors, Benign prostatic hyperplasia (BPH), Mendelian randomization (MR), Causal effect

## Abstract

**Background:**

Obesity (waist circumference, body mass index (BMI)) and lifestyle factors (dietary habits, smoking, alcohol drinking, Sedentary behavior) have been associated with risk of benign prostatic hyperplasia (BPH) in observational studies, but whether these associations are causal is unclear.

**Methods:**

We performed a univariable and multivariable Mendelian randomization study to evaluate these associations. Genetic instruments associated with exposures at the genome-wide significance level (*P* < 5 × 10^–8^) were selected from corresponding genome-wide associations studies (n = 216,590 to 1,232,091 individuals). Summary-level data for BPH were obtained from the UK Biobank (14,126 cases and 169,762 non-cases) and FinnGen consortium (13,118 cases and 72,799 non-cases). Results from UK Biobank and FinnGen consortium were combined using fixed-effect meta-analysis.

**Results:**

The combined odds ratios (ORs) of BPH were 1.24 (95% confidence interval (CI), 1.07–1.43, *P* = 0.0045), 1.08 (95% CI 1.01–1.17, *P* = 0.0175), 0.94 (95% CI 0.67–1.30, *P* = 0.6891), 1.29 (95% CI 0.88–1.89, *P* = 0.1922), 1.23 (95% CI 0.85–1.78, *P* = 0.2623), and 1.04 (95% CI 0.76–1.42, *P* = 0.8165) for one standard deviation (SD) increase in waist circumference, BMI, and relative carbohydrate, fat, protein and sugar intake, 1.05 (95% CI 0.92–1.20, *P* = 0.4581) for one SD increase in prevalence of smoking initiation, 1.10 (95% CI 0.96–1.26, *P* = 0.1725) and 0.84 (95% CI 0.69–1.02, *P* = 0.0741) for one SD increase of log-transformed smoking per day and drinks per week, and 1.31 (95% CI 1.08–1.58, *P* = 0.0051) for one SD increase in sedentary behavior. Genetically predicted waist circumference (OR = 1.26, 95% CI 1.11–1.43, *P* = 0.0004) and sedentary behavior (OR = 1.14, 95% CI 1.05–1.23, *P* = 0.0021) were associated with BPH after the adjustment of BMI.

**Conclusion:**

This study supports independent causal roles of high waist circumference, BMI and sedentary behavior in BPH.

**Supplementary Information:**

The online version contains supplementary material available at 10.1186/s12967-022-03722-y.

## Introduction

Benign prostate hyperplasia (BPH) is a common benign disease in middle-aged and elderly men, appearing in approximately 8% of men by age 40, but up to 90% of men by age 90 [1]. The incident cases of BPH increased by 105.70% from 1990 to 2019 according to the Global Burden of Disease 2019 data [2]. BPH is often underestimated and underdiagnosed. If patients are not treated in time, it may lead to serious complications, such as urinary retention, renal insufficiency and renal failure [3].

Epidemiological studies have reported several potential risk factors for BPH, including obesity [4–6], dietary habits [7–10], smoking [11], alcohol consumption [12–15] and sedentary behavior [16]. However, most of the evidence for the associations have been inconsistent and inconclusive. More importantly, in observational epidemiological studies, reverse causality, misclassification, unobserved confounding, and other biases may largely impede causal inference of these associations. For example, abdominal obesity and overall obesity are strongly related, and their independent association with BPH is unclear. Similarly, smoking and drinking may be overlapping behaviors for individuals, which may introduce residual confounding in traditional observational studies. Identifying the causal association of potentially modifiable risk factors with BPH has important practical implications for exploring the etiology of the disease and for its prevention and management in public health. Mendelian randomization (MR) designs use genetic variation as instrumental variables (IVs) for exposure and enhance causal association [17]. The method can reduce the influence of residual confounding since genetic variants are randomly distributed at conception and therefore unrelated to environmental and self-adopted lifestyle confounders [18]. Furthermore, MR designs can reduce the possibility of reverse causality, since genetic variants could not be altered by disease occurrence and progression.

Therefore, we performed a two-sample univariable and multivariable MR study to evaluate the possible causal associations of abdominal obesity (measured as waist circumference), overall obesity (measured as body mass index (BMI)), lifestyle factors (dietary habits, smoking, alcohol drinking, and sedentary behavior) with risk of BPH.

## Methods

### Study design and genetic instrument selection

**Fig. 1:**
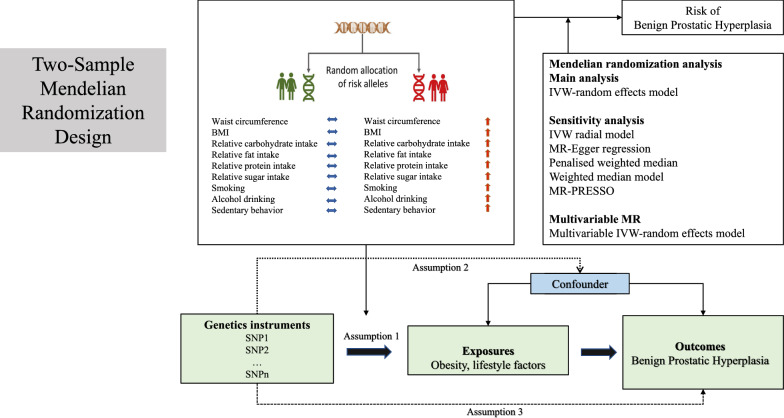
Overview and assumptions of the Mendelian randomization study design. Assumption 1: the instrumental variables should be closely related to the risk factor of interest; assumption 2: the instrumental variables should not be associated with potential confounders, and assumption 3: the instrumental variables should affect the risk of outcome only through risk factors and not through other alternative pathways. LD, Linkage disequilibrium; SNP, single nucleotide polymorphisms; BMI, body mass index; IVW, inverse-variance weighted; PRESSO, Pleiotropy Residual Sum and Outlier

Figure 1 shows the study design and the assumptions of MR in our study [19]. This IV analysis mimics randomized controlled trial with respect to the random allocation of single nucleotide polymorphisms (SNPs) in offspring (independent of confounding factors such as sex and age). We obtained the genetic IVs for the exposures from published genome-wide association studies (GWASs) using summary data [20–24]. We selected SNPs that were associated with waist circumference [20], waist circumference adjusted for BMI [20], BMI [21], dietary habits, smoking behaviors [22, 23], alcohol per week [22], and sedentary behavior [24] at the genome-wide significance threshold (*P* < 5 × 10^–8^). For dietary habits, four sets of instruments (SNPs for relative carbohydrate, fat, protein and sugar intake) were employed for validation [25]. For smoking behaviors, three sets of instruments (SNPs for smoking initiation [22], smoking per day [22], and lifetime smoking index [23]) were employed for validation. For example, the SNPs for smoking initiation were chosen from a meta-analysis of GWAS with a total of 1,232,091 participants of European ancestry. This phenotype was measured as a binary variable, coded as “1” if they had never been a regular smoker in their life and “2” if they had ever been a regular smoker in their life (current or former). The GWAS of number of cigarettes per day came from the Sequencing Consortium of Alcohol and Nicotine use, 23andMe and UK Biobank. The cigarettes per day was defined as the average number of cigarettes smoked per day both in current smoker and former smoker. The GWAS on the lifetime smoking index included information on duration of smoking, heaviness and cessation, which were combined into a simulated half-life (τ) constant and a lifetime smoking index. We obtained summary-level data of alcohol consumption from a GWAS of number of drinks per week in 941,280 individuals. The GWAS was from several cohorts including deCODE, UK Biobank and 23andMe. Drinks per week was defined as the amount of drinks a study participant reported drinking per week, including different types of alcohol. Detailed information on used data sources, definition, unit, participants included in the analysis, adjusted covariates and identified SNPs are displayed in Additional file 1: Table S1.

**Fig. 2:**
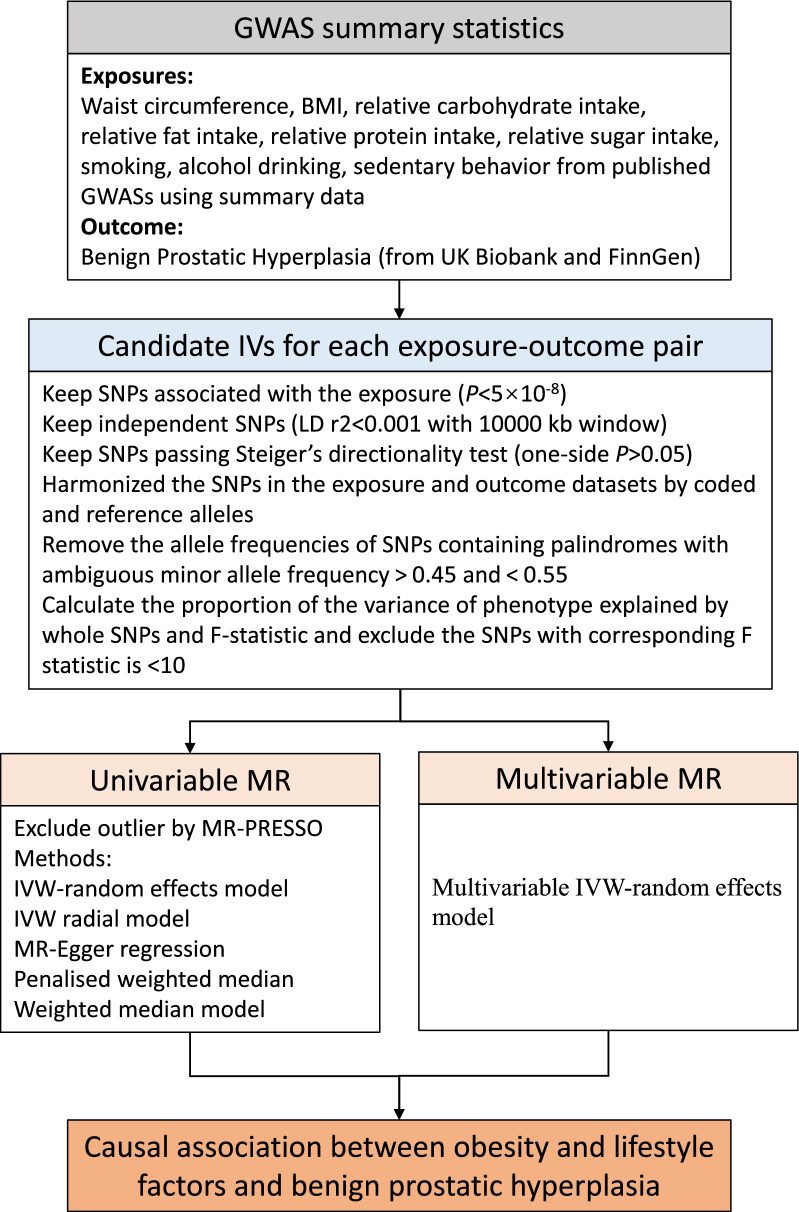
The flow chart of the inclusion and exclusion criterion of candidate SNPs for each exposure-outcome pair. GWAS, genome-wide association studies; BMI, body mass index; LD, Linkage disequilibrium; IVW, inverse-variance weighted; PRESSO, Pleiotropy Residual Sum and Outlier; MR, Mendelian randomization

We calculated the linkage disequilibrium (LD) between SNPs for every risk factor based on LD reference panel from 1000 Genomes of European populations. We excluded SNPs in LD (*r*^2^ > 0.001 and clump window < 10,000 kb) and retained the SNP with the lowest *P* value. Then, we harmonized the SNPs in the exposure and outcome datasets by coded and reference alleles. Exposure and outcome data are unified into a dataset by removing the allele frequencies of SNPs containing palindromes [19]. We defined palindromic SNPs with ambiguous minor allele frequency > 0.45 and < 0.55 [26]. Considering the limited impact of a small proportion of missing generates on the results, a few missing instruments tools in the outcome datasets were not replaced by proxy SNPS. We calculated the proportion of the variance of phenotype explained by whole SNPs and F-statistic [27]. When the corresponding F statistic is > 10, it is considered to be sufficient. We used an online tool to estimate the Power (https://shiny.cnsgenomics.com/mRnd/) [28]. The flow chart of our study is shown in Fig. 2, including the inclusion and exclusion criterion of candidate SNPs for each exposure-outcome pair. Detailed information on genetic instruments are shown in Additional file 1: Table S2.

### Data sources for benign prostatic hyperplasia

Summary-level genetic data of GWASs for BPH were acquired from the R5 release of the FinnGen consortium [29] and the UK Biobank study [30] (Additional file 1: Table S1). In the FinnGen consortium, BPH cases were defined by N40 in International Classification of Diseases-Tenth (ICD-10) Revision code and 600 in ICD-8 and ICD-9. The R5 release of the FinnGen consortium data with a total of 13,118 BPH cases and 72,799 non-cases were obtained. In this data set, individuals with undefined sex, high genotype deletion (> 5%), excess heterozygosity (± 4 standard deviations ((SDs)), and non-Finnish ancestry were excluded. Correlation tests were adjusted for age, 10 genetic principal components and genotyping batches. In the UK Biobank study, the disease was defined by N40 in ICD-10, self-reported operation codes, and office of population and censuses surveys. Data from the UK Biobank study included 183,888 participants (14,126 BPH and 169,762 non-cases) after exclusion of individuals who had withdrawn consent from the UK Biobank study, individuals with sexual chromosome aneuploidies, and individuals of non-European ancestry. Correlation tests had been adjusted for age and up to 20 main components. Detailed information on quality control refers to the web and cited GWAS papers of FinnGen consortium and the UK Biobank [29, 30].

All cited summary-level data from published GWASs and consortia had been approved by the relevant review committees and the participants involved had given informed consent, and the analytic process was in accordance with the STROBE-MR guidelines [31].

### Statistical analysis

We applied MR-Steiger analysis to test the direction of the potential causal association between each of the extracted SNPs on the risk factors and BPH [32]. Heterogeneity between the SNPs was evaluated by calculating Cochrane’s Q statistic. Heterogeneity was considered to exist when Cochrane Q-derived *P* < 0.05, and we used a random-effect inverse-variance weighted (IVW) model as the main analysis method [33]. Five other sensitivity analysis methods, including the weighted median, MR-Egger regression, penalized weighted median, IVW radial regression and MR-PRESSO (Pleiotropy Residual Sum and Outlier) methods, were performed to assess the robustness of the results [33–35]. The weighted median model provides consistent estimates on the condition that ≥ 50% of the weight in the analysis comes from valid IVs [36]. The MR-Egger regression analysis can detect and correct for directional pleiotropy whereas it compromises power. The *P* value for the MR-Egger intercept was used to indicate directional pleiotropy. Under the assumption that no more than 50% of the estimated weight of MR effects is derived from multi-effect SNPs, the penalized weighted median method gives consistent estimates of effects, where the weight is depended on the strength of their association with exposure [36]. The IVW radial regression uses modified second-order weights to test and remove outlying SNPs. The MR-PRESSO method can identify outliers and generate estimates after the outliers are removed [37]. The MR-PRESSO distortion test is designed to test the difference in the estimation before and after the outlier correction. A *P* < 0.05 of the distortion test indicates that there is a significant difference in the estimation before and after the outlier correction. To evaluate whether genetic liability to smoking is associated with BPH risk independently of alcohol drinking, a multivariable random-effects IVW model was employed to adjust for genetically predicted alcohol drinking in the analysis of smoking and vice versa [38, 39]. In addition, to assess whether genetic liability to lifestyle factors are associated with BPH risk independently of BMI, we performed multivariable MR analyses by random-effects IVW model with adjustment for genetically predicted BMI. Derived estimates based on the UK Biobank and the FinnGen consortium were summarized using fixed-effects meta-analyses. Odds ratios (ORs) and 95% confidence intervals (CIs) for the risk of BPH were scaled to per one standard deviation (SD) increase in waist circumference, BMI, relative carbohydrate, fat, protein, and sugar intake, lifetime smoking index and sedentary behavior, one SD increase in prevalence of smoking initiation, and one SD increase of log-transformed alcoholic smoking per day and drinks per week. To account for multiple testing in our analyses, a Bonferroni-corrected threshold of *P* < 0.0046 (α = 0.05/11 exposure factors) was applied. Associations with *P* < 0.0046 were considered significant, and associations with *P* ≥ 0.0046 and < 0.05 were considered suggestive. All statistical analyses were conducted using the Mendelian Randomization (0.4.2), TwoSampleMR (0.5.5), MRPRESSO (1.0), MVMR (0.3) and meta (4.11.0) packages in R, version 4.0.3.

## Results

The F statistics for IVs and estimated power for all analyses are shown in Additional file 1: Table S3. None of these IVs had an F-statistic below the threshold of 10, indicating that there was low evidence of weak instrument bias in this study. Expected ORs for BPH was estimated for Given a α = 5% and 80% power in the FinnGen consortium and UK biobank study. The power was low in the analysis of alcohol per week, but adequate for the other studied exposures.

The Steiger-MR indicated that the SNPs explained more variance in exposure than the outcome (all *P* > 0.05), which identified the robustness of the causal effect estimates. No pleiotropy was identified in the analysis of all exposures in the UK Biobank and FinnGen data by the MR-Egger regression (Additional file 1: Table S4). Moderate heterogeneity was found in the analysis of most exposures (*P* for Cochrane’s Q < 0.001) (Additional file 1: Table S4).

**Fig. 3:**
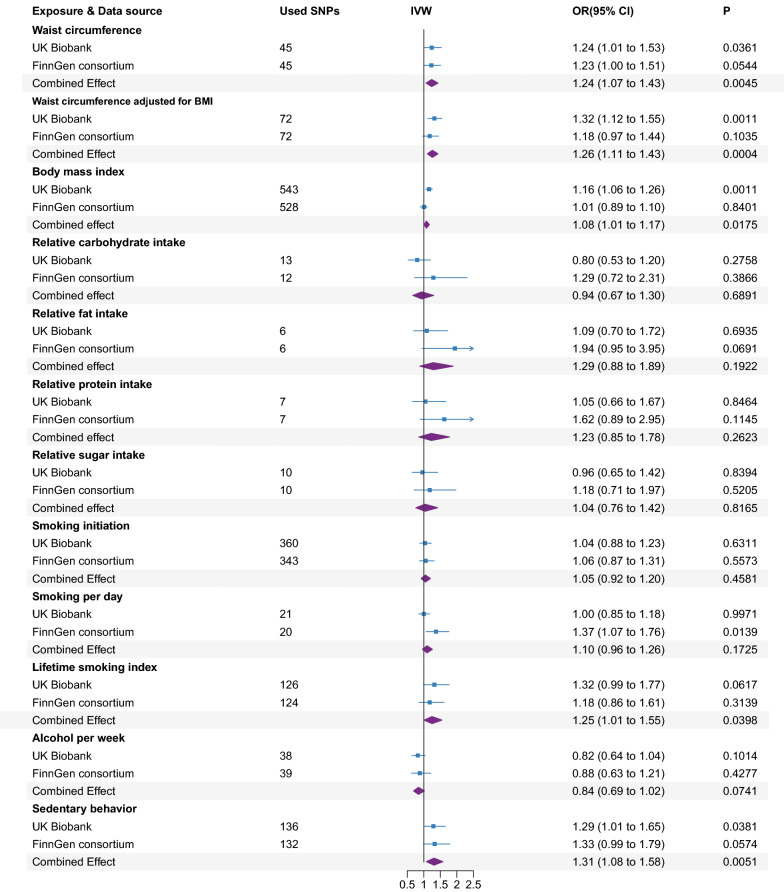
Associations of genetically predicted risk factors with benign prostatic hyperplasia using random effect inverse-variance weighted method. IVW, inverse-variance weighted; OR, odds ratio; CI confidence interval; BMI, body mass index; SNP, single nucleotide polymorphism

Genetically predicted higher waist circumference, waist circumference adjusted for BMI and BMI were associated with a raised risk of BPH in UK Biobank data (Fig. 3). The estimates were retained in FinnGen consortium data, but with wider CIs that span 1.0. The combined ORs of BPH were 1.24 (95% CI 1.07–1.43, *P* = 0.0045), 1.26 (95% CI 1.11–1.43, *P* = 0.0004) and 1.08 (95% CI 1.01–1.17, *P* = 0.0175) for waist circumference, waist circumference adjusted for BMI and BMI, respectively. The association calculated by different methods (the weighted median, MR-Egger regression, penalized weighted median, and IVW radial regression) was directionally consistent (Additional file 1: Figure S1). After removing outliers in the MR-PRESSO analysis, the association between waist circumference, waist circumference adjusted for BMI and BMI and BPH persisted and the *P* value for the distortion test were above 0.05 (Additional file 1: Table S4).

**Fig. 4:**
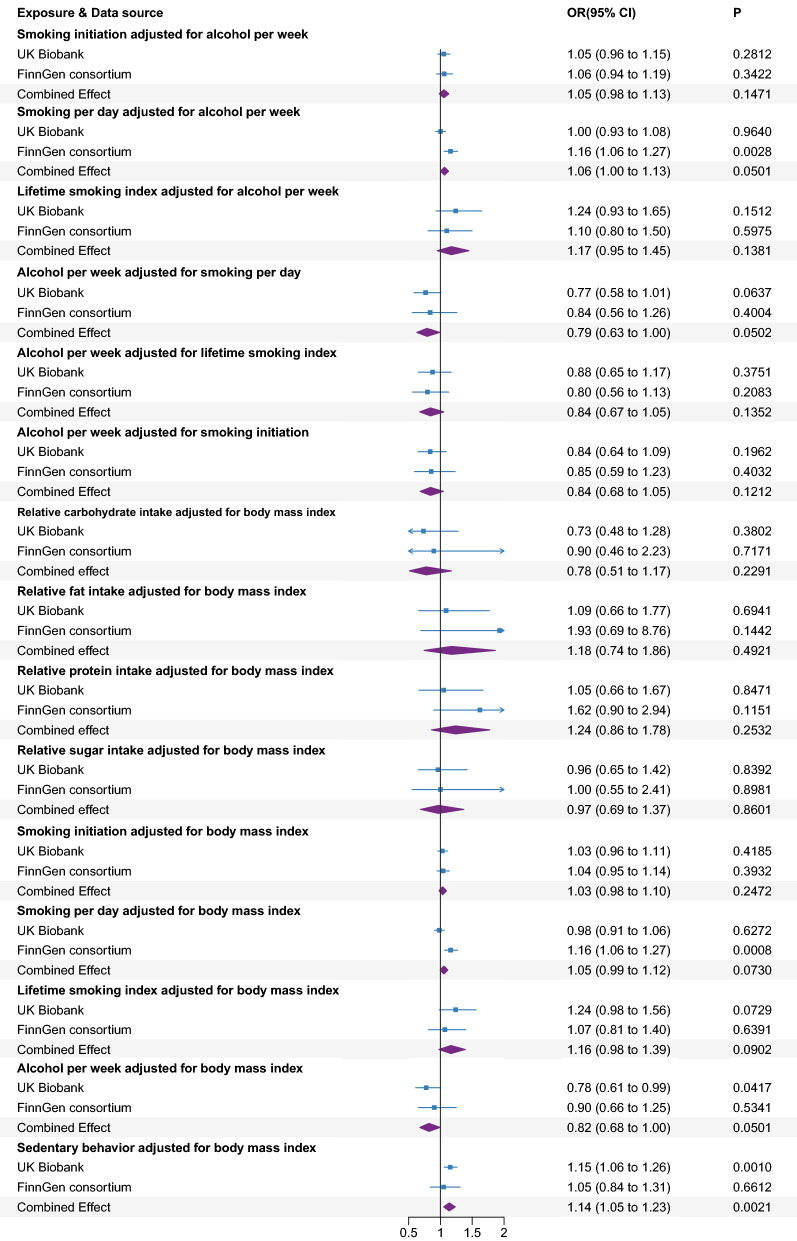
Associations of genetically predicted risk factors with benign prostatic hyperplasia using multivariable MR analyses. OR, odds ratio; CI, confidence interval

As for lifestyle factors, in terms of dietary habits, the combined ORs of BPH were 0.94 (95% CI 0.67–1.30, *P* = 0.6891), 1.29 (95% CI 0.88–1.89, *P* = 0.1922), 1.23 (95% CI 0.85–1.78, *P* = 0.2623), and 1.04 (95% CI 0.76–1.42, *P* = 0.8165) for one SD increase in relative carbohydrate, fat, protein, and sugar intake, respectively, in the meta-analysis of data from the UK Biobank and FinnGen consortium (Fig. 3). These null associations were replicated in the sensitivity analysis and multivariable MR analysis (Additional file 1: Figure S1 and Fig. 4).

As for smoking behaviors, genetic predisposition to smoking initiation (combined OR 1.05, 95% CI 0.92–1.20, *P* = 0.4581) and smoking per day (combined OR 1.10, 95% CI 0.96–1.26, *P* = 0.1725) were not associated with BPH in the meta-analysis of data from the UK Biobank and FinnGen consortium (Fig. 3). This null association was replicated in the sensitivity analysis (Additional file 1: Figure S1). In the multivariable MR analysis adjusting for alcohol per week and BMI separately, we also detected no association between genetic susceptibility to smoking initiation and smoking per day and risk of BPH (Fig. 4). A suggestive positive association between genetically predicted lifetime smoking index and BPH was detected in the meta-analysis (combined OR 1.25, 95% CI 1.01–1.55, *P* = 0.0398) (Fig. 4). However, the association did not remain after adjusting for alcohol per week (combined OR 1.17, 95% CI 0.95–1.45, *P* = 0.1380) and BMI (combined OR 1.16, 95% CI 0.98–1.39, *P* = 0.0902), respectively (Fig. 4). In addition, genetically predicted alcohol per week was not associated with BPH neither in a univariable model (combined OR 0.84, 95% CI 0.69–1.02, *P* = 0.0741) or in a multivariable MR analysis adjusting for smoking behaviors and BMI, respectively (Fig. 3, 4, Additional file 1: Figure S1).

However, genetically predicted higher sedentary behavior was associated with an elevated risk of BPH in UK Biobank data (Fig. 3). The estimate was unchanged in FinnGen consortium data, but with a broader CI. The combined ORs of BPH was 1.31 (95% CI 1.08–1.58, *P* = 0.0051) for sedentary behavior. The association was directionally consistent in the sensitivity analyses (Additional file 1: Figure S1). After the adjustment of BMI, a significantly positive association was also found between genetic predisposition to sedentary behavior and risk of BPH (OR = 1.14, 95% CI 1.05–1.23, *P* = 0.0021) (Fig. 4).

## Discussion

Our MR study supports that genetic predisposition to higher waist circumference and sedentary behavior are independently and causally associated with the risk of BPH. Suggestive causal association is observed between genetic predisposition to higher BMI and increased risk of BPH. There is no obvious evidence that genetic predisposition to relative carbohydrate, fat, protein, and sugar intake, smoking and alcohol drinking are causally associated with the risk of BPH.

It is reported that obesity, especially visceral obesity, has been associated with BPH in observational studies [40, 41]. A meta-analysis of 7 cohort studies and 12 case–control studies reported a positive association between BPH and BMI [4]. In the subgroup analysis of population-based case–control studies, BMI was associated with a dose–response relationship with BPH, and a marginal positive association was detected between BPH risk with higher BMI. However, some prospective studies have not confirmed the association between BMI and BPH [5, 6]. This difference may be due to the different covariate adjustments and small sample sizes in these studies. In this study, the association between waist circumference and BPH remained after the adjustment for BMI, which suggested that central obesity is a vital risk factor as overall fat mass for BMI. There is a possible mechanism that could explain why obesity itself results in BPH even in metabolically healthy populations. Previous studies have observed that obesity can lead to chronic systemic inflammation and oxidative stress, leading to prostate tissue immune cell infiltration, tissue remodeling and hyperplasia in prostate tissues [40, 42, 43].

A systematic review evaluated myriads of food types and food groups and found consumption of a high-calorie diet, high in starches and red meat may be weakly associated with BPH risk, while a lower-calorie diet, high in polyunsaturated fats and low in saturated fat may be weakly associated with decreased risk [7–10]. The Prostate Cancer Prevention Trial (PCPT) within a cohort of 18,800 patients aged more than 50 years found a diet low in fat and red meat and high in protein may reduce the risk of symptomatic BPH [7]. However, with regard to the role of a high-fat diet, the evidence from the literature is not consistent. Suzuki et al. found that BPH risk was not associated with energy-adjusted total fat intake [8]. The above-mentioned findings led us to emphasize that the exact association between different dietary patterns and BPH has not yet been fully elucidated. Our study did not detect associations between relative carbohydrate, fat, protein, and sugar intake and BPH in the univariable and multivariable analyses after adjusting for BMI, although we could not rule out the possibility that the observed null findings were led by an inadequate power.

Several previous observational studies have reported that smoking confers an aggravated effect on BPH [44, 45], while other studies have found a protective effect [46]. A meta-analysis of 1 case–control study, 6 cohort studies, and 1 cross-sectional study representing data from 44,100 subjects, found no association between smoking and BPH risk, either for current smokers or for ex-smokers [11]. In our study, we did not find statistically significant associations with BPH for genetic predisposition to smoking behaviors in the univariable analyses and multivariable analyses after adjusting for alcohol behavior and BMI, although positive associations were showed in all analyses. More well-powered MR analysis is needed to verify our finding. Nevertheless, several vivo and vitro studies have shown that nicotine may increase activity in the sympathetic nervous system and may cause symptoms of urine storage by increasing the tone of the bladder smooth muscles [45]. Several studies have suggested that smoking may reduce the levels of testosterone, and this effect could increase the risk of BPH [47–49]. In addition, smoking could cause nutritional imbalance, which may affect bladder and collagen synthesis [50]. It may also affect bladder wall strength and detrusor instability [51].

There are conflicting data on the association between BPH and alcohol consumption in observational studies [12–15]. Several studies have found that mild alcohol intake could increase lower urinary tract symptoms (LUTS) through diuretic effects or increased sympathetic nervous system activity, while moderate and high alcohol consumption may reduce the risk of BPH and complications of severe LUTS by altering androgen levels [52]. A meta-analysis including sixteen studies were conducted and the authors divided total alcohol intake per day into six gm levels [12]. At all six levels, alcohol intake was associated with slightly or significantly reduced likelihood of BPH. However, this finding was majorly based on cross-sectional and case-control studies and therefore were prone to residual confounding (e.g., other alcohol-related behaviors and lifestyles) and misclassification bias. Our study did not detect a negative association between alcohol consumption and BPH in the multivariable analyses after adjusting for smoking behavior and BMI, although our null findings are also at risk by inadequate power.

Several evidences support the role of sedentary behavior in endangering LUTS/BPH [53, 54]. In one large prospective study, men with the highest levels of physical activity were 19% less likely to develop moderate or worse LUTS than men with the lowest level [55]. Men who watched more than 30 h of television (TV) per week were more likely to develop moderate or more severe LUTS than men who watched less than 1 h of TV per week [55]. A cross-sectional study found that reducing sedentary time had a protective effect and reduced the prevalence of BPH [56]. However, their findings were prone to residual confounding from other sedentary related behaviors and lifestyles as well as misclassification bias. In our study, the proportion of time spent sitting throughout life appears to significantly increase the risk of BPH. Our MR study strengthened the causal nature of the positive association between sedentary behavior and BPH and further revealed that this association was independent of BMI. This result has potential implications for the prevention of BPH in ageing societies, where the incidence of BPH is gradually increasing.

MR analysis has three important assumptions [17, 57]. First, the selected IVs should be strongly associated with the exposure. In the present study, we selected SNPs that were associated with the exposures at the genome-wide significance level (*P* < 5 × 10^–8^) as IVs from GWASs with large sample sizes. Second, IVs should be independent of potential confounders. Given the study was based on summary-level data, a thorough examination of the associations between exposures and possible confounders was not possible. However, these IVs were widely used in previous MR studies [58, 59]. Third, the genetic IVs should affect the outcome only via the exposure, not via other alternative pathways. Although we could not completely rule out the possibility that our findings might be biased by horizontal pleiotropy, our results remained consistent across several sensitivity analyses and the MR-Egger and MR-PRESSO analyses detected limited evidence in support of strong pleiotropic effects. This is the first MR study to investigate the casual association between obesity, lifestyle factors and risk of BPH using two-sample MR analyses. The main advantages of this study were the MR design and the large number of cases of BPH. Similar results in two independent populations support the reliability and robustness of our results. In addition, the results remained overall consistent across several sensitivity analyses. The MR directionality test (Steiger method) supported evidence that our genetic IVs influences our exposure before our outcome as opposed to the opposite direction of effect. Moreover, all of the participants recruited in the GWASs were from European descent. Therefore, our findings were unlikely distorted by demographic stratification biases.

Limitations need to be considered in this study. First, a potential concern in any MR study is horizontal pleiotropy. In our study, we did not observe obvious pleiotropic effects in all the analyses of exposures from MR-Egger intercept test. In addition, there were few outliers detected by MR-PRESSO analysis and the associations remained consistent after the removal of outlying SNPs. For smoking and alcohol behavior, we observed no association between genetically predicted smoking behavior and BPH after adjusting for alcohol consumption in the multivariable MR analysis and vice versa. Given the consistent associations of genetic predisposition to high waist circumference, BMI and sedentary behavior with BPH across two data sources and different MR models, it is unlikely that these results are driven by horizontal pleiotropy. Second, overfitting may be a concern, as both smoking and alcohol GWAS contain a certain percentage of data from the UK Biobank. However, sample overlap was unlikely to mislead our results, as our IVs were chosen from large GWASs. Our SNPs were chosen at a strong genome-wide threshold (*P* < 5 × 10^–8^) with all estimated F statistics exceeding 10, suggesting that the bias introduced by partial sample overlap should be minimal. Meanwhile, we interpreted the associations based on the results of meta-analyses by FinnGen and UK Biobank, with nearly half of the weights were derived from FinnGen. Third, even though our analyses were based on FinnGen and UK Biobank with large sample sizes, we may overlook weak associations, especially exposures consisting of a few SNPS that explain small phenotypic variations. Fourth, for lifestyle factors, whether the observed associations differ by age, metabolic level and other potential factors and BPH severity could not be examined based on summary-level data in this study. Fifth, the population restriction in our study may limit the generality of our findings in other populations. Sixth, for alcohol consumption, the effects of different types of alcohol are indistinguishable, and the nonlinear association could not be evaluated in our present MR analysis. Similarly, the gene-environmental interaction could not be estimated in summary-level genetic statistics. Finally, further validation studies should be performed to explore the association between other risk factors and BPH [60].

BPH is a major prostate disease in men that increase with aging. Previous traditional epidemiological studies with small sample size had explained some common related factors and did not perform causal inference. Therefore, risks of BPH were not fully understood. To validate the causality, we performed a two-sample Mendelian randomization analyses. In conclusion, our study provides MR evidence supporting a significantly causal role of waist circumference and sedentary behavior in BPH. The suggestive association between genetic predisposition to higher BMI and BPH risk needs verification. Dietary habits, smoking and alcohol drinking need confirmation in well-powered studies. Although many of these risks have not been fully studied, they might be beneficial in providing information to assist in counselling of patients and help to form strategies for the prevention of BPH. Meanwhile, in the future studies, identifying comprehensive risk factors on BPH, and developing freely accessible prediction models for the BPH can identify individuals at particular risk and provide decision-making supports for individualized intervention.

## Supplementary Information

**Additional file1: Table S1.** Detailed information on used studies. **Table S2.** Detailed information on genetic instruments. **Table S3.** Variance explained, average F-statistic, and power calculation. **Table S4.** The results of pleiotropy test, Cochrane’s Q and MR-PRESSO. **Figure S1.** Associations of genetically predicted risk factors with benign prostatic hyperplasia using multiple MR sensitivity analyses. IVW, inverse-variance weighted; OR, odds ratio; CI, confidence interval; BMI, body mass index; SNP, single nucleotide polymorphism; MR, Mendelian randomization.

## Data Availability

The datasets analyzed in this study are publicly available summary statistics. Data used can be obtained upon a reasonable request to the corresponding author.

## Abbreviations

BMI: Body mass index
BPH: Benign prostate hyperplasia
CI: Confidence interval
ICD-10: International Classification of Diseases-Tenth
IV: Instrumental variable
IVW: Inverse-variance weighted
GWASs: Genome-wide association studies
LD: Linkage disequilibrium
LUTS: Lower urinary tract symptoms
MR: Mendelian randomization
OR: Odds ratio
PRESSO: Pleiotropy Residual Sum and Outlier
SD: Standard deviation
SNP: Single nucleotide polymorphism
