# Supplementary material for: Causal relationship between obesity, lifestyle factors and risk of benign prostatic hyperplasia: a univariable and multivariable Mendelian randomization study
Source: J Transl Med. 2022 Oct 29;20:495. doi: 10.1186/s12967-022-03722-y (PMC9617448; doi:10.1186/s12967-022-03722-y)
Supplement: Supplementary file 1 — Additional file1: Table S1. Detailed information on used studies. Table S2. Detailed information on genetic instruments. Table S3. Variance explained, average F-statistic, and power calculation. Table S4. The results of pleiotropy test, Cochrane’s Q and MR-PRESSO. Figure S1. Associations of genetically predicted risk factors with benign prostatic hyperplasia using multiple MR sensitivity analyses. IVW, inverse-variance weighted; OR, odds ratio; CI, confidence interval; BMI, body mass index; SNP, single nucleotide polymorphism; MR, Mendelian randomization. [file 12967_2022_3722_MOESM1_ESM.docx]

**Causal relationship between obesity, lifestyle factors and risk of benign prostatic hyperplasia: A univariable and multivariable Mendelian randomization study**

**Supplementary Files**

Supplementary Table 1. Detailed information on used studies

Supplementary Table 2. Detailed information on genetic instruments

Supplementary Table 3. Variance explained, average F-statistic, and power calculation

Supplementary Table 4. The results of pleiotropy test, Cochrane’s Q and MR-PRESSO

Supplementary Figure 1. Associations of genetically predicted risk factors with benign prostatic hyperplasia using multiple MR sensitivity analyses.

IVW, inverse-variance weighted; OR, odds ratio; CI, confidence interval; BMI, body mass index; SNP, single nucleotide polymorphism; MR, Mendelian randomization.

Supplementary Table 1. Detailed information on used studies

| Exposure or outcome | Definition | Unit | Participants included in analysis | Adjustments | Identified SNPs | PubMed ID/Web source |
| --- | --- | --- | --- | --- | --- | --- |
| Obesity |  |  |  |  |  |  |
| Waist circumference | - | SD | 224,459 European-descent individuals | Age and study-specific covariates | 45 | 25673412 |
| Waist circumference adjusted for BMI | - | SD | 224,459 European-descent individuals | Age, body mass index and study-specific covariates | 72 | 25673412 |
| Body mass index | Body mass divided by the square of the body height | SD (~4.8kg/m^2^) | 806,834 European-descent individuals | Age, sex, and genetic 1-5 principal components | 543 | 25673413 |
| Lifestyle factors |  |  |  |  |  |  |
| Relative carbohydrate intake* | Carbohydrate included intake from all saccharides, while sugar included  intake from mono- and disaccharides only. The four dietary composition phenotypes were measured as relative intake, which could be described as an “adjusted macronutrients density”. | SD | 268,922 European-descent individuals | Age, sex, and up to 20 genetic principal components | 13 | 32393786 |
| Relative fat intake* |  | SD | 268,922 European-descent individuals | Age, sex, and up to 20 genetic principal components | 6 | 32393786 |
| Relative protein intake* |  | SD | 268,922 European-descent individuals | Age, sex, and up to 20 genetic principal components | 7 | 32393786 |
| Relative sugar intake* |  | SD | 235,391 European-descent individuals | Age, sex, and up to 20 genetic principal components | 10 | 32393786 |
| Smoking initiation | Probability of ever smoked regularly | SD in prevalence of smoking initiation | 1232,091 European-descent individuals | Age, sex, and the first ten genetic principal components | 360 | 30643251 |
| Smoking per day | Average number of cigarettes smoked per day both in current smoker and former smoker | SD increase of log-transformed alcoholic smoking/ day | 216,590 European-descent individuals | Age, sex, and the first 10 genetic principal components | 21 | 30643251 |
| Lifetime smoking index | Takes into account smoking status as well as smoking duration, heaviness, and cessation in ever smokers | SD increase of lifetime smoking index | 462,690 European-descent individuals | Genotyping chip and sex | 126 | 31689377 |
| Alcohol per week | Amount of drinks a study participant reported drinking per week | SD increase of log-transformed alcoholic drinks/ week | 941,280 European-descent individuals | Age, sex, and the first ten genetic principal components | 38 | 30643251 |
| Sedentary behavior | Hours of sedentary behavior per day | SD in hours/day | 437,887 European-descent individuals | Age, sex, and genotyping chips | 136 | MRC-IEU: ukb-b-5192 |
| Outcome |  |  |  |  |  |  |
| Benign prostatic hyperplasia | - | - | 14,126 Benign prostatic hyperplasia cases and 169,762 non-cases | Age, sex, and up to 20 genetic principal components | - | UK Biobank (http://www.nealelab.is/uk-biobank) |
| Benign prostatic hyperplasia | - | - | 13,118 Benign prostatic hyperplasia cases and 72,799 non-cases | Age, sex, 10 genetic principal components, and genotyping batch | - | FinnGen consortium (https://www.finngen.fi/fi) |

OR, Odds ratio; SD, Standard deviation; SNPs, single nucleotide polymorphism.

*Specifically, phenotype definition of the energy-corrected macronutrient intakes is given by

corrected intake=$\frac{energy from macronutrient}{{total energy}^{\beta}}$

Here, the macronutrient and total energy intake was measured in the same unit (e.g., kilocalories). β is a correction factor and detailed estimated procedure was described by Meddens et al.

Supplementary Table 2. Detailed information on genetic instruments

| Exposure | SNP | Chr.Position | EA | OEA | EAF | BETA | SE | P-value |
| --- | --- | --- | --- | --- | --- | --- | --- | --- |
| Body mass index | rs17024393 | 1:110154688 | T | C | 0.9689 | -0.0644 | 0.0049 | 7.11E-39 |
| Body mass index | rs197374 | 1:112289983 | T | C | 0.4027 | 0.0141 | 0.0017 | 3.16E-16 |
| Body mass index | rs10779751 | 1:11284336 | A | G | 0.2787 | 0.0131 | 0.0018 | 2.66E-13 |
| Body mass index | rs17544384 | 1:115295160 | T | C | 0.7952 | -0.0131 | 0.0023 | 2.28E-08 |
| Body mass index | rs7534091 | 1:118864616 | A | G | 0.7182 | -0.012 | 0.0018 | 6.65E-11 |
| Body mass index | rs74887628 | 1:147032779 | A | G | 0.0339 | 0.0305 | 0.0054 | 1.76E-08 |
| Body mass index | rs4970991 | 1:151004003 | T | C | 0.2151 | 0.0116 | 0.0021 | 1.81E-08 |
| Body mass index | rs61813324 | 1:156049877 | T | C | 0.1312 | 0.0289 | 0.0028 | 3.20E-24 |
| Body mass index | rs1750307 | 1:156488420 | A | T | 0.3655 | 0.0129 | 0.0018 | 2.78E-13 |
| Body mass index | rs79113395 | 1:1590521 | A | G | 0.266 | -0.02 | 0.0022 | 2.03E-20 |
| Body mass index | rs10733051 | 1:167280354 | A | G | 0.512 | 0.0093 | 0.0016 | 6.96E-09 |
| Body mass index | rs61828641 | 1:174321997 | A | G | 0.1092 | 0.0223 | 0.003 | 2.49E-13 |
| Body mass index | rs543874 | 1:177889480 | A | G | 0.7786 | -0.0479 | 0.002 | 3.06E-125 |
| Body mass index | rs10920678 | 1:190239907 | A | G | 0.425 | 0.0149 | 0.0016 | 7.15E-20 |
| Body mass index | rs2400414 | 1:194965200 | T | C | 0.3461 | -0.0126 | 0.0018 | 5.52E-13 |
| Body mass index | rs61740466 | 1:19934900 | A | G | 0.2346 | -0.0142 | 0.0022 | 2.24E-10 |
| Body mass index | rs2820295 | 1:201800868 | A | G | 0.3266 | 0.0235 | 0.0018 | 5.56E-39 |
| Body mass index | rs10920336 | 1:202115945 | A | G | 0.5327 | -0.0101 | 0.0017 | 4.20E-09 |
| Body mass index | rs1006317 | 1:209552636 | T | G | 0.1268 | 0.016 | 0.0026 | 3.75E-10 |
| Body mass index | rs6661316 | 1:210095527 | T | C | 0.5893 | 0.012 | 0.0016 | 1.72E-13 |
| Body mass index | rs10864728 | 1:230304914 | A | G | 0.3978 | 0.011 | 0.0019 | 1.20E-08 |
| Body mass index | rs4655141 | 1:23312025 | T | C | 0.8362 | -0.0173 | 0.0022 | 6.20E-15 |
| Body mass index | rs946824 | 1:243684019 | T | C | 0.1355 | 0.0197 | 0.0025 | 4.82E-15 |
| Body mass index | rs10909880 | 1:2727804 | T | C | 0.4522 | -0.0135 | 0.0016 | 2.21E-16 |
| Body mass index | rs2228552 | 1:32165495 | T | G | 0.5663 | 0.0124 | 0.0019 | 1.80E-11 |
| Body mass index | rs4653017 | 1:33776728 | T | C | 0.6679 | 0.0118 | 0.0018 | 1.11E-10 |
| Body mass index | rs7512146 | 1:34283008 | T | G | 0.5272 | -0.0097 | 0.0017 | 1.29E-08 |
| Body mass index | rs11577094 | 1:38026600 | T | C | 0.0791 | 0.0186 | 0.003 | 3.28E-10 |
| Body mass index | rs112646560 | 1:39560250 | T | C | 0.2119 | 0.018 | 0.0023 | 1.29E-14 |
| Body mass index | rs2984618 | 1:47690438 | T | G | 0.4408 | 0.0165 | 0.0016 | 3.80E-24 |
| Body mass index | rs657452 | 1:49589847 | A | G | 0.4042 | 0.0188 | 0.0016 | 3.17E-30 |
| Body mass index | rs587271 | 1:54743111 | T | C | 0.69 | 0.012 | 0.0018 | 6.21E-11 |
| Body mass index | rs79906980 | 1:57887985 | T | C | 0.1555 | 0.0158 | 0.0026 | 1.69E-09 |
| Body mass index | rs12140153 | 1:62579891 | T | G | 0.0911 | -0.0353 | 0.0034 | 1.44E-25 |
| Body mass index | rs2503185 | 1:66461401 | A | G | 0.5097 | 0.013 | 0.0017 | 1.33E-14 |
| Body mass index | rs3101336 | 1:72751185 | T | C | 0.3825 | -0.0254 | 0.0016 | 4.80E-54 |
| Body mass index | rs6656785 | 1:75005776 | A | G | 0.6125 | -0.0178 | 0.0016 | 3.06E-27 |
| Body mass index | rs34517439 | 1:78450517 | A | C | 0.1168 | 0.0391 | 0.003 | 3.40E-39 |
| Body mass index | rs6696828 | 1:80812020 | C | G | 0.2976 | 0.012 | 0.0018 | 6.42E-11 |
| Body mass index | rs11121210 | 1:8708529 | T | C | 0.3445 | -0.0111 | 0.0017 | 2.37E-10 |
| Body mass index | rs321237 | 1:96478125 | A | G | 0.7498 | 0.0126 | 0.002 | 1.49E-10 |
| Body mass index | rs11165643 | 1:96924097 | T | C | 0.5796 | 0.0185 | 0.0016 | 4.49E-30 |
| Body mass index | rs12072739 | 1:98315893 | A | G | 0.7762 | -0.0169 | 0.0023 | 1.50E-13 |
| Body mass index | rs17094222 | 10:102395440 | T | C | 0.7912 | -0.0173 | 0.002 | 4.04E-18 |
| Body mass index | rs7907470 | 10:10268989 | A | G | 0.9161 | -0.0177 | 0.0031 | 7.36E-09 |
| Body mass index | rs11594179 | 10:104392580 | T | C | 0.2328 | -0.0109 | 0.0019 | 2.16E-08 |
| Body mass index | rs79780963 | 10:104952499 | T | C | 0.0804 | 0.0244 | 0.0035 | 2.63E-12 |
| Body mass index | rs7903146 | 10:114758349 | T | C | 0.2787 | -0.0178 | 0.0018 | 1.67E-23 |
| Body mass index | rs2257791 | 10:118643670 | A | G | 0.756 | -0.0139 | 0.002 | 1.63E-12 |
| Body mass index | rs845084 | 10:125220036 | A | G | 0.2666 | 0.0136 | 0.0019 | 3.22E-12 |
| Body mass index | rs17636031 | 10:126594078 | T | C | 0.7244 | -0.0154 | 0.0018 | 3.87E-17 |
| Body mass index | rs4880341 | 10:133992689 | T | C | 0.5725 | -0.013 | 0.0017 | 3.06E-14 |
| Body mass index | rs7893571 | 10:16750129 | T | G | 0.6736 | 0.0125 | 0.0018 | 5.83E-12 |
| Body mass index | rs76638898 | 10:21099584 | A | G | 0.0234 | -0.0376 | 0.0065 | 8.15E-09 |
| Body mass index | rs7084454 | 10:21821274 | A | G | 0.3086 | 0.0198 | 0.0018 | 4.51E-27 |
| Body mass index | rs10829164 | 10:27318370 | T | C | 0.1468 | 0.0151 | 0.0024 | 2.37E-10 |
| Body mass index | rs4097319 | 10:33860515 | T | G | 0.5641 | 0.0107 | 0.0017 | 4.32E-10 |
| Body mass index | rs12765914 | 10:34013507 | T | C | 0.0801 | 0.0226 | 0.0031 | 1.96E-13 |
| Body mass index | rs1624134 | 10:34834482 | C | G | 0.3925 | 0.01 | 0.0017 | 6.51E-09 |
| Body mass index | rs12259464 | 10:53680099 | A | G | 0.4811 | 0.0109 | 0.0017 | 1.48E-10 |
| Body mass index | rs7070670 | 10:61842645 | T | C | 0.3228 | -0.0126 | 0.0021 | 7.14E-10 |
| Body mass index | rs10761785 | 10:65318766 | T | G | 0.5136 | -0.0133 | 0.0016 | 3.47E-16 |
| Body mass index | rs12098284 | 10:76047464 | T | C | 0.1212 | 0.0184 | 0.0026 | 9.87E-13 |
| Body mass index | rs11001259 | 10:76807650 | A | T | 0.1767 | -0.0138 | 0.0024 | 1.22E-08 |
| Body mass index | rs7899106 | 10:87410904 | A | G | 0.9496 | -0.0327 | 0.0037 | 1.72E-18 |
| Body mass index | rs10788494 | 10:88110925 | C | G | 0.4835 | 0.0132 | 0.0017 | 5.51E-15 |
| Body mass index | rs147568678 | 10:93061851 | T | C | 0.7684 | 0.0134 | 0.0023 | 3.42E-09 |
| Body mass index | rs2439823 | 10:99778226 | A | G | 0.4514 | -0.0165 | 0.0017 | 6.51E-22 |
| Body mass index | rs2513999 | 11:103019633 | A | G | 0.1593 | -0.0154 | 0.0026 | 3.07E-09 |
| Body mass index | rs12286929 | 11:115022404 | A | G | 0.5007 | -0.0177 | 0.0016 | 1.93E-27 |
| Body mass index | rs76942203 | 11:116973247 | A | G | 0.0587 | 0.0263 | 0.0041 | 9.08E-11 |
| Body mass index | rs3825061 | 11:118944675 | T | C | 0.3863 | 0.014 | 0.0017 | 6.15E-16 |
| Body mass index | rs11218510 | 11:121922587 | A | G | 0.3949 | -0.014 | 0.002 | 6.79E-13 |
| Body mass index | rs35483388 | 11:122545146 | T | C | 0.3794 | 0.0125 | 0.002 | 2.61E-10 |
| Body mass index | rs7944782 | 11:130795698 | T | G | 0.5016 | -0.0144 | 0.0017 | 3.61E-17 |
| Body mass index | rs2007518 | 11:132639606 | A | G | 0.5618 | -0.013 | 0.0017 | 1.96E-14 |
| Body mass index | rs900144 | 11:13294268 | T | C | 0.5784 | 0.0148 | 0.0017 | 1.53E-18 |
| Body mass index | rs1941213 | 11:133125329 | A | C | 0.7114 | 0.0108 | 0.0019 | 1.59E-08 |
| Body mass index | rs329651 | 11:133767622 | T | G | 0.806 | 0.016 | 0.0021 | 2.13E-14 |
| Body mass index | rs12364470 | 11:134601012 | T | G | 0.8535 | -0.0187 | 0.0022 | 2.18E-17 |
| Body mass index | rs2074314 | 11:17411821 | T | C | 0.6356 | 0.0105 | 0.0017 | 1.37E-09 |
| Body mass index | rs6265 | 11:27679916 | T | C | 0.1836 | -0.0413 | 0.0021 | 7.40E-89 |
| Body mass index | rs570463 | 11:28739318 | A | C | 0.3266 | -0.0121 | 0.0018 | 4.33E-11 |
| Body mass index | rs11030618 | 11:29243293 | T | C | 0.5786 | 0.011 | 0.0017 | 1.67E-10 |
| Body mass index | rs2065418 | 11:30422068 | T | G | 0.646 | 0.0139 | 0.0018 | 6.07E-15 |
| Body mass index | rs11246136 | 11:371265 | A | C | 0.1021 | -0.0167 | 0.003 | 2.03E-08 |
| Body mass index | rs2862996 | 11:43653833 | T | G | 0.7012 | -0.0216 | 0.0017 | 3.60E-35 |
| Body mass index | rs10742752 | 11:45438374 | T | C | 0.3761 | -0.0123 | 0.0017 | 1.20E-13 |
| Body mass index | rs118081010 | 11:46174948 | T | C | 0.0165 | 0.0518 | 0.0075 | 5.28E-12 |
| Body mass index | rs7124681 | 11:47529947 | A | C | 0.4162 | 0.0257 | 0.0016 | 3.96E-55 |
| Body mass index | rs6591407 | 11:56914157 | A | C | 0.1984 | -0.0124 | 0.0021 | 3.58E-09 |
| Body mass index | rs562664 | 11:63823619 | T | C | 0.1873 | -0.0138 | 0.0022 | 4.15E-10 |
| Body mass index | rs7102454 | 11:65594820 | T | C | 0.6437 | -0.0168 | 0.0018 | 3.84E-21 |
| Body mass index | rs592483 | 11:69445173 | T | C | 0.5933 | -0.0137 | 0.0017 | 1.53E-16 |
| Body mass index | rs12282785 | 11:76476030 | A | C | 0.2204 | -0.0157 | 0.0023 | 1.43E-11 |
| Body mass index | rs349088 | 11:84814393 | A | C | 0.4777 | -0.013 | 0.0017 | 3.50E-14 |
| Body mass index | rs4256980 | 11:8673939 | C | G | 0.3305 | -0.0187 | 0.0017 | 8.63E-29 |
| Body mass index | rs12421848 | 11:891338 | A | G | 0.3962 | -0.0141 | 0.0019 | 3.99E-13 |
| Body mass index | rs10741329 | 11:89997796 | A | G | 0.6944 | 0.0115 | 0.0018 | 3.91E-10 |
| Body mass index | rs3019466 | 11:92476178 | T | C | 0.1677 | -0.0128 | 0.0024 | 4.91E-08 |
| Body mass index | rs2605603 | 11:93221105 | A | G | 0.4812 | -0.0103 | 0.0016 | 2.04E-10 |
| Body mass index | rs6539064 | 12:103706754 | C | G | 0.7468 | 0.0194 | 0.0019 | 1.14E-23 |
| Body mass index | rs1860561 | 12:110783241 | A | G | 0.2087 | 0.0159 | 0.0019 | 1.71E-16 |
| Body mass index | rs11066188 | 12:112610714 | A | G | 0.3762 | -0.0114 | 0.0016 | 3.05E-12 |
| Body mass index | rs2707183 | 12:116957607 | T | G | 0.5321 | -0.0093 | 0.0017 | 4.89E-08 |
| Body mass index | rs11615578 | 12:121714935 | T | C | 0.2575 | 0.0117 | 0.002 | 3.04E-09 |
| Body mass index | rs12369179 | 12:122963550 | T | C | 0.085 | -0.034 | 0.0031 | 2.32E-28 |
| Body mass index | rs11060853 | 12:123424071 | A | G | 0.591 | -0.0107 | 0.0019 | 3.10E-08 |
| Body mass index | rs11614340 | 12:133426483 | T | C | 0.7066 | -0.0117 | 0.0018 | 1.87E-10 |
| Body mass index | rs10772983 | 12:17141582 | T | C | 0.5394 | -0.01 | 0.0016 | 6.21E-10 |
| Body mass index | rs11044430 | 12:19287416 | A | T | 0.844 | 0.0161 | 0.0023 | 3.77E-12 |
| Body mass index | rs765125 | 12:2156207 | T | C | 0.5855 | -0.0098 | 0.0017 | 1.27E-08 |
| Body mass index | rs11046972 | 12:23705969 | T | C | 0.0714 | 0.0176 | 0.0032 | 3.72E-08 |
| Body mass index | rs10842240 | 12:24060075 | C | G | 0.1256 | 0.0195 | 0.0025 | 1.21E-14 |
| Body mass index | rs11170468 | 12:39430048 | A | C | 0.7826 | 0.013 | 0.0019 | 1.12E-11 |
| Body mass index | rs1350430 | 12:41819215 | T | C | 0.5328 | -0.0125 | 0.0017 | 2.08E-13 |
| Body mass index | rs1126930 | 12:49399132 | C | G | 0.0339 | 0.0341 | 0.0053 | 1.00E-10 |
| Body mass index | rs7138803 | 12:50247468 | A | G | 0.387 | 0.0297 | 0.0017 | 3.10E-71 |
| Body mass index | rs2271189 | 12:56494991 | A | G | 0.396 | -0.0141 | 0.0018 | 9.26E-16 |
| Body mass index | rs7975187 | 12:60964108 | A | G | 0.7712 | -0.0137 | 0.0021 | 3.86E-11 |
| Body mass index | rs650198 | 12:69674595 | T | C | 0.7294 | -0.0137 | 0.0019 | 6.43E-13 |
| Body mass index | rs11115176 | 12:82465797 | T | C | 0.7839 | 0.0131 | 0.0019 | 6.79E-12 |
| Body mass index | rs10506971 | 12:89757937 | A | G | 0.5521 | -0.0142 | 0.0017 | 4.69E-17 |
| Body mass index | rs11105839 | 12:91237920 | A | T | 0.3729 | -0.0113 | 0.0017 | 1.25E-11 |
| Body mass index | rs11611246 | 12:939480 | T | G | 0.199 | 0.0223 | 0.002 | 2.04E-28 |
| Body mass index | rs2712665 | 12:99594947 | T | C | 0.6968 | -0.0108 | 0.0019 | 5.40E-09 |
| Body mass index | rs9888533 | 13:107854612 | T | C | 0.5302 | 0.0125 | 0.0019 | 1.38E-10 |
| Body mass index | rs9522183 | 13:111977280 | T | G | 0.5492 | -0.0135 | 0.0019 | 2.10E-12 |
| Body mass index | rs12868881 | 13:112218924 | A | T | 0.4066 | 0.0138 | 0.0017 | 1.40E-15 |
| Body mass index | rs9512648 | 13:27933910 | A | G | 0.4741 | 0.0095 | 0.0017 | 4.11E-08 |
| Body mass index | rs7323 | 13:28009031 | C | G | 0.268 | -0.0166 | 0.0019 | 2.18E-18 |
| Body mass index | rs4771218 | 13:28655311 | A | G | 0.6244 | -0.0141 | 0.0018 | 3.18E-15 |
| Body mass index | rs9595908 | 13:33184288 | T | C | 0.6427 | 0.0154 | 0.0017 | 3.73E-20 |
| Body mass index | rs9603697 | 13:40783323 | T | C | 0.3184 | 0.0134 | 0.0018 | 1.69E-13 |
| Body mass index | rs12429545 | 13:54102206 | A | G | 0.1218 | 0.0313 | 0.0024 | 1.42E-37 |
| Body mass index | rs962796 | 13:54385284 | T | C | 0.1937 | 0.014 | 0.0021 | 1.88E-11 |
| Body mass index | rs9569777 | 13:58484786 | T | G | 0.1865 | -0.0201 | 0.0021 | 3.43E-21 |
| Body mass index | rs9527895 | 13:59367767 | T | C | 0.819 | -0.0158 | 0.0023 | 3.69E-12 |
| Body mass index | rs8181823 | 13:65477940 | A | C | 0.2344 | -0.0125 | 0.002 | 4.36E-10 |
| Body mass index | rs9599161 | 13:67434016 | T | C | 0.57 | 0.0098 | 0.0016 | 2.32E-09 |
| Body mass index | rs1441264 | 13:79580919 | A | G | 0.5846 | 0.0174 | 0.0017 | 7.60E-25 |
| Body mass index | rs9531786 | 13:85983968 | C | G | 0.3715 | -0.0111 | 0.0018 | 4.11E-10 |
| Body mass index | rs77432547 | 13:86494817 | A | G | 0.7229 | -0.017 | 0.0021 | 1.44E-15 |
| Body mass index | rs1927790 | 13:96922191 | T | C | 0.606 | -0.014 | 0.0016 | 1.57E-17 |
| Body mass index | rs9168 | 13:99101583 | A | C | 0.2752 | -0.0139 | 0.0019 | 1.08E-13 |
| Body mass index | rs7161194 | 14:101529005 | A | G | 0.3437 | 0.019 | 0.0019 | 2.23E-24 |
| Body mass index | rs10145749 | 14:102782109 | T | C | 0.1549 | 0.0161 | 0.0026 | 8.33E-10 |
| Body mass index | rs4906263 | 14:103249127 | C | G | 0.6562 | -0.0176 | 0.0018 | 8.11E-23 |
| Body mass index | rs2273175 | 14:104160141 | T | C | 0.6791 | -0.0121 | 0.0018 | 3.69E-11 |
| Body mass index | rs10132280 | 14:25928179 | A | C | 0.311 | -0.0214 | 0.0018 | 2.28E-33 |
| Body mass index | rs4981693 | 14:29680331 | A | G | 0.7743 | 0.0202 | 0.002 | 7.89E-24 |
| Body mass index | rs225882 | 14:30480123 | T | C | 0.7513 | 0.0113 | 0.0018 | 8.56E-10 |
| Body mass index | rs1958898 | 14:40886886 | C | G | 0.2098 | -0.0146 | 0.0021 | 4.23E-12 |
| Body mass index | rs61983990 | 14:41475003 | A | G | 0.0802 | 0.0198 | 0.0035 | 1.91E-08 |
| Body mass index | rs4900714 | 14:47302219 | T | G | 0.4776 | 0.015 | 0.0017 | 1.00E-18 |
| Body mass index | rs217669 | 14:62360075 | T | C | 0.7224 | -0.0172 | 0.0021 | 6.27E-16 |
| Body mass index | rs4430672 | 14:63094407 | T | C | 0.1958 | 0.0124 | 0.0021 | 5.00E-09 |
| Body mass index | rs3902951 | 14:69789755 | T | G | 0.7691 | -0.0134 | 0.0019 | 2.88E-12 |
| Body mass index | rs17182027 | 14:73348130 | A | G | 0.5647 | -0.011 | 0.0017 | 1.46E-10 |
| Body mass index | rs7144011 | 14:79940383 | T | G | 0.2334 | 0.0263 | 0.002 | 2.37E-40 |
| Body mass index | rs4517716 | 14:83013876 | C | G | 0.7924 | -0.0117 | 0.002 | 4.27E-09 |
| Body mass index | rs12888545 | 14:88308044 | A | G | 0.7464 | -0.0133 | 0.002 | 1.76E-11 |
| Body mass index | rs1951455 | 14:91512339 | T | C | 0.2813 | -0.0148 | 0.0019 | 6.05E-15 |
| Body mass index | rs942066 | 14:94031914 | A | G | 0.373 | -0.0202 | 0.002 | 2.36E-24 |
| Body mass index | rs12594043 | 15:27034988 | C | G | 0.5205 | 0.0102 | 0.0017 | 1.31E-09 |
| Body mass index | rs7172627 | 15:31877690 | A | G | 0.523 | -0.0114 | 0.0017 | 1.81E-11 |
| Body mass index | rs11636611 | 15:36391965 | T | C | 0.4981 | 0.0104 | 0.0017 | 8.85E-10 |
| Body mass index | rs9944219 | 15:46500612 | A | G | 0.6044 | -0.0119 | 0.0017 | 8.14E-13 |
| Body mass index | rs12912198 | 15:47103953 | T | C | 0.2731 | -0.01 | 0.0018 | 3.44E-08 |
| Body mass index | rs6493498 | 15:51754451 | T | C | 0.4465 | 0.0137 | 0.0016 | 4.84E-17 |
| Body mass index | rs10518694 | 15:53072673 | A | C | 0.1394 | 0.0144 | 0.0024 | 3.43E-09 |
| Body mass index | rs12439632 | 15:59103963 | C | G | 0.1682 | 0.0145 | 0.0023 | 3.68E-10 |
| Body mass index | rs339991 | 15:60913637 | A | G | 0.4201 | -0.0125 | 0.0017 | 3.51E-13 |
| Body mass index | rs1559673 | 15:62156514 | A | C | 0.9688 | 0.0359 | 0.0049 | 2.21E-13 |
| Body mass index | rs2241423 | 15:68086838 | A | G | 0.2285 | -0.0298 | 0.0019 | 3.60E-54 |
| Body mass index | rs7171864 | 15:73227249 | A | G | 0.6935 | 0.0132 | 0.0017 | 2.60E-14 |
| Body mass index | rs2470893 | 15:75019449 | T | C | 0.3049 | 0.0107 | 0.0017 | 9.43E-10 |
| Body mass index | rs56151256 | 15:78024806 | A | C | 0.7555 | 0.0166 | 0.0022 | 6.19E-14 |
| Body mass index | rs12914623 | 15:80993570 | C | G | 0.2722 | -0.0159 | 0.0019 | 2.00E-16 |
| Body mass index | rs72757415 | 15:92572762 | T | G | 0.2123 | -0.0159 | 0.0023 | 1.34E-11 |
| Body mass index | rs11633626 | 15:95271378 | A | C | 0.6313 | -0.0157 | 0.0018 | 7.27E-19 |
| Body mass index | rs6496248 | 15:98262923 | A | T | 0.6352 | 0.0104 | 0.0018 | 2.93E-09 |
| Body mass index | rs12591120 | 15:99236869 | T | C | 0.7397 | 0.012 | 0.0022 | 3.84E-08 |
| Body mass index | rs2715423 | 15:99511873 | A | G | 0.2805 | -0.0115 | 0.0019 | 1.90E-09 |
| Body mass index | rs9926784 | 16:19941968 | T | C | 0.8007 | 0.0237 | 0.0021 | 1.08E-30 |
| Body mass index | rs194809 | 16:23804956 | A | G | 0.1871 | 0.0126 | 0.0022 | 4.86E-09 |
| Body mass index | rs2342892 | 16:24540806 | T | G | 0.4855 | 0.0126 | 0.0017 | 1.33E-13 |
| Body mass index | rs7498665 | 16:28883241 | A | G | 0.6179 | -0.0285 | 0.0017 | 1.14E-66 |
| Body mass index | rs3814883 | 16:29994922 | T | C | 0.4795 | 0.0227 | 0.0017 | 1.47E-40 |
| Body mass index | rs214249 | 16:348687 | T | G | 0.6071 | 0.0138 | 0.0017 | 2.72E-15 |
| Body mass index | rs12448257 | 16:3599655 | A | G | 0.2185 | 0.0161 | 0.002 | 9.04E-16 |
| Body mass index | rs879620 | 16:4015729 | T | C | 0.5966 | 0.0226 | 0.0017 | 8.45E-39 |
| Body mass index | rs6500208 | 16:49011249 | A | G | 0.2021 | 0.0146 | 0.002 | 3.21E-13 |
| Body mass index | rs1477199 | 16:53712135 | A | G | 0.8426 | -0.0221 | 0.0023 | 5.15E-21 |
| Body mass index | rs7206790 | 16:53797908 | C | G | 0.5232 | -0.0606 | 0.0017 | ###### |
| Body mass index | rs8057911 | 16:54143352 | T | C | 0.234 | 0.0125 | 0.0023 | 3.66E-08 |
| Body mass index | rs2058527 | 16:6704749 | T | G | 0.2715 | -0.0115 | 0.0019 | 1.80E-09 |
| Body mass index | rs889398 | 16:69556715 | T | C | 0.4151 | -0.0195 | 0.0016 | 3.23E-32 |
| Body mass index | rs35949039 | 16:70572605 | T | G | 0.1056 | -0.0215 | 0.0031 | 6.30E-12 |
| Body mass index | rs12926250 | 16:72213316 | T | G | 0.1051 | 0.018 | 0.0028 | 1.78E-10 |
| Body mass index | rs756717 | 16:72996162 | A | G | 0.394 | -0.0134 | 0.0017 | 2.38E-15 |
| Body mass index | rs4390583 | 16:81694835 | A | C | 0.5714 | -0.01 | 0.0018 | 1.63E-08 |
| Body mass index | rs12922346 | 16:82438337 | C | G | 0.2641 | 0.0133 | 0.002 | 1.48E-11 |
| Body mass index | rs7206608 | 16:82872628 | C | G | 0.6751 | -0.013 | 0.0018 | 1.20E-12 |
| Body mass index | rs1075901 | 17:15943910 | T | C | 0.4516 | -0.0118 | 0.0016 | 4.43E-13 |
| Body mass index | rs3923783 | 17:1843189 | A | C | 0.1784 | -0.0222 | 0.0022 | 4.12E-23 |
| Body mass index | rs4986044 | 17:21261560 | T | C | 0.455 | -0.0177 | 0.0016 | 1.27E-27 |
| Body mass index | rs11078883 | 17:2138828 | C | G | 0.654 | -0.0126 | 0.0018 | 1.30E-12 |
| Body mass index | rs8065172 | 17:31456969 | A | G | 0.2383 | -0.0124 | 0.002 | 5.29E-10 |
| Body mass index | rs12150665 | 17:34914787 | T | C | 0.5931 | 0.0168 | 0.0016 | 1.74E-24 |
| Body mass index | rs6607337 | 17:35057373 | T | C | 0.2978 | -0.0124 | 0.0019 | 2.55E-11 |
| Body mass index | rs56161855 | 17:46288649 | A | T | 0.8678 | -0.0233 | 0.0028 | 1.41E-16 |
| Body mass index | rs9299 | 17:46669430 | T | C | 0.6447 | 0.0119 | 0.0018 | 1.87E-11 |
| Body mass index | rs11655587 | 17:47140794 | T | C | 0.3572 | -0.021 | 0.002 | 6.87E-26 |
| Body mass index | rs7774 | 17:4801163 | A | C | 0.3197 | 0.0128 | 0.0021 | 4.22E-10 |
| Body mass index | rs11649864 | 17:56093061 | A | G | 0.0859 | 0.0192 | 0.003 | 2.52E-10 |
| Body mass index | rs12602912 | 17:65870073 | T | C | 0.212 | 0.0166 | 0.002 | 2.90E-16 |
| Body mass index | rs312750 | 17:68343539 | A | G | 0.4986 | 0.0097 | 0.0016 | 2.57E-09 |
| Body mass index | rs12939549 | 17:78611724 | A | G | 0.5572 | 0.018 | 0.0016 | 3.68E-28 |
| Body mass index | rs35867081 | 17:79047278 | A | G | 0.4871 | 0.015 | 0.0019 | 6.65E-15 |
| Body mass index | rs8097672 | 18:1839601 | A | T | 0.8545 | -0.0209 | 0.0024 | 3.26E-18 |
| Body mass index | rs891387 | 18:21103909 | T | C | 0.4951 | 0.0208 | 0.0017 | 9.26E-35 |
| Body mass index | rs1945160 | 18:22164216 | A | G | 0.3819 | -0.0104 | 0.0018 | 5.65E-09 |
| Body mass index | rs1941696 | 18:31252129 | A | G | 0.5174 | 0.0112 | 0.0017 | 4.74E-11 |
| Body mass index | rs474605 | 18:39612720 | A | G | 0.4679 | -0.0125 | 0.0017 | 2.72E-13 |
| Body mass index | rs1356506 | 18:40708038 | T | C | 0.6287 | 0.0137 | 0.0018 | 8.39E-15 |
| Body mass index | rs2612576 | 18:42967950 | A | T | 0.2945 | -0.0108 | 0.0019 | 8.49E-09 |
| Body mass index | rs9951619 | 18:56882326 | T | G | 0.2239 | -0.0152 | 0.0019 | 2.33E-15 |
| Body mass index | rs6567160 | 18:57829135 | T | C | 0.7518 | -0.0552 | 0.0019 | ###### |
| Body mass index | rs17066856 | 18:58049656 | T | C | 0.8915 | 0.035 | 0.0027 | 7.10E-38 |
| Body mass index | rs66595146 | 18:58204315 | A | C | 0.6291 | 0.0135 | 0.002 | 9.09E-12 |
| Body mass index | rs17783165 | 18:63461638 | T | C | 0.671 | -0.0128 | 0.0017 | 2.74E-13 |
| Body mass index | rs11150911 | 18:73498528 | A | C | 0.2848 | 0.0118 | 0.0018 | 3.72E-11 |
| Body mass index | rs478707 | 18:7543207 | T | C | 0.2032 | -0.015 | 0.0024 | 1.98E-10 |
| Body mass index | rs273512 | 19:18224729 | T | C | 0.409 | 0.0156 | 0.0018 | 4.48E-19 |
| Body mass index | rs17724992 | 19:18454825 | A | G | 0.7197 | 0.0172 | 0.0018 | 5.23E-21 |
| Body mass index | rs12981256 | 19:1865901 | A | G | 0.5284 | 0.0151 | 0.0017 | 1.43E-18 |
| Body mass index | rs7258722 | 19:18808915 | A | T | 0.4131 | -0.02 | 0.0017 | 1.81E-30 |
| Body mass index | rs12611148 | 19:19865077 | A | C | 0.1429 | -0.0137 | 0.0024 | 1.42E-08 |
| Body mass index | rs45486197 | 19:2244849 | A | G | 0.0635 | 0.0279 | 0.004 | 1.80E-12 |
| Body mass index | rs12462975 | 19:30272202 | A | G | 0.3243 | 0.0193 | 0.0018 | 1.47E-25 |
| Body mass index | rs7245985 | 19:30710410 | T | G | 0.7956 | 0.0119 | 0.0021 | 1.88E-08 |
| Body mass index | rs11882409 | 19:34019685 | A | C | 0.303 | 0.0121 | 0.0019 | 3.31E-10 |
| Body mass index | rs185350 | 19:34306816 | T | C | 0.4902 | 0.0137 | 0.0016 | 9.10E-17 |
| Body mass index | rs895330 | 19:4060707 | C | G | 0.806 | 0.0196 | 0.0022 | 1.59E-19 |
| Body mass index | rs429358 | 19:45411941 | T | C | 0.8459 | 0.0257 | 0.0026 | 2.60E-22 |
| Body mass index | rs11672660 | 19:46180184 | T | C | 0.1871 | -0.0338 | 0.0021 | 6.83E-60 |
| Body mass index | rs9304665 | 19:47602577 | A | T | 0.7423 | 0.023 | 0.002 | 2.20E-31 |
| Body mass index | rs3764625 | 19:49649051 | T | G | 0.3989 | 0.0096 | 0.0017 | 2.42E-08 |
| Body mass index | rs4303732 | 2:100830040 | T | C | 0.6137 | 0.0169 | 0.0017 | 1.26E-22 |
| Body mass index | rs264941 | 2:104297420 | A | C | 0.4679 | -0.0124 | 0.0017 | 1.84E-13 |
| Body mass index | rs10197031 | 2:105454590 | T | C | 0.7262 | -0.0161 | 0.0019 | 5.05E-18 |
| Body mass index | rs11902450 | 2:12845368 | T | C | 0.1052 | 0.0167 | 0.0027 | 1.06E-09 |
| Body mass index | rs13033310 | 2:133523605 | A | G | 0.2519 | 0.0146 | 0.0022 | 3.40E-11 |
| Body mass index | rs4988235 | 2:136608646 | A | G | 0.7206 | 0.0124 | 0.0017 | 7.09E-13 |
| Body mass index | rs17814208 | 2:144037998 | A | G | 0.7575 | -0.0129 | 0.002 | 7.24E-11 |
| Body mass index | rs1451077 | 2:147901207 | A | G | 0.5776 | -0.0169 | 0.0019 | 1.43E-18 |
| Body mass index | rs2119753 | 2:151224579 | A | G | 0.6229 | 0.01 | 0.0017 | 1.00E-08 |
| Body mass index | rs11695013 | 2:157057487 | T | C | 0.632 | -0.0104 | 0.0017 | 2.43E-09 |
| Body mass index | rs3764835 | 2:159519368 | A | G | 0.1505 | -0.013 | 0.0024 | 3.84E-08 |
| Body mass index | rs12692596 | 2:161265910 | T | C | 0.3618 | 0.012 | 0.0017 | 1.03E-12 |
| Body mass index | rs62176243 | 2:166190881 | A | T | 0.7571 | 0.0148 | 0.0022 | 2.41E-11 |
| Body mass index | rs56133507 | 2:172818467 | T | G | 0.8065 | -0.0131 | 0.0024 | 4.31E-08 |
| Body mass index | rs34234296 | 2:175166636 | A | G | 0.3876 | -0.0145 | 0.002 | 2.01E-13 |
| Body mass index | rs7588437 | 2:181575281 | A | G | 0.3664 | -0.0165 | 0.0017 | 2.32E-22 |
| Body mass index | rs6716898 | 2:198944271 | A | G | 0.4873 | 0.0127 | 0.0019 | 2.33E-11 |
| Body mass index | rs7593917 | 2:203931012 | A | G | 0.4535 | -0.0115 | 0.0016 | 9.67E-13 |
| Body mass index | rs4482463 | 2:205375909 | A | C | 0.917 | -0.031 | 0.0031 | 4.85E-23 |
| Body mass index | rs11692326 | 2:208263279 | T | C | 0.2302 | 0.0147 | 0.0019 | 1.69E-14 |
| Body mass index | rs73985439 | 2:212299249 | A | C | 0.6914 | -0.0131 | 0.0021 | 1.84E-10 |
| Body mass index | rs7599312 | 2:213413231 | A | G | 0.2737 | -0.0182 | 0.0018 | 1.52E-23 |
| Body mass index | rs12987009 | 2:219658170 | A | T | 0.5851 | -0.0109 | 0.0017 | 1.83E-10 |
| Body mass index | rs6725931 | 2:220205146 | T | C | 0.8483 | 0.0187 | 0.0024 | 2.30E-15 |
| Body mass index | rs4973618 | 2:229002620 | A | G | 0.6623 | -0.0148 | 0.0018 | 1.25E-16 |
| Body mass index | rs6720868 | 2:230663576 | T | C | 0.3083 | 0.0154 | 0.0018 | 1.82E-17 |
| Body mass index | rs7568228 | 2:236848488 | C | G | 0.5401 | -0.0104 | 0.0017 | 1.00E-09 |
| Body mass index | rs6710091 | 2:239597 | C | G | 0.6655 | 0.0103 | 0.0018 | 5.78E-09 |
| Body mass index | rs10182181 | 2:25150296 | A | G | 0.5052 | -0.0327 | 0.0016 | 2.45E-91 |
| Body mass index | rs935166 | 2:26949366 | A | G | 0.4948 | -0.0152 | 0.0019 | 8.69E-16 |
| Body mass index | rs10168563 | 2:35552173 | A | G | 0.7031 | 0.0126 | 0.0018 | 9.33E-12 |
| Body mass index | rs3770890 | 2:36657992 | T | G | 0.9725 | -0.0297 | 0.0053 | 2.10E-08 |
| Body mass index | rs10185199 | 2:40282202 | A | G | 0.286 | -0.0143 | 0.002 | 3.18E-13 |
| Body mass index | rs10169594 | 2:41637688 | T | C | 0.6475 | -0.012 | 0.0018 | 1.34E-11 |
| Body mass index | rs77165542 | 2:430975 | T | C | 0.0341 | -0.0939 | 0.0053 | 1.51E-70 |
| Body mass index | rs7561278 | 2:48954905 | T | C | 0.7668 | 0.0169 | 0.0021 | 4.89E-16 |
| Body mass index | rs930295 | 2:50233352 | A | C | 0.1548 | 0.0208 | 0.0023 | 2.03E-19 |
| Body mass index | rs805412 | 2:54120820 | A | G | 0.4337 | -0.0098 | 0.0017 | 1.14E-08 |
| Body mass index | rs4671328 | 2:58935282 | T | G | 0.4496 | 0.0214 | 0.0017 | 3.42E-36 |
| Body mass index | rs6545714 | 2:59307725 | A | G | 0.6112 | -0.0194 | 0.0016 | 4.01E-32 |
| Body mass index | rs10929925 | 2:6155557 | A | C | 0.4105 | -0.0142 | 0.0016 | 3.05E-18 |
| Body mass index | rs13021737 | 2:632348 | A | G | 0.1567 | -0.0578 | 0.0021 | ###### |
| Body mass index | rs2861685 | 2:67837553 | T | C | 0.5864 | 0.0165 | 0.0019 | 7.82E-18 |
| Body mass index | rs17020497 | 2:81826131 | A | G | 0.1341 | 0.014 | 0.0025 | 2.04E-08 |
| Body mass index | rs12714199 | 2:86812549 | T | C | 0.6146 | -0.0141 | 0.0017 | 3.22E-16 |
| Body mass index | rs2423668 | 20:12430673 | T | C | 0.4196 | 0.0106 | 0.0018 | 7.84E-09 |
| Body mass index | rs1884389 | 20:1410582 | T | C | 0.4386 | -0.0108 | 0.0017 | 3.72E-10 |
| Body mass index | rs852056 | 20:17102860 | T | C | 0.2538 | 0.0123 | 0.0019 | 2.15E-10 |
| Body mass index | rs1409818 | 20:21381121 | T | C | 0.1074 | 0.0195 | 0.0028 | 2.59E-12 |
| Body mass index | rs8122855 | 20:25192049 | A | G | 0.3377 | 0.0137 | 0.0018 | 4.11E-14 |
| Body mass index | rs4813619 | 20:2815715 | T | G | 0.5305 | -0.0106 | 0.0018 | 1.70E-09 |
| Body mass index | rs4812405 | 20:35276585 | A | C | 0.0763 | -0.0202 | 0.0033 | 1.28E-09 |
| Body mass index | rs742748 | 20:39293397 | T | C | 0.5864 | -0.0113 | 0.0017 | 1.13E-11 |
| Body mass index | rs79186842 | 20:47689036 | A | G | 0.8597 | 0.0202 | 0.0028 | 2.60E-13 |
| Body mass index | rs17806224 | 20:51065854 | A | G | 0.1808 | -0.026 | 0.0022 | 7.91E-32 |
| Body mass index | rs1304549 | 20:54378256 | A | G | 0.2262 | -0.0118 | 0.0021 | 1.35E-08 |
| Body mass index | rs6010784 | 20:61540319 | T | C | 0.5062 | 0.0106 | 0.0016 | 6.91E-11 |
| Body mass index | rs6512302 | 20:62691550 | C | G | 0.7391 | 0.0134 | 0.002 | 1.52E-11 |
| Body mass index | rs1884897 | 20:6612832 | A | G | 0.3747 | -0.0184 | 0.0017 | 2.70E-28 |
| Body mass index | rs2064044 | 21:22119890 | A | C | 0.8062 | -0.0123 | 0.0021 | 1.02E-08 |
| Body mass index | rs2832283 | 21:30690558 | A | G | 0.2254 | 0.0115 | 0.002 | 4.72E-09 |
| Body mass index | rs13047416 | 21:40309436 | C | G | 0.6263 | 0.0152 | 0.0018 | 6.93E-18 |
| Body mass index | rs8134638 | 21:40644170 | T | C | 0.6273 | -0.0133 | 0.002 | 1.57E-11 |
| Body mass index | rs8126575 | 21:46435610 | T | G | 0.8673 | 0.0152 | 0.0025 | 5.88E-10 |
| Body mass index | rs427943 | 21:46570896 | A | C | 0.4288 | -0.0177 | 0.0017 | 3.60E-25 |
| Body mass index | rs11538 | 22:18220831 | A | G | 0.8284 | -0.0138 | 0.0023 | 1.09E-09 |
| Body mass index | rs2238799 | 22:20109325 | A | G | 0.6187 | 0.0102 | 0.0018 | 8.65E-09 |
| Body mass index | rs12628891 | 22:38317137 | T | C | 0.3186 | -0.0115 | 0.0019 | 5.85E-10 |
| Body mass index | rs12628051 | 22:40654276 | T | C | 0.6407 | 0.0161 | 0.0018 | 2.90E-19 |
| Body mass index | rs28489620 | 22:41804716 | A | G | 0.2818 | -0.0151 | 0.0021 | 1.42E-12 |
| Body mass index | rs9615337 | 22:48898559 | C | G | 0.575 | -0.011 | 0.002 | 2.05E-08 |
| Body mass index | rs1436344 | 3:104606144 | C | G | 0.5703 | 0.0147 | 0.0017 | 1.07E-17 |
| Body mass index | rs7640424 | 3:107820063 | T | C | 0.3106 | -0.0135 | 0.0018 | 1.23E-14 |
| Body mass index | rs17681451 | 3:114399296 | A | G | 0.0762 | -0.0225 | 0.0031 | 7.37E-13 |
| Body mass index | rs6783054 | 3:11672805 | A | C | 0.5028 | 0.0099 | 0.0017 | 6.22E-09 |
| Body mass index | rs6808814 | 3:116852469 | T | C | 0.7335 | 0.0118 | 0.0019 | 1.31E-09 |
| Body mass index | rs10510419 | 3:12426936 | T | G | 0.1467 | -0.0168 | 0.0023 | 2.23E-13 |
| Body mass index | rs2600226 | 3:12928762 | T | C | 0.6712 | -0.0116 | 0.0018 | 1.42E-10 |
| Body mass index | rs7631156 | 3:131751628 | A | G | 0.3091 | 0.0215 | 0.0018 | 3.33E-32 |
| Body mass index | rs1048637 | 3:13358171 | T | G | 0.5474 | -0.0094 | 0.0017 | 2.94E-08 |
| Body mass index | rs687339 | 3:135932359 | T | C | 0.7697 | 0.0188 | 0.0019 | 4.32E-22 |
| Body mass index | rs16851483 | 3:141275436 | T | G | 0.0717 | 0.0352 | 0.0034 | 4.87E-25 |
| Body mass index | rs355777 | 3:154034950 | C | G | 0.3984 | 0.0151 | 0.0017 | 2.13E-18 |
| Body mass index | rs9826775 | 3:156295341 | A | G | 0.8513 | 0.0155 | 0.0024 | 6.61E-11 |
| Body mass index | rs2047648 | 3:157033438 | A | T | 0.7465 | -0.0133 | 0.002 | 7.95E-12 |
| Body mass index | rs3732927 | 3:170586057 | T | C | 0.2907 | 0.0099 | 0.0017 | 1.13E-08 |
| Body mass index | rs12635553 | 3:171113714 | A | T | 0.486 | 0.0099 | 0.0017 | 4.10E-09 |
| Body mass index | rs39654 | 3:173095123 | A | G | 0.4513 | -0.0163 | 0.0017 | 1.75E-21 |
| Body mass index | rs6443750 | 3:181329682 | T | C | 0.1994 | -0.0152 | 0.0021 | 7.25E-13 |
| Body mass index | rs865809 | 3:183997735 | A | G | 0.2246 | 0.0124 | 0.002 | 6.50E-10 |
| Body mass index | rs9816226 | 3:185834499 | A | T | 0.1755 | -0.0315 | 0.0021 | 1.45E-50 |
| Body mass index | rs7616009 | 3:194881756 | A | G | 0.1595 | -0.0157 | 0.0024 | 4.33E-11 |
| Body mass index | rs6803161 | 3:196205694 | T | C | 0.3945 | 0.0107 | 0.0019 | 2.71E-08 |
| Body mass index | rs4858193 | 3:20441050 | T | C | 0.7199 | 0.0133 | 0.0019 | 2.44E-12 |
| Body mass index | rs6804842 | 3:25106437 | A | G | 0.4301 | -0.0141 | 0.0016 | 7.57E-18 |
| Body mass index | rs11921432 | 3:35117776 | T | C | 0.8906 | -0.0189 | 0.0027 | 5.38E-12 |
| Body mass index | rs1799923 | 3:42306294 | A | G | 0.1117 | -0.0224 | 0.0026 | 1.12E-17 |
| Body mass index | rs28350 | 3:42418446 | A | G | 0.1717 | 0.0172 | 0.0022 | 1.07E-14 |
| Body mass index | rs4017425 | 3:44028764 | T | C | 0.4676 | -0.0118 | 0.0017 | 2.91E-12 |
| Body mass index | rs11919665 | 3:48085349 | A | T | 0.3249 | 0.0117 | 0.002 | 7.30E-09 |
| Body mass index | rs11713193 | 3:49924424 | A | G | 0.5156 | 0.0246 | 0.0017 | 3.02E-48 |
| Body mass index | rs2365389 | 3:61236462 | T | C | 0.3999 | -0.0168 | 0.0016 | 6.49E-25 |
| Body mass index | rs6445258 | 3:62112198 | T | C | 0.207 | 0.0131 | 0.0023 | 2.48E-08 |
| Body mass index | rs925018 | 3:62713143 | C | G | 0.6665 | -0.013 | 0.0017 | 4.33E-14 |
| Body mass index | rs11915371 | 3:70539559 | A | C | 0.8019 | -0.0154 | 0.0021 | 2.29E-13 |
| Body mass index | rs12636480 | 3:82719412 | T | G | 0.3507 | 0.0128 | 0.0018 | 6.96E-13 |
| Body mass index | rs9827823 | 3:84221774 | T | C | 0.8552 | 0.0182 | 0.0023 | 3.39E-15 |
| Body mass index | rs9818122 | 3:85861064 | T | C | 0.7933 | -0.0228 | 0.002 | 3.97E-30 |
| Body mass index | rs34184235 | 3:86192846 | T | C | 0.4353 | -0.0115 | 0.0019 | 2.14E-09 |
| Body mass index | rs11128021 | 3:88139016 | A | G | 0.1615 | -0.0181 | 0.0024 | 1.63E-14 |
| Body mass index | rs1492014 | 3:94071481 | T | C | 0.5674 | -0.0171 | 0.0017 | 1.44E-23 |
| Body mass index | rs59302296 | 3:9507314 | A | T | 0.0991 | 0.0217 | 0.0032 | 9.12E-12 |
| Body mass index | rs13107325 | 4:103188709 | T | C | 0.082 | 0.0468 | 0.0032 | 3.81E-47 |
| Body mass index | rs326893 | 4:112691776 | T | C | 0.5812 | 0.0121 | 0.0017 | 1.89E-12 |
| Body mass index | rs7696649 | 4:120322177 | A | G | 0.278 | 0.0116 | 0.0019 | 4.73E-10 |
| Body mass index | rs4864201 | 4:130731284 | T | C | 0.3471 | 0.0137 | 0.0017 | 4.30E-16 |
| Body mass index | rs1296328 | 4:137083193 | A | C | 0.4464 | 0.0166 | 0.0017 | 3.49E-22 |
| Body mass index | rs17367750 | 4:140782542 | T | C | 0.3117 | -0.0122 | 0.0018 | 1.97E-11 |
| Body mass index | rs9992189 | 4:144060728 | C | G | 0.6083 | -0.0097 | 0.0017 | 2.04E-08 |
| Body mass index | rs3914628 | 4:147438019 | T | C | 0.8593 | 0.0165 | 0.0023 | 6.91E-13 |
| Body mass index | rs750090 | 4:152931436 | T | C | 0.6258 | 0.0113 | 0.0018 | 2.50E-10 |
| Body mass index | rs13110266 | 4:162129844 | A | G | 0.4037 | -0.0124 | 0.0016 | 3.96E-14 |
| Body mass index | rs7685628 | 4:165310133 | A | T | 0.4014 | 0.0101 | 0.0017 | 6.15E-09 |
| Body mass index | rs12642970 | 4:16601492 | C | G | 0.44 | -0.0109 | 0.0019 | 1.71E-08 |
| Body mass index | rs1522569 | 4:171632637 | T | G | 0.8183 | 0.0141 | 0.0022 | 1.60E-10 |
| Body mass index | rs1437842 | 4:173597016 | A | G | 0.4907 | -0.0106 | 0.0017 | 8.49E-10 |
| Body mass index | rs6850421 | 4:180187034 | A | G | 0.4564 | 0.0113 | 0.0019 | 3.66E-09 |
| Body mass index | rs994596 | 4:18459828 | T | C | 0.3247 | 0.013 | 0.0018 | 1.06E-12 |
| Body mass index | rs35408866 | 4:187743245 | A | G | 0.1339 | 0.0159 | 0.0028 | 1.50E-08 |
| Body mass index | rs34811474 | 4:25408838 | A | G | 0.2224 | -0.0293 | 0.0023 | 8.50E-38 |
| Body mass index | rs73213484 | 4:28489339 | A | T | 0.8565 | 0.0208 | 0.0027 | 2.04E-14 |
| Body mass index | rs2051559 | 4:3298800 | T | C | 0.8588 | -0.0167 | 0.0025 | 3.78E-11 |
| Body mass index | rs337637 | 4:38604470 | A | G | 0.359 | -0.0137 | 0.0017 | 6.24E-16 |
| Body mass index | rs10938397 | 4:45182527 | A | G | 0.57 | -0.0322 | 0.0016 | 2.42E-86 |
| Body mass index | rs2271046 | 4:52752812 | A | T | 0.6948 | -0.0115 | 0.0018 | 3.03E-10 |
| Body mass index | rs1492767 | 4:55221467 | T | C | 0.4681 | 0.0095 | 0.0016 | 3.55E-09 |
| Body mass index | rs2192158 | 4:55505360 | A | G | 0.4492 | 0.0137 | 0.0017 | 7.42E-16 |
| Body mass index | rs1346841 | 4:65651730 | A | G | 0.4116 | -0.0126 | 0.0017 | 3.18E-13 |
| Body mass index | rs10002111 | 4:67815504 | A | G | 0.2227 | 0.0125 | 0.0021 | 1.26E-09 |
| Body mass index | rs10033843 | 4:77028783 | A | G | 0.215 | 0.014 | 0.0021 | 1.07E-11 |
| Body mass index | rs72649373 | 4:80609966 | T | C | 0.8619 | -0.0167 | 0.0028 | 3.20E-09 |
| Body mass index | rs4148155 | 4:89054667 | A | G | 0.8867 | 0.0192 | 0.0026 | 1.34E-13 |
| Body mass index | rs4286488 | 4:94440026 | A | G | 0.7608 | 0.0121 | 0.002 | 1.70E-09 |
| Body mass index | rs7678054 | 4:95093855 | A | G | 0.4788 | -0.0099 | 0.0017 | 4.57E-09 |
| Body mass index | rs3796432 | 4:96030402 | T | G | 0.3672 | -0.0113 | 0.0018 | 2.21E-10 |
| Body mass index | rs11739877 | 5:105876806 | T | C | 0.6231 | 0.0116 | 0.0018 | 4.01E-11 |
| Body mass index | rs6888194 | 5:106910657 | T | C | 0.8443 | -0.0127 | 0.0023 | 3.58E-08 |
| Body mass index | rs40067 | 5:107439012 | A | G | 0.1701 | -0.0252 | 0.0023 | 9.73E-29 |
| Body mass index | rs459552 | 5:112176756 | A | T | 0.7747 | -0.0133 | 0.0019 | 8.36E-12 |
| Body mass index | rs6893539 | 5:122705737 | A | C | 0.6981 | -0.0122 | 0.0019 | 6.17E-11 |
| Body mass index | rs6864049 | 5:124330522 | A | G | 0.489 | -0.0121 | 0.0016 | 1.48E-13 |
| Body mass index | rs6886072 | 5:136598460 | T | C | 0.4665 | -0.01 | 0.0017 | 4.13E-09 |
| Body mass index | rs13174863 | 5:139080745 | A | G | 0.8542 | -0.0197 | 0.0023 | 1.94E-17 |
| Body mass index | rs17405603 | 5:144608340 | A | T | 0.7062 | -0.0125 | 0.0019 | 4.43E-11 |
| Body mass index | rs2910026 | 5:152529936 | T | C | 0.7222 | -0.0132 | 0.0021 | 5.69E-10 |
| Body mass index | rs7715256 | 5:153537893 | T | G | 0.5644 | -0.0158 | 0.0016 | 3.98E-22 |
| Body mass index | rs7734385 | 5:158460212 | A | G | 0.4449 | -0.0101 | 0.0016 | 6.08E-10 |
| Body mass index | rs4921301 | 5:159984492 | T | C | 0.2127 | -0.0129 | 0.0024 | 4.03E-08 |
| Body mass index | rs2861089 | 5:164557954 | A | T | 0.3806 | 0.0105 | 0.0017 | 1.55E-09 |
| Body mass index | rs7727781 | 5:165185571 | T | C | 0.5164 | 0.0093 | 0.0017 | 4.27E-08 |
| Body mass index | rs2053682 | 5:170599327 | A | C | 0.6784 | 0.017 | 0.0018 | 2.59E-20 |
| Body mass index | rs6556301 | 5:176527577 | T | G | 0.3707 | -0.0113 | 0.0017 | 8.14E-11 |
| Body mass index | rs6890310 | 5:27193573 | A | G | 0.2923 | -0.0119 | 0.0019 | 3.29E-10 |
| Body mass index | rs698147 | 5:3513485 | A | G | 0.447 | 0.0116 | 0.0017 | 9.67E-12 |
| Body mass index | rs7730004 | 5:43191033 | T | C | 0.6614 | 0.0139 | 0.0018 | 1.46E-14 |
| Body mass index | rs116374395 | 5:50723410 | A | G | 0.0348 | 0.0321 | 0.0052 | 7.05E-10 |
| Body mass index | rs150215901 | 5:50935903 | A | T | 0.0414 | -0.0275 | 0.0049 | 1.54E-08 |
| Body mass index | rs4865796 | 5:53272664 | A | G | 0.7044 | -0.0096 | 0.0018 | 4.21E-08 |
| Body mass index | rs13186194 | 5:60795485 | T | C | 0.6169 | 0.01 | 0.0017 | 2.20E-09 |
| Body mass index | rs4700608 | 5:63026280 | T | C | 0.5236 | -0.0155 | 0.0017 | 4.32E-20 |
| Body mass index | rs10050620 | 5:63927239 | T | C | 0.3308 | -0.013 | 0.002 | 1.20E-10 |
| Body mass index | rs1159692 | 5:63977815 | A | C | 0.4752 | 0.0135 | 0.0017 | 5.54E-15 |
| Body mass index | rs2112347 | 5:75015242 | T | G | 0.6298 | 0.0276 | 0.0017 | 1.17E-61 |
| Body mass index | rs10942267 | 5:80841914 | A | G | 0.6927 | 0.0148 | 0.0018 | 6.53E-16 |
| Body mass index | rs2962334 | 5:86879056 | T | G | 0.0272 | 0.0396 | 0.0059 | 1.78E-11 |
| Body mass index | rs1501673 | 5:87963600 | A | G | 0.1355 | 0.0289 | 0.0025 | 2.73E-31 |
| Body mass index | rs12652212 | 5:88808594 | A | G | 0.5611 | -0.0131 | 0.0016 | 1.63E-15 |
| Body mass index | rs7713317 | 5:95716722 | A | G | 0.7198 | -0.0166 | 0.0018 | 1.96E-20 |
| Body mass index | rs6882366 | 5:95864693 | T | C | 0.3959 | -0.0131 | 0.0017 | 4.39E-14 |
| Body mass index | rs57989773 | 6:100629078 | T | C | 0.7576 | -0.0142 | 0.0023 | 5.63E-10 |
| Body mass index | rs156201 | 6:104847441 | C | G | 0.7459 | 0.0124 | 0.0019 | 1.65E-10 |
| Body mass index | rs768023 | 6:108876002 | A | G | 0.6033 | 0.0161 | 0.0016 | 1.13E-22 |
| Body mass index | rs2357760 | 6:120213880 | A | G | 0.6735 | 0.0143 | 0.0017 | 2.11E-16 |
| Body mass index | rs2228213 | 6:12124855 | A | G | 0.3374 | -0.0144 | 0.0017 | 5.50E-17 |
| Body mass index | rs2875762 | 6:124925032 | C | G | 0.252 | 0.0129 | 0.002 | 1.07E-10 |
| Body mass index | rs1268065 | 6:126042783 | A | G | 0.5 | -0.01 | 0.0016 | 7.12E-10 |
| Body mass index | rs9375702 | 6:130384187 | T | C | 0.6785 | -0.0106 | 0.0018 | 5.52E-09 |
| Body mass index | rs11757278 | 6:13180454 | T | C | 0.6961 | 0.0133 | 0.0019 | 6.92E-13 |
| Body mass index | rs2246012 | 6:131898208 | T | C | 0.8512 | -0.0161 | 0.0022 | 1.15E-13 |
| Body mass index | rs6922607 | 6:142703483 | A | G | 0.808 | -0.013 | 0.0022 | 1.85E-09 |
| Body mass index | rs765875 | 6:143185683 | T | C | 0.4689 | -0.0132 | 0.0017 | 1.08E-14 |
| Body mass index | rs7760482 | 6:147354276 | A | G | 0.6159 | -0.0102 | 0.0018 | 6.25E-09 |
| Body mass index | rs12527426 | 6:153392002 | A | G | 0.3 | 0.015 | 0.0019 | 5.54E-16 |
| Body mass index | rs9478496 | 6:154333183 | T | C | 0.8453 | -0.0157 | 0.0023 | 8.17E-12 |
| Body mass index | rs13191362 | 6:163033350 | A | G | 0.8572 | 0.0235 | 0.0025 | 4.08E-21 |
| Body mass index | rs9458814 | 6:163771305 | T | C | 0.7708 | -0.0112 | 0.002 | 2.33E-08 |
| Body mass index | rs3806114 | 6:20482335 | A | G | 0.6916 | -0.012 | 0.0018 | 1.46E-11 |
| Body mass index | rs2066295 | 6:26168903 | A | G | 0.7577 | 0.0142 | 0.002 | 2.29E-12 |
| Body mass index | rs3115667 | 6:31643399 | T | C | 0.2821 | -0.0179 | 0.0019 | 5.92E-21 |
| Body mass index | rs2281819 | 6:33771673 | A | T | 0.2293 | -0.0154 | 0.002 | 2.09E-14 |
| Body mass index | rs2744974 | 6:34579431 | T | C | 0.3239 | 0.0261 | 0.0017 | 1.28E-51 |
| Body mass index | rs2436728 | 6:40365601 | A | G | 0.4073 | 0.0189 | 0.0017 | 1.97E-29 |
| Body mass index | rs1358980 | 6:43764551 | T | C | 0.4735 | -0.0129 | 0.0017 | 5.15E-15 |
| Body mass index | rs2206277 | 6:50798526 | T | C | 0.1557 | 0.0408 | 0.0021 | 1.82E-83 |
| Body mass index | rs1327259 | 6:51177811 | A | G | 0.6096 | 0.0157 | 0.0017 | 1.47E-19 |
| Body mass index | rs6915002 | 6:54028069 | T | C | 0.4113 | 0.0099 | 0.0017 | 8.71E-09 |
| Body mass index | rs9370410 | 6:55171842 | A | G | 0.7293 | 0.0105 | 0.0019 | 2.58E-08 |
| Body mass index | rs2622274 | 6:64240516 | T | G | 0.4553 | -0.0107 | 0.0017 | 3.23E-10 |
| Body mass index | rs1293037 | 6:70248345 | T | C | 0.7544 | 0.0129 | 0.0022 | 5.73E-09 |
| Body mass index | rs6921533 | 6:73742334 | T | C | 0.2933 | 0.0104 | 0.0018 | 1.37E-08 |
| Body mass index | rs9294260 | 6:83433228 | A | G | 0.4689 | 0.014 | 0.0016 | 8.16E-18 |
| Body mass index | rs6909685 | 6:97753952 | T | C | 0.3295 | -0.0149 | 0.0018 | 2.78E-16 |
| Body mass index | rs9320823 | 6:98429337 | T | C | 0.4118 | -0.0165 | 0.0017 | 2.07E-21 |
| Body mass index | rs1721447 | 7:109214139 | T | G | 0.5096 | -0.01 | 0.0017 | 3.79E-09 |
| Body mass index | rs2396625 | 7:113028634 | A | T | 0.4183 | -0.0176 | 0.0017 | 2.81E-24 |
| Body mass index | rs13245051 | 7:113362799 | A | G | 0.4557 | 0.015 | 0.0017 | 1.12E-18 |
| Body mass index | rs10261050 | 7:114337652 | T | C | 0.4754 | 0.0113 | 0.0017 | 4.53E-11 |
| Body mass index | rs2283093 | 7:126721231 | T | C | 0.1949 | 0.0121 | 0.0021 | 1.10E-08 |
| Body mass index | rs6950388 | 7:1270699 | A | G | 0.7782 | 0.0135 | 0.0022 | 1.59E-09 |
| Body mass index | rs7802342 | 7:137435925 | T | G | 0.7048 | -0.0124 | 0.0019 | 6.23E-11 |
| Body mass index | rs11525873 | 7:138817193 | T | C | 0.8995 | 0.0232 | 0.0032 | 2.98E-13 |
| Body mass index | rs11773362 | 7:147668180 | T | C | 0.3396 | -0.0105 | 0.0018 | 6.39E-09 |
| Body mass index | rs2907948 | 7:150638484 | A | G | 0.244 | -0.0145 | 0.0019 | 1.95E-14 |
| Body mass index | rs56211164 | 7:158016764 | A | G | 0.2395 | -0.0128 | 0.0022 | 8.90E-09 |
| Body mass index | rs4721089 | 7:1872921 | T | C | 0.783 | 0.0167 | 0.0023 | 5.60E-13 |
| Body mass index | rs4307239 | 7:24354300 | A | G | 0.5361 | -0.0115 | 0.0017 | 1.47E-11 |
| Body mass index | rs213518 | 7:26941065 | T | C | 0.8498 | -0.0153 | 0.0024 | 2.49E-10 |
| Body mass index | rs215669 | 7:32378979 | A | G | 0.6024 | -0.0149 | 0.0017 | 8.94E-18 |
| Body mass index | rs2108719 | 7:39481056 | A | G | 0.727 | 0.0107 | 0.0019 | 2.28E-08 |
| Body mass index | rs217433 | 7:44553496 | T | C | 0.8031 | -0.0115 | 0.0021 | 3.00E-08 |
| Body mass index | rs2289379 | 7:44804225 | T | C | 0.3917 | -0.0137 | 0.0018 | 7.02E-15 |
| Body mass index | rs10499694 | 7:50614173 | A | G | 0.4935 | 0.013 | 0.0016 | 1.29E-15 |
| Body mass index | rs6463489 | 7:5542513 | T | C | 0.0959 | 0.0167 | 0.0026 | 2.50E-10 |
| Body mass index | rs11772246 | 7:71603692 | T | C | 0.8234 | 0.0145 | 0.0022 | 3.69E-11 |
| Body mass index | rs17207196 | 7:75101065 | T | C | 0.42 | -0.022 | 0.0017 | 1.58E-36 |
| Body mass index | rs17149254 | 7:76634463 | T | C | 0.2051 | 0.0238 | 0.0023 | 2.99E-25 |
| Body mass index | rs6973656 | 7:77422583 | A | G | 0.5836 | -0.0101 | 0.0017 | 6.31E-09 |
| Body mass index | rs1965529 | 7:77825707 | A | G | 0.773 | 0.0161 | 0.0022 | 7.66E-14 |
| Body mass index | rs274628 | 7:86265855 | A | C | 0.3366 | -0.0102 | 0.0018 | 1.36E-08 |
| Body mass index | rs2283006 | 7:93085722 | A | G | 0.4881 | 0.0132 | 0.0017 | 8.06E-15 |
| Body mass index | rs13240600 | 7:99064466 | A | G | 0.8343 | 0.018 | 0.0022 | 7.90E-16 |
| Body mass index | rs1383592 | 8:106430676 | A | G | 0.212 | 0.0122 | 0.0021 | 4.92E-09 |
| Body mass index | rs4240673 | 8:10787612 | T | C | 0.4545 | 0.0175 | 0.0016 | 1.58E-26 |
| Body mass index | rs3808477 | 8:116670347 | T | C | 0.274 | -0.0182 | 0.0019 | 8.73E-22 |
| Body mass index | rs72673947 | 8:118884379 | A | G | 0.8902 | -0.0223 | 0.0031 | 4.11E-13 |
| Body mass index | rs6470144 | 8:124152245 | T | G | 0.6503 | 0.0098 | 0.0018 | 3.06E-08 |
| Body mass index | rs7842934 | 8:132838921 | T | C | 0.9211 | -0.0178 | 0.0031 | 1.55E-08 |
| Body mass index | rs305256 | 8:137568252 | T | C | 0.2263 | -0.0115 | 0.0021 | 2.79E-08 |
| Body mass index | rs16906838 | 8:138213836 | T | C | 0.0486 | -0.0247 | 0.004 | 4.31E-10 |
| Body mass index | rs13263601 | 8:14095900 | A | C | 0.6585 | -0.0145 | 0.0018 | 4.87E-16 |
| Body mass index | rs11782074 | 8:142617096 | T | G | 0.3675 | 0.0124 | 0.0018 | 4.24E-12 |
| Body mass index | rs10099330 | 8:143383694 | A | G | 0.538 | -0.0119 | 0.0017 | 3.22E-12 |
| Body mass index | rs10110189 | 8:15393380 | T | C | 0.1057 | -0.0156 | 0.0028 | 2.20E-08 |
| Body mass index | rs10101364 | 8:20634888 | T | C | 0.6801 | 0.012 | 0.0018 | 5.61E-11 |
| Body mass index | rs73225274 | 8:21088909 | A | G | 0.8649 | -0.0156 | 0.0028 | 2.33E-08 |
| Body mass index | rs13249650 | 8:25664336 | A | T | 0.4443 | 0.011 | 0.0019 | 1.02E-08 |
| Body mass index | rs1421334 | 8:30865733 | A | C | 0.4546 | 0.0135 | 0.0017 | 3.11E-15 |
| Body mass index | rs2466103 | 8:32412304 | T | G | 0.6928 | -0.0121 | 0.0018 | 8.00E-12 |
| Body mass index | rs6468266 | 8:34380276 | A | T | 0.4176 | -0.0113 | 0.0017 | 7.71E-11 |
| Body mass index | rs1700082 | 8:4121727 | C | G | 0.659 | 0.0094 | 0.0017 | 3.38E-08 |
| Body mass index | rs12681792 | 8:62054463 | A | C | 0.2024 | 0.015 | 0.0021 | 2.88E-12 |
| Body mass index | rs12334877 | 8:67194171 | A | G | 0.1913 | -0.0144 | 0.0021 | 2.17E-11 |
| Body mass index | rs1808629 | 8:73435964 | A | G | 0.674 | -0.0202 | 0.002 | 2.19E-23 |
| Body mass index | rs17405819 | 8:76806584 | T | C | 0.6845 | 0.0211 | 0.0018 | 6.04E-33 |
| Body mass index | rs2196618 | 8:85089437 | A | G | 0.2634 | -0.0137 | 0.0019 | 1.49E-12 |
| Body mass index | rs12386885 | 8:87766769 | T | C | 0.1564 | 0.0151 | 0.0026 | 9.90E-09 |
| Body mass index | rs2120710 | 8:93210803 | A | G | 0.6529 | 0.0101 | 0.0018 | 1.98E-08 |
| Body mass index | rs12680842 | 8:95582606 | A | G | 0.6839 | 0.0142 | 0.0017 | 3.41E-16 |
| Body mass index | rs7025938 | 9:103088321 | C | G | 0.6798 | -0.0162 | 0.0018 | 1.47E-19 |
| Body mass index | rs7024334 | 9:109072075 | T | G | 0.2254 | 0.0135 | 0.002 | 4.71E-12 |
| Body mass index | rs1948080 | 9:11852043 | T | G | 0.6303 | 0.0136 | 0.0018 | 1.13E-14 |
| Body mass index | rs1928295 | 9:120378483 | T | C | 0.5712 | 0.0134 | 0.0016 | 2.23E-16 |
| Body mass index | rs1877875 | 9:120664469 | T | C | 0.4341 | -0.0109 | 0.0017 | 2.51E-10 |
| Body mass index | rs13298487 | 9:126112104 | T | C | 0.6076 | 0.0115 | 0.002 | 1.43E-08 |
| Body mass index | rs10733682 | 9:129460914 | A | G | 0.4594 | 0.0148 | 0.0016 | 1.76E-19 |
| Body mass index | rs2267958 | 9:131015279 | A | G | 0.5088 | -0.013 | 0.0018 | 2.01E-13 |
| Body mass index | rs4740383 | 9:133783566 | A | G | 0.4185 | 0.0131 | 0.0018 | 7.03E-14 |
| Body mass index | rs10858334 | 9:137989785 | C | G | 0.8484 | -0.0148 | 0.0025 | 4.91E-09 |
| Body mass index | rs10961649 | 9:14670949 | T | C | 0.3238 | 0.0104 | 0.0018 | 1.51E-08 |
| Body mass index | rs4740619 | 9:15634326 | T | C | 0.5436 | 0.0189 | 0.0016 | 3.15E-31 |
| Body mass index | rs10962550 | 9:16720329 | C | G | 0.1756 | 0.0186 | 0.0022 | 5.68E-17 |
| Body mass index | rs10811868 | 9:23199959 | A | G | 0.32 | -0.01 | 0.0018 | 3.18E-08 |
| Body mass index | rs10968114 | 9:27800007 | A | C | 0.5331 | 0.0113 | 0.0017 | 3.30E-11 |
| Body mass index | rs1412235 | 9:28410996 | C | G | 0.3151 | 0.0237 | 0.0017 | 2.28E-42 |
| Body mass index | rs10121187 | 9:29737191 | C | G | 0.509 | 0.0102 | 0.0017 | 2.02E-09 |
| Body mass index | rs2275003 | 9:34124860 | A | G | 0.5007 | 0.0111 | 0.0016 | 9.44E-12 |
| Body mass index | rs13296413 | 9:37258105 | T | C | 0.3811 | -0.0148 | 0.0017 | 2.49E-17 |
| Body mass index | rs10975933 | 9:6954557 | C | G | 0.6634 | 0.0114 | 0.0018 | 2.07E-10 |
| Body mass index | rs2134858 | 9:73837155 | T | C | 0.5119 | -0.0117 | 0.0017 | 5.87E-12 |
| Body mass index | rs7861160 | 9:80799579 | T | C | 0.5941 | 0.0095 | 0.0017 | 3.68E-08 |
| Body mass index | rs1999433 | 9:81371441 | T | C | 0.4403 | -0.0107 | 0.0017 | 2.90E-10 |
| Body mass index | rs2777768 | 9:84186734 | A | G | 0.7228 | 0.0119 | 0.0019 | 6.38E-10 |
| Body mass index | rs7357754 | 9:92207308 | A | G | 0.4985 | -0.012 | 0.0017 | 1.81E-12 |
| Body mass index | rs10992867 | 9:96461013 | A | G | 0.2687 | 0.0162 | 0.0019 | 3.20E-17 |
| Carbohydrate | rs10206338 | 2:60209981 | A | G | 0.578 | -0.01577 | 0.002787 | 1.52E-08 |
| Carbohydrate | rs10510554 | 3:25099776 | T | C | 0.428 | 0.019417 | 0.002781 | 2.94E-12 |
| Carbohydrate | rs10433500 | 3:85546798 | A | G | 0.6371 | 0.016076 | 0.002863 | 1.96E-08 |
| Carbohydrate | rs7012637 | 8:9173209 | A | G | 0.4728 | 0.017343 | 0.002784 | 4.68E-10 |
| Carbohydrate | rs10962121 | 9:15702704 | T | G | 0.4989 | -0.01519 | 0.002752 | 3.40E-08 |
| Carbohydrate | rs2472297 | 15:75027880 | T | C | 0.2603 | -0.01795 | 0.003262 | 3.73E-08 |
| Carbohydrate | rs7190396 | 16:53822502 | T | G | 0.6033 | 0.017812 | 0.002812 | 2.39E-10 |
| Carbohydrate | rs1104608 | 16:73912588 | C | G | 0.4231 | 0.018467 | 0.002893 | 1.74E-10 |
| Carbohydrate | rs36123991 | 17:44359663 | T | G | 0.2055 | 0.021297 | 0.003695 | 8.24E-09 |
| Carbohydrate | rs8097672 | 18:1839601 | A | T | 0.8549 | 0.023456 | 0.003908 | 1.95E-09 |
| Carbohydrate | rs838144 | 19:49250239 | T | C | 0.5089 | -0.02323 | 0.002753 | 3.26E-17 |
| Carbohydrate | rs9987289 | 8:9183358 | A | T | 0.0903 | -0.02623 | 0.0048 | 4.64E-08 |
| Carbohydrate | rs429358 | 19:45411941 | T | C | 0.8483 | -0.02698 | 0.003879 | 3.49E-12 |
| Fat | rs1229984 | 4:100239319 | T | C | 0.0293 | 0.09777 | 0.008862 | 2.64E-28 |
| Fat | rs57193069 | 7:1862417 | A | G | 0.5456 | -0.01574 | 0.002796 | 1.80E-08 |
| Fat | rs7012814 | 8:9173358 | A | G | 0.4727 | -0.0189 | 0.002783 | 1.12E-11 |
| Fat | rs33988101 | 19:49218111 | T | G | 0.5298 | -0.0294 | 0.002759 | 1.66E-26 |
| Fat | rs9927317 | 16:53820996 | C | G | 0.6069 | -0.0242 | 0.003501 | 4.77E-12 |
| Fat | rs429358 | 19:45411941 | T | G | 0.8483 | 0.023788 | 0.003879 | 8.65E-10 |
| Protein | rs780094 | 2:27741237 | T | C | 0.3871 | 0.017517 | 0.002824 | 5.58E-10 |
| Protein | rs445551 | 2:79697982 | A | G | 0.3072 | 0.019189 | 0.003389 | 1.49E-08 |
| Protein | rs1603978 | 3:25108236 | A | C | 0.6935 | 0.019168 | 0.002985 | 1.35E-10 |
| Protein | rs13146907 | 4:39425248 | A | G | 0.6214 | -0.0219 | 0.002839 | 1.24E-14 |
| Protein | rs1461729 | 8:9187242 | A | G | 0.0998 | 0.03183 | 0.00459 | 4.09E-12 |
| Protein | rs55872725 | 16:53809123 | T | C | 0.4072 | 0.017793 | 0.0028 | 2.09E-10 |
| Protein | rs838133 | 19:49259529 | A | G | 0.4469 | -0.03182 | 0.003013 | 4.52E-26 |
| Sugar | rs12713415 | 2:60205134 | C | G | 0.71 | -0.019 | 0.003 | 4.88E-09 |
| Sugar | rs7619139 | 3:25110415 | A | T | 0.59 | -0.024 | 0.003 | 4.98E-16 |
| Sugar | rs13202107 | 6:51395463 | A | G | 0.217 | -0.02 | 0.004 | 1.77E-08 |
| Sugar | rs7012814 | 8:9173358 | A | G | 0.472 | 0.019 | 0.003 | 4.99E-10 |
| Sugar | rs9972653 | 16:53814363 | T | G | 0.399 | -0.02 | 0.003 | 1.53E-11 |
| Sugar | rs8097672 | 18:1839601 | A | T | 0.854 | 0.03 | 0.004 | 1.54E-12 |
| Sugar | rs341228 | 18:6395336 | T | C | 0.331 | 0.019 | 0.003 | 2.72E-09 |
| Sugar | rs838144 | 19:49250239 | T | C | 0.508 | -0.028 | 0.003 | 8.53E-21 |
| Sugar | rs62132802 | 19:49270872 | T | C | 0.308 | -0.02017 | 0.003527 | 1.07E-08 |
| Sugar | rs429358 | 19:45411941 | T | C | 0.8488 | -0.02769 | 0.004164 | 2.97E-11 |
| Smoking per day | rs2118359 | 11:16231392 | T | G | 0.229 | 0.026 | 0.005 | 1.03E-08 |
| Smoking per day | rs78385743 | 15:78750388 | T | A | 0.034 | -0.062 | 0.01 | 1.36E-10 |
| Smoking per day | rs12442456 | 15:78751962 | T | G | 0.148 | -0.06 | 0.006 | 2.45E-27 |
| Smoking per day | rs114642875 | 15:78763289 | A | G | 0.015 | 0.083 | 0.014 | 6.61E-09 |
| Smoking per day | rs9788721 | 15:78802869 | T | C | 0.652 | -0.09 | 0.004 | ###### |
| Smoking per day | rs147760547 | 15:78816130 | G | A | 0.022 | 0.079 | 0.012 | 2.22E-10 |
| Smoking per day | rs189302121 | 15:78892421 | T | A | 0.028 | 0.06 | 0.01 | 7.53E-09 |
| Smoking per day | rs112272197 | 15:78892513 | T | C | 0.04 | 0.064 | 0.01 | 3.69E-11 |
| Smoking per day | rs3813567 | 15:78934551 | A | G | 0.779 | 0.058 | 0.005 | 2.58E-35 |
| Smoking per day | rs117189732 | 15:79083280 | T | C | 0.083 | 0.047 | 0.007 | 2.35E-12 |
| Smoking per day | rs8034274 | 15:79126155 | C | T | 0.565 | -0.041 | 0.004 | 1.40E-26 |
| Smoking per day | rs79409323 | 15:79164636 | C | T | 0.021 | -0.06 | 0.011 | 4.56E-08 |
| Smoking per day | rs1579233 | 16:52074530 | G | A | 0.571 | -0.023 | 0.004 | 1.94E-09 |
| Smoking per day | rs56113850 | 19:41353107 | C | T | 0.568 | 0.04 | 0.004 | 8.58E-26 |
| Smoking per day | rs28399442 | 19:41354458 | A | C | 0.02 | -0.098 | 0.011 | 4.22E-18 |
| Smoking per day | rs1801272 | 19:41354533 | T | A | 0.023 | -0.085 | 0.013 | 3.24E-11 |
| Smoking per day | rs59586387 | 19:41375030 | G | C | 0.072 | -0.039 | 0.007 | 2.27E-08 |
| Smoking per day | rs2273500 | 20:61986949 | C | T | 0.159 | 0.034 | 0.005 | 8.58E-11 |
| Smoking per day | rs215600 | 7:32333642 | A | G | 0.64 | -0.025 | 0.004 | 2.06E-10 |
| Smoking per day | rs58379124 | 8:42579203 | C | T | 0.748 | 0.034 | 0.004 | 2.06E-14 |
| Smoking per day | rs3025383 | 9:136502369 | C | T | 0.18 | -0.032 | 0.005 | 1.19E-11 |
| Lifetime smoking index | rs9435340 | 1:107593201 | T | A | 0.344 | 0.008 | 0.001 | 1.20E-08 |
| Lifetime smoking index | rs10918701 | 1:162090536 | G | A | 0.372 | 0.008 | 0.001 | 2.10E-08 |
| Lifetime smoking index | rs4949465 | 1:32178489 | T | C | 0.87 | -0.012 | 0.002 | 1.70E-08 |
| Lifetime smoking index | rs549845 | 1:44076469 | G | A | 0.301 | 0.011 | 0.002 | 8.30E-14 |
| Lifetime smoking index | rs1933270 | 1:49977965 | T | G | 0.364 | 0.009 | 0.001 | 1.50E-10 |
| Lifetime smoking index | rs7528604 | 1:66407352 | G | A | 0.566 | 0.01 | 0.001 | 5.70E-12 |
| Lifetime smoking index | rs11210229 | 1:73860028 | A | G | 0.384 | 0.012 | 0.001 | 2.00E-16 |
| Lifetime smoking index | rs7553348 | 1:75005067 | G | A | 0.438 | 0.01 | 0.001 | 5.20E-12 |
| Lifetime smoking index | rs1193237 | 1:7526486 | G | C | 0.439 | -0.008 | 0.001 | 2.80E-08 |
| Lifetime smoking index | rs10922907 | 1:91193049 | A | T | 0.451 | 0.01 | 0.001 | 3.00E-13 |
| Lifetime smoking index | rs1931263 | 1:96175101 | G | T | 0.51 | -0.008 | 0.001 | 4.00E-08 |
| Lifetime smoking index | rs7519626 | 1:99514554 | C | T | 0.324 | 0.008 | 0.001 | 1.20E-08 |
| Lifetime smoking index | rs7077678 | 10:104438565 | C | T | 0.623 | 0.009 | 0.001 | 2.60E-09 |
| Lifetime smoking index | rs12244388 | 10:104640052 | G | A | 0.661 | -0.013 | 0.001 | 1.40E-19 |
| Lifetime smoking index | rs3896224 | 10:106467853 | A | G | 0.585 | 0.01 | 0.001 | 1.10E-11 |
| Lifetime smoking index | rs2675638 | 10:63576286 | G | A | 0.581 | 0.008 | 0.001 | 1.30E-09 |
| Lifetime smoking index | rs10823968 | 10:74738269 | A | T | 0.633 | 0.008 | 0.001 | 2.10E-08 |
| Lifetime smoking index | rs11255908 | 10:8802912 | T | G | 0.743 | -0.01 | 0.002 | 2.30E-10 |
| Lifetime smoking index | rs17553262 | 10:92912773 | A | C | 0.885 | -0.013 | 0.002 | 5.30E-09 |
| Lifetime smoking index | rs9919670 | 11:112877304 | G | A | 0.612 | -0.015 | 0.001 | 7.60E-27 |
| Lifetime smoking index | rs34866095 | 11:16377356 | A | G | 0.686 | -0.009 | 0.002 | 1.20E-08 |
| Lifetime smoking index | rs75742406 | 11:17070365 | G | A | 0.739 | 0.01 | 0.002 | 1.30E-09 |
| Lifetime smoking index | rs17309874 | 11:27667236 | G | A | 0.74 | -0.011 | 0.002 | 9.70E-13 |
| Lifetime smoking index | rs4391802 | 11:28674592 | A | G | 0.707 | 0.01 | 0.002 | 1.40E-11 |
| Lifetime smoking index | rs112282219 | 11:46632809 | G | A | 0.959 | -0.023 | 0.004 | 3.80E-11 |
| Lifetime smoking index | rs74086911 | 12:50015942 | G | A | 0.925 | 0.015 | 0.003 | 2.10E-08 |
| Lifetime smoking index | rs7297175 | 12:56473808 | T | C | 0.431 | -0.008 | 0.001 | 6.60E-09 |
| Lifetime smoking index | rs10879871 | 12:75380511 | T | G | 0.343 | -0.01 | 0.001 | 5.00E-11 |
| Lifetime smoking index | rs12831617 | 12:84758368 | C | T | 0.764 | -0.009 | 0.002 | 1.90E-08 |
| Lifetime smoking index | rs7333559 | 13:100546450 | G | A | 0.212 | 0.011 | 0.002 | 3.20E-10 |
| Lifetime smoking index | rs6562474 | 13:67332812 | C | G | 0.651 | 0.008 | 0.001 | 1.00E-08 |
| Lifetime smoking index | rs3742365 | 14:104198251 | T | C | 0.595 | -0.011 | 0.001 | 2.50E-14 |
| Lifetime smoking index | rs860326 | 14:57342912 | C | T | 0.428 | 0.008 | 0.001 | 2.70E-09 |
| Lifetime smoking index | rs7155595 | 14:77502546 | A | C | 0.674 | -0.009 | 0.001 | 2.50E-09 |
| Lifetime smoking index | rs35175834 | 15:47680815 | G | A | 0.788 | -0.016 | 0.002 | 4.60E-22 |
| Lifetime smoking index | rs28485305 | 15:74044197 | C | T | 0.631 | 0.008 | 0.001 | 2.60E-08 |
| Lifetime smoking index | rs8042849 | 15:78817929 | C | T | 0.342 | 0.019 | 0.001 | 1.80E-39 |
| Lifetime smoking index | rs8042134 | 15:97514404 | T | G | 0.541 | -0.01 | 0.001 | 1.30E-12 |
| Lifetime smoking index | rs6598539 | 15:99204483 | T | C | 0.489 | -0.008 | 0.001 | 4.50E-09 |
| Lifetime smoking index | rs12708665 | 16:24728227 | A | G | 0.285 | -0.009 | 0.002 | 3.50E-09 |
| Lifetime smoking index | rs57611503 | 16:31165795 | G | A | 0.485 | 0.008 | 0.001 | 4.00E-08 |
| Lifetime smoking index | rs889398 | 16:69556715 | C | T | 0.588 | 0.009 | 0.001 | 6.30E-11 |
| Lifetime smoking index | rs11861214 | 16:746611 | G | T | 0.784 | 0.009 | 0.002 | 2.00E-08 |
| Lifetime smoking index | rs60952428 | 16:75640521 | T | C | 0.909 | 0.013 | 0.002 | 3.00E-08 |
| Lifetime smoking index | rs1050847 | 16:87443734 | C | T | 0.426 | 0.008 | 0.001 | 1.40E-08 |
| Lifetime smoking index | rs369230 | 16:89645437 | G | T | 0.308 | -0.009 | 0.002 | 1.80E-09 |
| Lifetime smoking index | rs8614 | 17:27588806 | C | A | 0.817 | -0.011 | 0.002 | 1.80E-10 |
| Lifetime smoking index | rs732083 | 17:37834367 | G | A | 0.333 | 0.008 | 0.001 | 1.50E-08 |
| Lifetime smoking index | rs9904288 | 17:47031973 | T | C | 0.708 | 0.008 | 0.002 | 3.10E-08 |
| Lifetime smoking index | rs67596067 | 17:50333733 | G | A | 0.649 | -0.009 | 0.001 | 1.20E-09 |
| Lifetime smoking index | rs12967855 | 18:35138245 | A | G | 0.331 | 0.008 | 0.001 | 3.10E-08 |
| Lifetime smoking index | rs62098013 | 18:50863861 | G | A | 0.64 | -0.009 | 0.001 | 4.10E-09 |
| Lifetime smoking index | rs71367545 | 18:77576337 | G | A | 0.791 | -0.01 | 0.002 | 1.40E-09 |
| Lifetime smoking index | rs35343344 | 19:18471610 | C | A | 0.733 | 0.009 | 0.002 | 8.80E-09 |
| Lifetime smoking index | rs76608582 | 19:4474725 | C | A | 0.953 | 0.022 | 0.003 | 3.20E-10 |
| Lifetime smoking index | rs2678670 | 2:104469564 | A | T | 0.486 | 0.009 | 0.001 | 3.10E-10 |
| Lifetime smoking index | rs62155874 | 2:105973094 | A | G | 0.873 | -0.017 | 0.002 | 5.20E-16 |
| Lifetime smoking index | rs3811038 | 2:113240183 | T | C | 0.724 | -0.01 | 0.002 | 8.90E-10 |
| Lifetime smoking index | rs2890772 | 2:146175106 | G | T | 0.413 | -0.014 | 0.001 | 2.10E-22 |
| Lifetime smoking index | rs62175972 | 2:161362830 | T | C | 0.966 | 0.022 | 0.004 | 1.70E-08 |
| Lifetime smoking index | rs3769949 | 2:166199284 | T | A | 0.528 | -0.008 | 0.001 | 2.50E-09 |
| Lifetime smoking index | rs13009008 | 2:174043233 | A | G | 0.328 | 0.009 | 0.001 | 4.60E-09 |
| Lifetime smoking index | rs4473348 | 2:182073742 | A | T | 0.25 | -0.01 | 0.002 | 6.40E-11 |
| Lifetime smoking index | rs12623702 | 2:202885506 | A | G | 0.613 | -0.01 | 0.001 | 7.70E-12 |
| Lifetime smoking index | rs6741228 | 2:22548774 | T | C | 0.433 | 0.008 | 0.001 | 1.60E-08 |
| Lifetime smoking index | rs62135536 | 2:44326028 | C | T | 0.968 | 0.024 | 0.004 | 8.00E-10 |
| Lifetime smoking index | rs7569203 | 2:45154418 | A | C | 0.689 | -0.011 | 0.002 | 7.40E-13 |
| Lifetime smoking index | rs13016665 | 2:57995348 | C | A | 0.577 | -0.008 | 0.001 | 1.80E-09 |
| Lifetime smoking index | rs4671357 | 2:60136176 | T | C | 0.519 | -0.009 | 0.001 | 1.10E-11 |
| Lifetime smoking index | rs359243 | 2:60475509 | T | C | 0.393 | -0.009 | 0.001 | 9.50E-10 |
| Lifetime smoking index | rs2867112 | 2:651349 | T | G | 0.835 | 0.015 | 0.002 | 4.80E-15 |
| Lifetime smoking index | rs4814873 | 20:19616429 | C | T | 0.767 | 0.01 | 0.002 | 2.90E-09 |
| Lifetime smoking index | rs6119897 | 20:31145415 | G | A | 0.762 | -0.013 | 0.002 | 3.60E-15 |
| Lifetime smoking index | rs12481282 | 20:44761377 | G | C | 0.722 | -0.009 | 0.002 | 7.80E-09 |
| Lifetime smoking index | rs348809 | 20:59032097 | A | G | 0.348 | -0.008 | 0.001 | 1.30E-08 |
| Lifetime smoking index | rs6011779 | 20:61984317 | C | T | 0.191 | 0.019 | 0.002 | 2.30E-27 |
| Lifetime smoking index | rs147412694 | 21:40702786 | G | A | 0.85 | -0.012 | 0.002 | 2.90E-09 |
| Lifetime smoking index | rs2838834 | 21:46665208 | C | T | 0.699 | -0.009 | 0.002 | 6.30E-10 |
| Lifetime smoking index | rs136233 | 22:31212410 | A | G | 0.809 | -0.01 | 0.002 | 1.80E-08 |
| Lifetime smoking index | rs202645 | 22:41798520 | A | G | 0.203 | -0.01 | 0.002 | 3.90E-09 |
| Lifetime smoking index | rs326341 | 3:107811142 | G | A | 0.525 | 0.009 | 0.001 | 1.20E-11 |
| Lifetime smoking index | rs73220544 | 3:131074511 | A | C | 0.842 | -0.011 | 0.002 | 1.50E-08 |
| Lifetime smoking index | rs9842947 | 3:157412246 | C | T | 0.326 | -0.009 | 0.001 | 3.10E-09 |
| Lifetime smoking index | rs6779302 | 3:16859710 | G | T | 0.633 | -0.009 | 0.001 | 1.20E-09 |
| Lifetime smoking index | rs6778080 | 3:49317338 | T | C | 0.267 | 0.011 | 0.002 | 1.30E-12 |
| Lifetime smoking index | rs775758 | 3:77582005 | A | T | 0.433 | 0.008 | 0.001 | 1.10E-08 |
| Lifetime smoking index | rs421983 | 3:84892866 | T | C | 0.519 | 0.009 | 0.001 | 3.30E-10 |
| Lifetime smoking index | rs72678864 | 4:112422145 | G | A | 0.829 | 0.012 | 0.002 | 1.60E-11 |
| Lifetime smoking index | rs17576594 | 4:147952241 | G | A | 0.724 | 0.011 | 0.002 | 1.70E-12 |
| Lifetime smoking index | rs61796681 | 4:23678196 | A | T | 0.912 | -0.013 | 0.002 | 4.20E-08 |
| Lifetime smoking index | rs624833 | 4:2881256 | T | G | 0.695 | 0.009 | 0.002 | 6.60E-10 |
| Lifetime smoking index | rs317021 | 4:35418368 | T | A | 0.814 | -0.012 | 0.002 | 1.10E-10 |
| Lifetime smoking index | rs4957528 | 5:106420589 | A | C | 0.208 | -0.01 | 0.002 | 4.20E-09 |
| Lifetime smoking index | rs11948770 | 5:13246336 | T | C | 0.768 | -0.01 | 0.002 | 4.90E-10 |
| Lifetime smoking index | rs329120 | 5:133861756 | C | T | 0.581 | 0.01 | 0.001 | 6.30E-12 |
| Lifetime smoking index | rs986391 | 5:166993972 | G | A | 0.367 | 0.011 | 0.001 | 9.40E-15 |
| Lifetime smoking index | rs13153393 | 5:167604213 | A | G | 0.884 | -0.014 | 0.002 | 2.50E-10 |
| Lifetime smoking index | rs245774 | 5:170530930 | A | G | 0.272 | -0.009 | 0.002 | 7.40E-09 |
| Lifetime smoking index | rs71627581 | 5:43161351 | G | A | 0.889 | 0.013 | 0.002 | 1.60E-09 |
| Lifetime smoking index | rs10052591 | 5:50812738 | T | C | 0.573 | 0.008 | 0.001 | 2.10E-09 |
| Lifetime smoking index | rs2080870 | 5:60388313 | A | T | 0.258 | 0.009 | 0.002 | 4.90E-08 |
| Lifetime smoking index | rs4571506 | 5:87756918 | C | T | 0.54 | 0.008 | 0.001 | 1.50E-08 |
| Lifetime smoking index | rs7766610 | 6:111707821 | C | A | 0.183 | 0.013 | 0.002 | 2.20E-12 |
| Lifetime smoking index | rs6935954 | 6:26255451 | A | G | 0.421 | 0.01 | 0.001 | 8.20E-12 |
| Lifetime smoking index | rs2254710 | 6:37477000 | C | A | 0.236 | 0.009 | 0.002 | 3.50E-08 |
| Lifetime smoking index | rs2894808 | 6:52861990 | T | A | 0.922 | -0.015 | 0.003 | 3.50E-09 |
| Lifetime smoking index | rs12202536 | 6:67475273 | A | G | 0.513 | -0.008 | 0.001 | 2.80E-09 |
| Lifetime smoking index | rs10282292 | 7:111092478 | C | T | 0.362 | 0.009 | 0.001 | 5.90E-10 |
| Lifetime smoking index | rs2401924 | 7:115057862 | G | C | 0.502 | 0.011 | 0.001 | 2.70E-14 |
| Lifetime smoking index | rs7807019 | 7:117543063 | A | G | 0.54 | -0.01 | 0.001 | 6.70E-14 |
| Lifetime smoking index | rs6957896 | 7:132309592 | C | T | 0.503 | -0.008 | 0.001 | 4.50E-08 |
| Lifetime smoking index | rs4731925 | 7:132664757 | C | T | 0.316 | -0.008 | 0.001 | 2.60E-08 |
| Lifetime smoking index | rs10226228 | 7:32315613 | A | G | 0.63 | -0.011 | 0.001 | 2.00E-15 |
| Lifetime smoking index | rs1922018 | 7:3560401 | C | T | 0.364 | 0.01 | 0.001 | 3.00E-12 |
| Lifetime smoking index | rs11768481 | 7:96629103 | C | A | 0.666 | 0.009 | 0.001 | 9.90E-10 |
| Lifetime smoking index | rs6962772 | 7:99081730 | A | G | 0.846 | 0.011 | 0.002 | 7.80E-09 |
| Lifetime smoking index | rs11783093 | 8:27425349 | C | T | 0.839 | 0.016 | 0.002 | 1.20E-16 |
| Lifetime smoking index | rs2062882 | 8:91839576 | G | A | 0.587 | -0.008 | 0.001 | 1.10E-08 |
| Lifetime smoking index | rs72674867 | 8:95578201 | A | T | 0.765 | 0.009 | 0.002 | 3.80E-08 |
| Lifetime smoking index | rs35169606 | 8:9604066 | T | G | 0.612 | 0.009 | 0.001 | 1.20E-09 |
| Lifetime smoking index | rs1221148 | 9:122046875 | C | G | 0.587 | 0.009 | 0.001 | 7.30E-11 |
| Lifetime smoking index | rs13296519 | 9:128471924 | G | T | 0.606 | -0.01 | 0.001 | 8.10E-12 |
| Lifetime smoking index | rs113382419 | 9:136463019 | C | A | 0.889 | -0.028 | 0.002 | 3.00E-37 |
| Lifetime smoking index | rs4543592 | 9:3014254 | T | C | 0.52 | -0.009 | 0.001 | 4.50E-10 |
| Lifetime smoking index | rs7039819 | 9:82430418 | G | A | 0.427 | 0.009 | 0.001 | 5.10E-10 |
| Lifetime smoking index | rs1246265 | 9:86761745 | T | C | 0.305 | -0.009 | 0.002 | 4.20E-09 |
| Drinks per week | rs600348 | 1:107998734 | T | C | 0.405 | -0.014 | 0.003 | 3.63E-06 |
| Drinks per week | rs58324444 | 1:221192226 | T | C | 0.018 | -0.068 | 0.015 | 3.20E-06 |
| Drinks per week | rs3827886 | 12:14660184 | C | T | 0.126 | -0.02 | 0.004 | 2.64E-06 |
| Drinks per week | rs1027172 | 12:92169415 | T | A | 0.6 | -0.015 | 0.003 | 1.74E-06 |
| Drinks per week | rs7193413 | 16:28644663 | T | A | 0.693 | -0.015 | 0.003 | 1.60E-06 |
| Drinks per week | rs7187575 | 16:28990101 | T | C | 0.703 | 0.019 | 0.003 | 5.23E-09 |
| Drinks per week | rs13332432 | 16:85721809 | G | C | 0.296 | 0.017 | 0.003 | 6.09E-07 |
| Drinks per week | rs676388 | 19:49211969 | C | T | 0.494 | 0.018 | 0.003 | 2.31E-09 |
| Drinks per week | rs72857537 | 2:147830318 | C | T | 0.039 | 0.038 | 0.008 | 2.21E-06 |
| Drinks per week | rs57595195 | 2:15325152 | A | T | 0.1 | 0.028 | 0.006 | 6.91E-07 |
| Drinks per week | rs4667699 | 2:164539577 | A | G | 0.104 | 0.025 | 0.005 | 2.02E-07 |
| Drinks per week | rs16849203 | 2:164889473 | C | T | 0.027 | 0.041 | 0.009 | 1.22E-06 |
| Drinks per week | rs112621228 | 2:202748440 | T | C | 0.035 | 0.045 | 0.009 | 1.01E-07 |
| Drinks per week | rs1260326 | 2:27730940 | C | T | 0.595 | 0.014 | 0.003 | 4.74E-06 |
| Drinks per week | rs13032049 | 2:63581507 | G | A | 0.28 | 0.016 | 0.003 | 1.43E-06 |
| Drinks per week | rs9880277 | 3:13348742 | G | C | 0.114 | 0.021 | 0.005 | 3.38E-06 |
| Drinks per week | rs2247173 | 3:158468442 | G | A | 0.98 | -0.054 | 0.011 | 6.23E-07 |
| Drinks per week | rs66490435 | 3:2045084 | T | C | 0.173 | -0.018 | 0.004 | 2.83E-06 |
| Drinks per week | rs62253088 | 3:85400801 | C | T | 0.663 | -0.015 | 0.003 | 1.30E-06 |
| Drinks per week | rs1229984 | 4:100239319 | C | T | 0.953 | 0.145 | 0.008 | 1.12E-65 |
| Drinks per week | rs1229978 | 4:100256199 | C | T | 0.398 | 0.021 | 0.003 | 3.04E-12 |
| Drinks per week | rs283412 | 4:100267672 | G | A | 0.972 | 0.05 | 0.01 | 2.74E-07 |
| Drinks per week | rs35225200 | 4:103146888 | C | A | 0.071 | -0.034 | 0.006 | 8.08E-08 |
| Drinks per week | rs4696055 | 4:174217122 | G | A | 0.198 | 0.017 | 0.004 | 2.79E-06 |
| Drinks per week | rs111615986 | 4:18040190 | T | C | 0.03 | -0.045 | 0.009 | 3.75E-07 |
| Drinks per week | rs140330848 | 4:188485679 | T | G | 0.023 | -0.05 | 0.011 | 2.27E-06 |
| Drinks per week | rs7441955 | 4:32860650 | T | C | 0.011 | -0.069 | 0.015 | 2.27E-06 |
| Drinks per week | rs11940694 | 4:39414993 | G | A | 0.595 | 0.023 | 0.003 | 4.82E-14 |
| Drinks per week | rs2687963 | 4:39438149 | A | G | 0.707 | -0.017 | 0.003 | 3.42E-07 |
| Drinks per week | rs1795722 | 4:89942863 | T | C | 0.808 | 0.017 | 0.004 | 1.24E-06 |
| Drinks per week | rs71612659 | 4:99691047 | A | G | 0.06 | -0.035 | 0.006 | 1.26E-08 |
| Drinks per week | rs4699680 | 4:99759132 | A | G | 0.951 | 0.042 | 0.007 | 1.97E-08 |
| Drinks per week | rs112609127 | 5:126377570 | A | C | 0.039 | -0.038 | 0.008 | 4.22E-06 |
| Drinks per week | rs55872084 | 5:155902003 | T | G | 0.218 | 0.02 | 0.004 | 2.20E-08 |
| Drinks per week | rs56387435 | 6:134937080 | C | A | 0.12 | 0.021 | 0.005 | 2.63E-06 |
| Drinks per week | rs9504848 | 6:6566290 | C | T | 0.464 | 0.014 | 0.003 | 4.10E-06 |
| Drinks per week | rs1558413 | 7:69504764 | G | C | 0.329 | -0.015 | 0.003 | 1.40E-06 |
| Drinks per week | rs6987656 | 8:93264306 | A | G | 0.764 | 0.016 | 0.003 | 4.63E-06 |
| Drinks per week | rs10964229 | 9:19583437 | T | C | 0.139 | 0.02 | 0.004 | 4.18E-06 |
| Sedentary behavior | rs4847408 | 1:93791437 | C | G | 0.649373 | -0.0104 | 0.001663 | 4.10E-10 |
| Sedentary behavior | rs7539775 | 1:3109151 | A | G | 0.743419 | 0.009969 | 0.00182 | 4.30E-08 |
| Sedentary behavior | rs1085727 | 1:84706933 | A | T | 0.69299 | 0.009544 | 0.001733 | 3.60E-08 |
| Sedentary behavior | rs4469687 | 1:184679019 | G | A | 0.483936 | 0.008799 | 0.00159 | 3.10E-08 |
| Sedentary behavior | rs7414210 | 1:97812612 | C | A | 0.161463 | 0.012754 | 0.002173 | 4.40E-09 |
| Sedentary behavior | rs12045585 | 1:243673099 | A | G | 0.131234 | -0.01506 | 0.00241 | 4.10E-10 |
| Sedentary behavior | rs1324491 | 1:60350616 | A | G | 0.130982 | 0.013413 | 0.002357 | 1.30E-08 |
| Sedentary behavior | rs71658797 | 1:77967507 | A | T | 0.121135 | 0.015758 | 0.00244 | 1.10E-10 |
| Sedentary behavior | rs749056 | 1:110037838 | G | T | 0.304306 | -0.01002 | 0.001732 | 7.20E-09 |
| Sedentary behavior | rs72768080 | 1:241871634 | C | T | 0.209946 | -0.0119 | 0.001985 | 2.10E-09 |
| Sedentary behavior | rs75641275 | 1:98327133 | C | A | 0.14333 | 0.015433 | 0.00227 | 1.00E-11 |
| Sedentary behavior | rs1730858 | 1:107619244 | C | T | 0.651431 | -0.00935 | 0.001685 | 2.90E-08 |
| Sedentary behavior | rs814197 | 1:61092456 | G | T | 0.466816 | -0.01068 | 0.001594 | 2.00E-11 |
| Sedentary behavior | rs883027 | 2:50600165 | C | G | 0.419695 | 0.009679 | 0.001613 | 2.00E-09 |
| Sedentary behavior | rs62145951 | 2:68399586 | C | T | 0.263053 | -0.01203 | 0.001804 | 2.60E-11 |
| Sedentary behavior | rs872169 | 2:24259188 | G | C | 0.205676 | 0.011346 | 0.001968 | 8.20E-09 |
| Sedentary behavior | rs4675246 | 2:202864487 | T | G | 0.200277 | 0.011862 | 0.001981 | 2.10E-09 |
| Sedentary behavior | rs3754970 | 2:162091836 | C | T | 0.502922 | 0.009702 | 0.001599 | 1.30E-09 |
| Sedentary behavior | rs11680095 | 2:181825956 | T | C | 0.592713 | -0.00919 | 0.001634 | 1.90E-08 |
| Sedentary behavior | rs10189857 | 2:60713235 | G | A | 0.432324 | 0.014686 | 0.001605 | 5.70E-20 |
| Sedentary behavior | rs6740081 | 2:116323703 | C | T | 0.71078 | 0.010512 | 0.001755 | 2.10E-09 |
| Sedentary behavior | rs13014947 | 2:193742999 | A | G | 0.574722 | 0.010607 | 0.001624 | 6.50E-11 |
| Sedentary behavior | rs2678662 | 2:104446759 | G | T | 0.608337 | 0.011146 | 0.001632 | 8.60E-12 |
| Sedentary behavior | rs6742621 | 2:107561110 | A | T | 0.576309 | -0.01085 | 0.001612 | 1.70E-11 |
| Sedentary behavior | rs1451533 | 2:105466005 | A | G | 0.273764 | 0.009829 | 0.001796 | 4.40E-08 |
| Sedentary behavior | rs68056254 | 2:147846855 | T | G | 0.150874 | 0.013495 | 0.002225 | 1.30E-09 |
| Sedentary behavior | rs73946726 | 2:117073427 | A | C | 0.02024 | 0.031959 | 0.005708 | 2.20E-08 |
| Sedentary behavior | rs4303732 | 2:100830040 | C | T | 0.40162 | -0.01073 | 0.001621 | 3.70E-11 |
| Sedentary behavior | rs62199883 | 2:215376706 | A | C | 0.48612 | 0.013843 | 0.00159 | 3.20E-18 |
| Sedentary behavior | rs263771 | 2:185921692 | A | C | 0.233452 | 0.012014 | 0.001885 | 1.90E-10 |
| Sedentary behavior | rs34109383 | 2:191464451 | T | C | 0.326713 | 0.009782 | 0.001696 | 8.00E-09 |
| Sedentary behavior | rs9867437 | 3:85676752 | C | A | 0.460371 | -0.0103 | 0.001602 | 1.30E-10 |
| Sedentary behavior | rs35007338 | 3:49671186 | T | C | 0.523754 | 0.009964 | 0.001588 | 3.50E-10 |
| Sedentary behavior | rs35797019 | 3:93987306 | G | A | 0.392335 | -0.00921 | 0.001628 | 1.50E-08 |
| Sedentary behavior | rs9834970 | 3:36856030 | C | T | 0.497804 | -0.00891 | 0.001587 | 2.00E-08 |
| Sedentary behavior | rs9880023 | 3:54178199 | T | G | 0.556427 | 0.009647 | 0.001608 | 2.00E-09 |
| Sedentary behavior | rs77719387 | 3:49917021 | A | T | 0.017691 | 0.039189 | 0.006409 | 9.70E-10 |
| Sedentary behavior | rs2352984 | 3:49948728 | C | T | 0.430943 | 0.019757 | 0.001604 | 7.40E-35 |
| Sedentary behavior | rs114600294 | 3:181419367 | C | G | 0.20804 | 0.010821 | 0.001955 | 3.10E-08 |
| Sedentary behavior | rs56398417 | 3:88024986 | T | C | 0.310889 | -0.00977 | 0.001718 | 1.30E-08 |
| Sedentary behavior | rs11714337 | 3:71582521 | A | G | 0.430094 | -0.00991 | 0.001611 | 7.60E-10 |
| Sedentary behavior | rs60737686 | 3:83002551 | G | A | 0.156124 | -0.01222 | 0.002205 | 3.00E-08 |
| Sedentary behavior | rs9867121 | 3:114631548 | A | C | 0.184147 | -0.0117 | 0.00206 | 1.40E-08 |
| Sedentary behavior | rs6850494 | 4:82291771 | C | A | 0.386016 | 0.00966 | 0.001634 | 3.40E-09 |
| Sedentary behavior | rs13107325 | 4:103188709 | T | C | 0.075099 | 0.02032 | 0.003018 | 1.70E-11 |
| Sedentary behavior | rs362312 | 4:3237644 | C | T | 0.424168 | -0.00952 | 0.001612 | 3.50E-09 |
| Sedentary behavior | rs34811474 | 4:25408838 | A | G | 0.230691 | -0.01316 | 0.001887 | 3.10E-12 |
| Sedentary behavior | rs11938781 | 4:17924734 | C | T | 0.165734 | 0.01287 | 0.002145 | 2.00E-09 |
| Sedentary behavior | rs6814554 | 4:152454334 | A | G | 0.47436 | 0.013528 | 0.001596 | 2.40E-17 |
| Sedentary behavior | rs2646351 | 4:55701312 | A | G | 0.452916 | 0.008914 | 0.001598 | 2.40E-08 |
| Sedentary behavior | rs6895658 | 5:124274035 | C | T | 0.193239 | -0.01298 | 0.002013 | 1.10E-10 |
| Sedentary behavior | rs262890 | 5:62930015 | G | A | 0.298989 | 0.013239 | 0.001738 | 2.60E-14 |
| Sedentary behavior | rs114755463 | 5:152503110 | A | G | 0.16775 | 0.013259 | 0.002148 | 6.70E-10 |
| Sedentary behavior | rs4554203 | 5:147886011 | A | G | 0.419724 | -0.00986 | 0.001613 | 9.90E-10 |
| Sedentary behavior | rs4110177 | 5:88793281 | A | G | 0.367281 | 0.009427 | 0.001657 | 1.30E-08 |
| Sedentary behavior | rs11242455 | 5:138568379 | T | C | 0.727097 | 0.010042 | 0.00181 | 2.90E-08 |
| Sedentary behavior | rs2283 | 5:106773623 | G | A | 0.339277 | -0.00927 | 0.001676 | 3.20E-08 |
| Sedentary behavior | rs9326862 | 5:112054547 | T | A | 0.530752 | -0.00923 | 0.001593 | 6.80E-09 |
| Sedentary behavior | rs996234 | 5:59455212 | A | G | 0.516342 | -0.01054 | 0.001648 | 1.60E-10 |
| Sedentary behavior | rs249960 | 5:96164771 | G | A | 0.182119 | -0.0118 | 0.002066 | 1.10E-08 |
| Sedentary behavior | rs1826510 | 5:24800012 | A | G | 0.828498 | -0.01273 | 0.002264 | 1.90E-08 |
| Sedentary behavior | rs1563908 | 5:60437381 | G | A | 0.378034 | -0.01027 | 0.001642 | 4.00E-10 |
| Sedentary behavior | rs9375292 | 6:98661205 | A | T | 0.268357 | -0.01188 | 0.001795 | 3.70E-11 |
| Sedentary behavior | rs9471333 | 6:40362023 | T | C | 0.552259 | -0.01043 | 0.001598 | 6.70E-11 |
| Sedentary behavior | rs6929983 | 6:43249411 | T | C | 0.166161 | 0.011887 | 0.002137 | 2.70E-08 |
| Sedentary behavior | rs58541850 | 6:166165563 | A | G | 0.058526 | 0.02139 | 0.003387 | 2.70E-10 |
| Sedentary behavior | rs4339469 | 6:98369230 | G | T | 0.62921 | 0.012529 | 0.001647 | 2.80E-14 |
| Sedentary behavior | rs319068 | 6:107788546 | C | T | 0.549179 | -0.0089 | 0.0016 | 2.60E-08 |
| Sedentary behavior | rs12214364 | 6:67556372 | G | T | 0.415458 | 0.009046 | 0.001645 | 3.80E-08 |
| Sedentary behavior | rs17789218 | 6:100600097 | C | T | 0.243949 | -0.01092 | 0.001849 | 3.60E-09 |
| Sedentary behavior | rs2857693 | 6:31588384 | T | G | 0.365548 | 0.010658 | 0.001646 | 9.60E-11 |
| Sedentary behavior | rs75499503 | 6:26145217 | T | C | 0.219683 | -0.01843 | 0.001948 | 3.10E-21 |
| Sedentary behavior | rs10269099 | 7:126371011 | T | G | 0.391234 | 0.009149 | 0.001632 | 2.10E-08 |
| Sedentary behavior | rs2106164 | 7:92661753 | C | T | 0.531731 | -0.00939 | 0.001598 | 4.20E-09 |
| Sedentary behavior | rs2240857 | 7:8010634 | G | T | 0.141014 | 0.015931 | 0.002302 | 4.50E-12 |
| Sedentary behavior | rs7798292 | 7:112974602 | A | G | 0.43452 | -0.00972 | 0.001603 | 1.30E-09 |
| Sedentary behavior | rs10109061 | 8:144239859 | G | A | 0.443183 | -0.00897 | 0.001619 | 3.10E-08 |
| Sedentary behavior | rs6994132 | 8:92653740 | C | T | 0.579236 | -0.00996 | 0.001611 | 6.20E-10 |
| Sedentary behavior | rs2725371 | 8:30854033 | G | A | 0.695652 | -0.01326 | 0.001735 | 2.10E-14 |
| Sedentary behavior | rs73560982 | 8:34214305 | C | T | 0.059951 | -0.01866 | 0.003349 | 2.50E-08 |
| Sedentary behavior | rs72673939 | 8:118867693 | C | G | 0.183051 | 0.0121 | 0.002058 | 4.10E-09 |
| Sedentary behavior | rs34094119 | 8:10935898 | G | A | 0.525005 | 0.009082 | 0.001596 | 1.30E-08 |
| Sedentary behavior | rs12553324 | 9:23347865 | G | C | 0.415158 | -0.01356 | 0.001615 | 4.50E-17 |
| Sedentary behavior | rs2073869 | 9:135763816 | T | C | 0.166613 | -0.0138 | 0.002139 | 1.10E-10 |
| Sedentary behavior | rs73571431 | 9:126136139 | T | C | 0.109769 | 0.015838 | 0.002553 | 5.50E-10 |
| Sedentary behavior | rs10739499 | 9:120514261 | G | C | 0.662566 | -0.00924 | 0.001685 | 4.20E-08 |
| Sedentary behavior | rs507021 | 9:22477134 | T | C | 0.57091 | 0.008812 | 0.001609 | 4.30E-08 |
| Sedentary behavior | rs494566 | 9:1785717 | T | C | 0.332086 | 0.010166 | 0.001696 | 2.00E-09 |
| Sedentary behavior | rs3138499 | 9:92219921 | C | A | 0.518183 | 0.010964 | 0.001605 | 8.50E-12 |
| Sedentary behavior | rs34864022 | 9:22609110 | G | A | 0.065324 | 0.021279 | 0.003232 | 4.60E-11 |
| Sedentary behavior | rs11245482 | 10:126733546 | C | T | 0.385493 | 0.010189 | 0.001636 | 4.80E-10 |
| Sedentary behavior | rs7921305 | 10:133775196 | A | G | 0.252545 | -0.01147 | 0.001831 | 3.70E-10 |
| Sedentary behavior | rs61864793 | 10:85803372 | C | T | 0.251695 | -0.01114 | 0.001834 | 1.20E-09 |
| Sedentary behavior | rs7089973 | 10:116569565 | A | C | 0.380267 | 0.009295 | 0.001646 | 1.60E-08 |
| Sedentary behavior | rs7899206 | 10:127188859 | G | T | 0.492382 | -0.00978 | 0.00161 | 1.30E-09 |
| Sedentary behavior | rs11191129 | 10:103606543 | T | C | 0.422818 | -0.00985 | 0.001609 | 9.30E-10 |
| Sedentary behavior | rs4747438 | 10:22124263 | T | C | 0.676512 | -0.01182 | 0.001703 | 3.90E-12 |
| Sedentary behavior | rs1291871 | 10:11086083 | C | T | 0.514085 | 0.009162 | 0.001598 | 9.90E-09 |
| Sedentary behavior | rs10765776 | 11:95656364 | A | C | 0.390712 | -0.01132 | 0.001638 | 4.80E-12 |
| Sedentary behavior | rs801733 | 11:65934549 | C | A | 0.357688 | -0.01249 | 0.001659 | 5.00E-14 |
| Sedentary behavior | rs11222919 | 11:131969663 | G | T | 0.175374 | -0.01221 | 0.002102 | 6.30E-09 |
| Sedentary behavior | rs115608101 | 11:107106532 | T | C | 0.130399 | -0.01354 | 0.002371 | 1.10E-08 |
| Sedentary behavior | rs77273138 | 12:24092043 | A | T | 0.045412 | 0.023477 | 0.00384 | 9.70E-10 |
| Sedentary behavior | rs1727332 | 12:123718301 | T | C | 0.753551 | 0.013151 | 0.001849 | 1.10E-12 |
| Sedentary behavior | rs9300594 | 13:100869905 | G | A | 0.253582 | 0.011202 | 0.001829 | 9.10E-10 |
| Sedentary behavior | rs1889996 | 13:54269950 | G | T | 0.738965 | 0.011968 | 0.001811 | 3.90E-11 |
| Sedentary behavior | rs147067 | 13:60434201 | A | G | 0.754854 | 0.010392 | 0.001855 | 2.10E-08 |
| Sedentary behavior | rs2479968 | 13:111969328 | G | A | 0.05112 | 0.020589 | 0.003702 | 2.70E-08 |
| Sedentary behavior | rs178203 | 14:26959322 | C | T | 0.749551 | 0.012014 | 0.001835 | 5.90E-11 |
| Sedentary behavior | rs2185490 | 14:69732119 | C | A | 0.617654 | 0.009373 | 0.001645 | 1.20E-08 |
| Sedentary behavior | rs78227853 | 15:44169073 | T | C | 0.024787 | -0.02836 | 0.005139 | 3.40E-08 |
| Sedentary behavior | rs79373894 | 15:73369053 | C | T | 0.034386 | -0.02873 | 0.00444 | 9.80E-11 |
| Sedentary behavior | rs6493583 | 15:53096084 | G | C | 0.137725 | 0.013362 | 0.002328 | 9.50E-09 |
| Sedentary behavior | rs4076457 | 15:78007213 | T | C | 0.256946 | -0.01004 | 0.001824 | 3.70E-08 |
| Sedentary behavior | rs749671 | 16:31088347 | A | G | 0.371805 | -0.01108 | 0.001645 | 1.60E-11 |
| Sedentary behavior | rs7184800 | 16:53509131 | A | G | 0.302842 | -0.01345 | 0.00173 | 7.40E-15 |
| Sedentary behavior | rs4788616 | 16:72211984 | G | T | 0.391065 | -0.01018 | 0.001631 | 4.30E-10 |
| Sedentary behavior | rs898751 | 17:2291863 | T | C | 0.493393 | 0.010021 | 0.001591 | 3.00E-10 |
| Sedentary behavior | rs184332798 | 18:53373610 | A | G | 0.026113 | -0.02777 | 0.005003 | 2.80E-08 |
| Sedentary behavior | rs11662211 | 18:77618869 | T | C | 0.505628 | -0.00945 | 0.001591 | 2.90E-09 |
| Sedentary behavior | rs11877758 | 18:35138110 | G | T | 0.312562 | 0.011075 | 0.001724 | 1.30E-10 |
| Sedentary behavior | rs111901094 | 19:19513570 | T | G | 0.182164 | 0.012711 | 0.002098 | 1.40E-09 |
| Sedentary behavior | rs6511708 | 19:10788813 | C | T | 0.664796 | -0.01208 | 0.001685 | 7.50E-13 |
| Sedentary behavior | rs76608582 | 19:4474725 | A | C | 0.046861 | -0.02183 | 0.003953 | 3.40E-08 |
| Sedentary behavior | rs61743199 | 19:50161091 | G | A | 0.071839 | 0.017384 | 0.003079 | 1.60E-08 |
| Sedentary behavior | rs204092 | 20:14732585 | C | T | 0.623782 | -0.00952 | 0.001657 | 9.00E-09 |
| Sedentary behavior | rs6103265 | 20:42012125 | A | G | 0.159307 | -0.01213 | 0.002171 | 2.30E-08 |
| Sedentary behavior | rs4911257 | 20:31359574 | C | T | 0.393375 | 0.009747 | 0.001632 | 2.30E-09 |
| Sedentary behavior | rs6102912 | 20:41202935 | C | T | 0.41003 | -0.01091 | 0.001615 | 1.40E-11 |
| Sedentary behavior | rs11700249 | 20:11910800 | G | T | 0.408795 | 0.009433 | 0.001621 | 5.90E-09 |
| Sedentary behavior | rs60387034 | 20:40010670 | G | C | 0.268725 | 0.009914 | 0.001801 | 3.70E-08 |
| Sedentary behavior | rs11696187 | 20:58891882 | T | C | 0.160229 | -0.01357 | 0.002171 | 4.20E-10 |
| Sedentary behavior | rs3810496 | 20:62406886 | C | T | 0.61619 | 0.009371 | 0.001644 | 1.20E-08 |
| Sedentary behavior | rs6125907 | 20:48730315 | A | C | 0.092033 | 0.016182 | 0.00276 | 4.50E-09 |
| Sedentary behavior | rs6031440 | 20:42809317 | T | A | 0.602325 | -0.00894 | 0.001627 | 3.90E-08 |
| Sedentary behavior | rs11911112 | 21:40528346 | C | A | 0.365887 | -0.01074 | 0.001653 | 8.00E-11 |
| Smoking Initiation | rs12027999 | 1:154206358 | C | T | 0.12 | -0.0143 | 0.00244 | 5.33E-10 |
| Smoking Initiation | rs45444697 | 1:155034632 | G | C | 0.212 | 0.00955 | 0.00151 | 2.72E-10 |
| Smoking Initiation | rs2901785 | 1:174104743 | A | G | 0.446 | -0.00816 | 0.00161 | 1.47E-11 |
| Smoking Initiation | rs147052174 | 1:179783167 | T | G | 0.0171 | 0.0368 | 0.00631 | 2.30E-10 |
| Smoking Initiation | rs3820277 | 1:18436657 | T | G | 0.526 | -0.0098 | 0.00162 | 1.57E-13 |
| Smoking Initiation | rs35656245 | 1:190957480 | A | G | 0.276 | 0.00925 | 0.00179 | 2.23E-08 |
| Smoking Initiation | rs12739243 | 1:210302043 | C | T | 0.221 | -0.0122 | 0.00192 | 4.45E-12 |
| Smoking Initiation | rs12563365 | 1:236872829 | A | G | 0.556 | 0.00927 | 0.00162 | 1.05E-10 |
| Smoking Initiation | rs876793 | 1:237852083 | C | T | 0.349262 | -0.00861 | 0.001315 | 5.69E-11 |
| Smoking Initiation | rs1889571 | 1:32195819 | G | T | 0.131 | 0.0132 | 0.00236 | 4.19E-09 |
| Smoking Initiation | rs10914684 | 1:33795572 | A | G | 0.324 | -0.00836 | 0.00172 | 6.32E-09 |
| Smoking Initiation | rs2637869 | 1:38757237 | A | G | 0.297 | 0.00893 | 0.00174 | 6.54E-11 |
| Smoking Initiation | rs12755632 | 1:41776623 | G | A | 0.316 | -0.00798 | 0.00172 | 1.93E-08 |
| Smoking Initiation | rs951740 | 1:44011737 | A | G | 0.625 | 0.0163 | 0.00166 | 3.82E-29 |
| Smoking Initiation | rs925524 | 1:46496709 | G | A | 0.71 | 0.00814 | 0.00176 | 2.94E-08 |
| Smoking Initiation | rs12022778 | 1:50603995 | C | A | 0.202 | 0.015 | 0.00198 | 3.18E-17 |
| Smoking Initiation | rs11587399 | 1:50861071 | T | A | 0.221 | -0.00858 | 0.00148 | 7.25E-09 |
| Smoking Initiation | rs4912332 | 1:58815243 | T | C | 0.491 | 0.00845 | 0.0016 | 2.94E-08 |
| Smoking Initiation | rs1937443 | 1:66469643 | G | C | 0.563 | 0.0129 | 0.00162 | 1.79E-15 |
| Smoking Initiation | rs1022528 | 1:71490122 | A | G | 0.344 | 0.00837 | 0.00129 | 8.48E-11 |
| Smoking Initiation | rs12740789 | 1:72752073 | A | G | 0.178 | -0.0137 | 0.001597 | 1.18E-17 |
| Smoking Initiation | rs80054503 | 1:72900406 | C | T | 0.116 | -0.0128 | 0.002162 | 3.10E-09 |
| Smoking Initiation | rs10789369 | 1:73824909 | G | A | 0.615 | -0.013 | 0.00165 | 3.39E-19 |
| Smoking Initiation | rs1514176 | 1:74991596 | A | G | 0.58 | -0.0107 | 0.00162 | 7.67E-14 |
| Smoking Initiation | rs10873871 | 1:76689019 | G | A | 0.207 | 0.0098 | 0.00199 | 2.82E-08 |
| Smoking Initiation | rs12130857 | 1:7791461 | A | G | 0.324968 | -0.00867 | 0.001309 | 3.65E-11 |
| Smoking Initiation | rs301807 | 1:8484823 | G | A | 0.57 | 0.0112 | 0.00163 | 2.50E-12 |
| Smoking Initiation | rs11162019 | 1:87913176 | T | C | 0.363 | -0.00932 | 0.00166 | 5.06E-09 |
| Smoking Initiation | rs1008078 | 1:91189731 | T | C | 0.402 | 0.0119 | 0.00164 | 1.63E-18 |
| Smoking Initiation | rs1935571 | 1:96414335 | G | T | 0.48 | -0.0104 | 0.0016 | 6.99E-10 |
| Smoking Initiation | rs7920501 | 10:10043159 | A | T | 0.465 | -0.00911 | 0.0016 | 1.25E-09 |
| Smoking Initiation | rs7901883 | 10:103186838 | A | G | 0.230322 | -0.00934 | 0.001467 | 1.98E-10 |
| Smoking Initiation | rs11594623 | 10:103960351 | C | T | 0.234241 | 0.0132 | 0.00144 | 7.45E-20 |
| Smoking Initiation | rs11191269 | 10:104120522 | G | C | 0.193294 | 0.00855 | 0.00156 | 4.61E-08 |
| Smoking Initiation | rs28408682 | 10:104403310 | G | A | 0.600019 | 0.00805 | 0.001255 | 1.41E-10 |
| Smoking Initiation | rs12244388 | 10:104640052 | A | G | 0.35 | 0.0137 | 0.00169 | 4.31E-22 |
| Smoking Initiation | rs111842178 | 10:104852121 | G | A | 0.231008 | 0.0129 | 0.001841 | 2.24E-12 |
| Smoking Initiation | rs34970111 | 10:106078937 | T | C | 0.458 | -0.00718 | 0.001262 | 1.28E-08 |
| Smoking Initiation | rs9787523 | 10:106460460 | C | T | 0.418 | -0.00883 | 0.00165 | 1.42E-09 |
| Smoking Initiation | rs11192347 | 10:106929313 | A | G | 0.104 | -0.0129 | 0.002084 | 6.15E-10 |
| Smoking Initiation | rs1291821 | 10:11133823 | G | A | 0.534 | 0.00807 | 0.00161 | 1.39E-08 |
| Smoking Initiation | rs10885480 | 10:115378364 | C | T | 0.284 | -0.00953 | 0.00178 | 3.83E-11 |
| Smoking Initiation | rs4752018 | 10:118678712 | A | C | 0.231 | 0.0107 | 0.0019 | 4.42E-10 |
| Smoking Initiation | rs9423279 | 10:125680419 | G | C | 0.645 | -0.0102 | 0.00183 | 3.06E-12 |
| Smoking Initiation | rs11258417 | 10:13533053 | T | C | 0.391 | -0.00852 | 0.00163 | 2.71E-08 |
| Smoking Initiation | rs7072776 | 10:22032942 | G | A | 0.712 | -0.0138 | 0.00179 | 5.66E-15 |
| Smoking Initiation | rs2796793 | 10:36634124 | A | G | 0.452 | 0.0082 | 0.00161 | 1.55E-08 |
| Smoking Initiation | rs1733760 | 10:56698174 | C | T | 0.51 | 0.00821 | 0.00161 | 6.70E-09 |
| Smoking Initiation | rs7921378 | 10:63674885 | C | G | 0.482 | -0.0142 | 0.00161 | 6.10E-20 |
| Smoking Initiation | rs10905461 | 10:8803551 | C | T | 0.748 | -0.0104 | 0.00186 | 2.36E-08 |
| Smoking Initiation | rs76460663 | 11:111979741 | G | C | 0.041056 | -0.0226 | 0.003426 | 4.15E-11 |
| Smoking Initiation | rs2155646 | 11:112912811 | C | T | 0.4 | 0.021 | 0.00163 | 9.44E-48 |
| Smoking Initiation | rs1713676 | 11:113660576 | G | A | 0.522512 | -0.00802 | 0.00122 | 5.38E-11 |
| Smoking Initiation | rs238896 | 11:113994505 | A | G | 0.49 | -0.00989 | 0.00162 | 3.65E-11 |
| Smoking Initiation | rs540860 | 11:121530888 | G | A | 0.543 | 0.0101 | 0.00161 | 5.75E-12 |
| Smoking Initiation | rs1944689 | 11:121634334 | T | G | 0.785911 | 0.00859 | 0.00151 | 1.27E-08 |
| Smoking Initiation | rs1834306 | 11:122023187 | G | A | 0.579399 | -0.00711 | 0.00127 | 1.96E-08 |
| Smoking Initiation | rs1106363 | 11:131966264 | T | C | 0.344579 | 0.00833 | 0.00129 | 9.20E-11 |
| Smoking Initiation | rs2010921 | 11:132098205 | A | G | 0.311 | 0.00897 | 0.00174 | 2.47E-10 |
| Smoking Initiation | rs6265 | 11:27679916 | T | C | 0.188 | -0.0165 | 0.00204 | 2.81E-19 |
| Smoking Initiation | rs4275621 | 11:28652996 | G | A | 0.382 | -0.0103 | 0.00126 | 3.76E-16 |
| Smoking Initiation | rs62618693 | 11:32956492 | T | C | 0.0428 | -0.0204 | 0.00415 | 2.09E-08 |
| Smoking Initiation | rs2939756 | 11:41436297 | A | G | 0.48 | -0.00908 | 0.00161 | 7.45E-10 |
| Smoking Initiation | rs1381775 | 11:42442826 | C | T | 0.712 | -0.00754 | 0.00177 | 2.79E-08 |
| Smoking Initiation | rs2959084 | 11:46078656 | A | G | 0.704674 | 0.00817 | 0.00134 | 9.82E-10 |
| Smoking Initiation | rs3740977 | 11:46393574 | C | T | 0.167 | 0.0103 | 0.00216 | 1.17E-08 |
| Smoking Initiation | rs61886926 | 11:64133552 | T | C | 0.384 | -0.00987 | 0.00163 | 7.30E-12 |
| Smoking Initiation | rs61884449 | 11:64485193 | T | C | 0.149183 | 0.00962 | 0.001722 | 2.32E-08 |
| Smoking Initiation | rs644740 | 11:65561468 | T | C | 0.457 | -0.00839 | 0.00161 | 3.67E-08 |
| Smoking Initiation | rs7943721 | 11:73309393 | A | G | 0.829 | -0.0117 | 0.00218 | 3.58E-10 |
| Smoking Initiation | rs7929518 | 11:85980958 | G | A | 0.773 | 0.0119 | 0.00192 | 2.55E-10 |
| Smoking Initiation | rs586699 | 11:92289734 | A | G | 0.543 | -0.0078 | 0.0016 | 7.29E-09 |
| Smoking Initiation | rs77215829 | 12:112618346 | C | A | 0.131 | -0.013 | 0.00243 | 2.02E-10 |
| Smoking Initiation | rs1109480 | 12:121083279 | A | G | 0.384 | -0.0101 | 0.00167 | 1.84E-10 |
| Smoking Initiation | rs11611651 | 12:133380790 | A | G | 0.0868 | 0.0141 | 0.0028 | 2.05E-09 |
| Smoking Initiation | rs11057005 | 12:16748721 | G | A | 0.441 | -0.0104 | 0.00163 | 9.12E-10 |
| Smoking Initiation | rs13906 | 12:49952394 | T | C | 0.109 | -0.0118 | 0.00253 | 1.98E-09 |
| Smoking Initiation | rs4759229 | 12:56474480 | G | A | 0.656 | 0.01 | 0.00168 | 6.53E-09 |
| Smoking Initiation | rs7969559 | 12:69655167 | G | A | 0.713 | -0.0097 | 0.00178 | 1.53E-09 |
| Smoking Initiation | rs7134009 | 12:75263193 | C | T | 0.287 | -0.00904 | 0.00178 | 4.30E-08 |
| Smoking Initiation | rs7333559 | 13:100546450 | A | G | 0.783 | -0.0127 | 0.002 | 5.94E-14 |
| Smoking Initiation | rs1108130 | 13:100648356 | A | T | 0.212 | 0.0116 | 0.00151 | 1.57E-14 |
| Smoking Initiation | rs12855717 | 13:101252635 | T | C | 0.538 | 0.00753 | 0.00124 | 1.22E-09 |
| Smoking Initiation | rs17197663 | 13:38172867 | A | G | 0.125 | -0.0117 | 0.00245 | 2.06E-08 |
| Smoking Initiation | rs4264267 | 13:38359676 | T | C | 0.527042 | 0.00713 | 0.00123 | 6.82E-09 |
| Smoking Initiation | rs61959481 | 13:55834929 | A | G | 0.21 | -0.0113 | 0.00203 | 7.95E-11 |
| Smoking Initiation | rs3098272 | 13:55931424 | C | A | 0.798794 | -0.00867 | 0.001547 | 2.08E-08 |
| Smoking Initiation | rs9538162 | 13:59265043 | C | T | 0.415874 | 0.0085 | 0.001263 | 1.76E-11 |
| Smoking Initiation | rs1413119 | 13:59339281 | T | C | 0.396318 | -0.00734 | 0.001254 | 4.77E-09 |
| Smoking Initiation | rs56367474 | 13:59454139 | T | C | 0.304 | -0.00873 | 0.00175 | 4.20E-10 |
| Smoking Initiation | rs55786907 | 13:59871584 | G | A | 0.162492 | 0.00931 | 0.001654 | 1.84E-08 |
| Smoking Initiation | rs4886207 | 13:60705792 | C | T | 0.637 | -0.00872 | 0.00166 | 8.78E-10 |
| Smoking Initiation | rs9540731 | 13:66949370 | T | C | 0.509 | -0.0094 | 0.0016 | 3.42E-12 |
| Smoking Initiation | rs9545155 | 13:80191873 | C | T | 0.478 | -0.00889 | 0.0016 | 3.04E-10 |
| Smoking Initiation | rs1772572 | 13:81191176 | A | C | 0.324132 | -0.00807 | 0.001301 | 5.62E-10 |
| Smoking Initiation | rs75674569 | 13:96823724 | A | G | 0.0997 | -0.0125 | 0.00268 | 2.58E-09 |
| Smoking Initiation | rs12878369 | 14:28346502 | A | C | 0.414762 | 0.00836 | 0.00124 | 1.60E-11 |
| Smoking Initiation | rs9323328 | 14:58653514 | G | A | 0.537 | -0.00818 | 0.00162 | 2.55E-08 |
| Smoking Initiation | rs1811739 | 14:77529375 | A | G | 0.248 | 0.0112 | 0.00186 | 5.97E-10 |
| Smoking Initiation | rs8005334 | 14:79563654 | G | T | 0.36 | 0.0104 | 0.00168 | 3.44E-10 |
| Smoking Initiation | rs34940743 | 14:80102233 | G | A | 0.346 | 0.00773 | 0.0013 | 2.80E-09 |
| Smoking Initiation | rs2925128 | 14:98362355 | T | C | 0.385191 | 0.00805 | 0.00128 | 3.67E-10 |
| Smoking Initiation | rs1381287 | 14:98597552 | T | C | 0.467 | 0.00984 | 0.00163 | 1.81E-12 |
| Smoking Initiation | rs55913542 | 14:99693843 | T | G | 0.175 | 0.011 | 0.00211 | 3.25E-08 |
| Smoking Initiation | rs1435672 | 15:36399479 | C | T | 0.56 | 0.00784 | 0.00161 | 3.82E-08 |
| Smoking Initiation | rs281296 | 15:47685010 | A | G | 0.357 | 0.0143 | 0.00168 | 1.59E-20 |
| Smoking Initiation | rs1435741 | 15:47935843 | A | G | 0.432951 | 0.00904 | 0.00127 | 1.09E-12 |
| Smoking Initiation | rs56902655 | 15:63898709 | G | T | 0.136 | -0.0113 | 0.00235 | 4.09E-09 |
| Smoking Initiation | rs2289791 | 15:67476952 | T | G | 0.247 | -0.00884 | 0.00189 | 2.01E-09 |
| Smoking Initiation | rs60833441 | 15:74048768 | G | A | 0.461 | -0.00683 | 0.0016 | 2.28E-08 |
| Smoking Initiation | rs62007780 | 15:78025464 | T | G | 0.416 | -0.00787 | 0.00163 | 7.48E-10 |
| Smoking Initiation | rs4310804 | 15:96858409 | G | C | 0.247 | -0.0104 | 0.00187 | 7.55E-10 |
| Smoking Initiation | rs8027457 | 15:99204101 | C | T | 0.511 | 0.00869 | 0.0016 | 1.88E-09 |
| Smoking Initiation | rs7192140 | 16:10173748 | C | T | 0.498 | -0.00906 | 0.0016 | 3.40E-11 |
| Smoking Initiation | rs9922607 | 16:17570220 | T | C | 0.2 | -0.0122 | 0.00207 | 3.42E-12 |
| Smoking Initiation | rs9941217 | 16:18050926 | G | C | 0.352204 | -0.00892 | 0.00128 | 3.50E-12 |
| Smoking Initiation | rs7188873 | 16:24727064 | G | A | 0.612998 | 0.00972 | 0.001253 | 8.46E-15 |
| Smoking Initiation | rs6497840 | 16:25351633 | A | G | 0.707 | 0.0103 | 0.00178 | 2.01E-15 |
| Smoking Initiation | rs4785187 | 16:49766772 | A | G | 0.223 | 0.0106 | 0.00194 | 6.55E-11 |
| Smoking Initiation | rs8050598 | 16:49891964 | T | C | 0.254117 | 0.00941 | 0.00147 | 1.76E-10 |
| Smoking Initiation | rs12918191 | 16:50945156 | G | A | 0.243 | -0.0108 | 0.00188 | 3.14E-11 |
| Smoking Initiation | rs11076962 | 16:5811367 | C | T | 0.279 | 0.0101 | 0.0018 | 1.20E-10 |
| Smoking Initiation | rs9302604 | 16:69576894 | G | A | 0.435 | 0.0109 | 0.00163 | 3.29E-13 |
| Smoking Initiation | rs1139897 | 16:720986 | A | G | 0.23 | -0.0123 | 0.00188 | 1.77E-15 |
| Smoking Initiation | rs9936784 | 16:72230694 | G | T | 0.53418 | 0.00682 | 0.00125 | 4.33E-08 |
| Smoking Initiation | rs62052916 | 16:72574550 | T | A | 0.0701 | -0.0147 | 0.00297 | 1.62E-10 |
| Smoking Initiation | rs4788676 | 16:72950468 | C | T | 0.228528 | -0.00852 | 0.00146 | 4.92E-09 |
| Smoking Initiation | rs61537885 | 16:75620118 | C | T | 0.037215 | -0.0199 | 0.00344 | 8.06E-09 |
| Smoking Initiation | rs117657830 | 16:75766873 | G | A | 0.0417 | -0.0193 | 0.00421 | 3.18E-09 |
| Smoking Initiation | rs1050847 | 16:87443734 | T | C | 0.559 | -0.0092 | 0.00165 | 7.37E-09 |
| Smoking Initiation | rs11642231 | 16:89608702 | A | G | 0.369 | -0.00864 | 0.00166 | 3.44E-09 |
| Smoking Initiation | rs11651955 | 17:16235462 | A | G | 0.499 | -0.00802 | 0.00161 | 3.74E-08 |
| Smoking Initiation | rs4790874 | 17:1995177 | T | C | 0.532 | 0.01 | 0.00163 | 8.43E-12 |
| Smoking Initiation | rs67777803 | 17:27323322 | T | G | 0.172 | -0.0136 | 0.00217 | 3.18E-13 |
| Smoking Initiation | rs2344976 | 17:30685935 | C | T | 0.612 | -0.0083 | 0.00164 | 7.98E-09 |
| Smoking Initiation | rs3764351 | 17:37824339 | A | G | 0.657 | -0.00842 | 0.0017 | 3.89E-08 |
| Smoking Initiation | rs17692129 | 17:44793283 | T | C | 0.331 | 0.0112 | 0.00185 | 4.57E-13 |
| Smoking Initiation | rs75919030 | 17:50193197 | C | T | 0.267 | -0.0114 | 0.0018 | 3.35E-13 |
| Smoking Initiation | rs2938134 | 17:50243397 | A | C | 0.673 | -0.00858 | 0.00136 | 3.14E-10 |
| Smoking Initiation | rs2587507 | 17:77790135 | C | T | 0.502 | -0.00799 | 0.00159 | 8.69E-09 |
| Smoking Initiation | rs11078713 | 17:7795972 | G | A | 0.419348 | -0.00714 | 0.00126 | 1.59E-08 |
| Smoking Initiation | rs28441558 | 17:7803118 | C | T | 0.0563 | -0.0175 | 0.00332 | 1.24E-10 |
| Smoking Initiation | rs4476253 | 18:25253297 | A | G | 0.24 | -0.0105 | 0.00188 | 5.78E-10 |
| Smoking Initiation | rs7505855 | 18:31696075 | T | C | 0.586 | -0.0103 | 0.00163 | 5.31E-11 |
| Smoking Initiation | rs8096225 | 18:36921851 | C | A | 0.703 | 0.0084 | 0.00175 | 2.63E-08 |
| Smoking Initiation | rs67050670 | 18:39297254 | G | A | 0.229 | -0.0107 | 0.00193 | 2.34E-11 |
| Smoking Initiation | rs2359180 | 18:41314171 | G | A | 0.369 | -0.00715 | 0.00206 | 4.98E-08 |
| Smoking Initiation | rs72898831 | 18:42658643 | G | A | 0.155 | -0.0151 | 0.00224 | 4.14E-12 |
| Smoking Initiation | rs8083764 | 18:49874515 | T | G | 0.306171 | -0.00778 | 0.00135 | 7.97E-09 |
| Smoking Initiation | rs1373178 | 18:49967811 | G | T | 0.588 | -0.011 | 0.00165 | 4.16E-15 |
| Smoking Initiation | rs62098013 | 18:50863861 | A | G | 0.365322 | 0.0087 | 0.0013 | 2.24E-11 |
| Smoking Initiation | rs72938304 | 18:53661743 | A | G | 0.113 | -0.0131 | 0.0026 | 1.36E-11 |
| Smoking Initiation | rs34342129 | 18:5872472 | C | T | 0.509 | -0.00727 | 0.00159 | 2.13E-08 |
| Smoking Initiation | rs11872397 | 18:72535282 | A | G | 0.253 | -0.0116 | 0.00187 | 5.20E-09 |
| Smoking Initiation | rs71367544 | 18:77574374 | T | C | 0.203 | 0.0104 | 0.00198 | 8.54E-11 |
| Smoking Initiation | rs113230003 | 19:18460956 | A | G | 0.255 | -0.0106 | 0.00189 | 1.05E-10 |
| Smoking Initiation | rs8103660 | 19:18566395 | C | T | 0.354431 | 0.00764 | 0.00129 | 3.03E-09 |
| Smoking Initiation | rs76608582 | 19:4474725 | A | C | 0.0489 | -0.027 | 0.00437 | 4.88E-09 |
| Smoking Initiation | rs10853981 | 19:4965064 | A | G | 0.33036 | 0.00713 | 0.00131 | 4.88E-08 |
| Smoking Initiation | rs117734003 | 19:51129745 | C | G | 0.0673 | 0.015 | 0.00338 | 2.57E-09 |
| Smoking Initiation | rs1126757 | 19:55879872 | T | C | 0.473 | 0.0077 | 0.00161 | 2.92E-08 |
| Smoking Initiation | rs13392222 | 2:100672408 | C | A | 0.139 | -0.0121 | 0.0023 | 1.93E-10 |
| Smoking Initiation | rs1901477 | 2:104126983 | G | A | 0.511 | 0.0164 | 0.0016 | 2.07E-31 |
| Smoking Initiation | rs11889814 | 2:104432494 | C | A | 0.128 | -0.0101 | 0.00184 | 3.44E-08 |
| Smoking Initiation | rs3811038 | 2:113240183 | C | T | 0.279 | 0.0102 | 0.00179 | 1.58E-11 |
| Smoking Initiation | rs75210106 | 2:113246436 | T | C | 0.176676 | -0.00918 | 0.00164 | 2.33E-08 |
| Smoking Initiation | rs34399632 | 2:137571174 | G | A | 0.232 | 0.0101 | 0.00185 | 1.46E-10 |
| Smoking Initiation | rs74697736 | 2:145412271 | A | G | 0.287239 | 0.0107 | 0.001357 | 2.43E-15 |
| Smoking Initiation | rs6756212 | 2:146140132 | T | C | 0.535 | -0.0188 | 0.0016 | 3.49E-40 |
| Smoking Initiation | rs16826827 | 2:147825689 | C | T | 0.124 | -0.0128 | 0.00243 | 9.17E-09 |
| Smoking Initiation | rs1445649 | 2:155682556 | C | T | 0.538 | 0.011 | 0.0016 | 8.48E-16 |
| Smoking Initiation | rs1722666 | 2:161816880 | T | C | 0.732 | 0.00771 | 0.001378 | 2.17E-08 |
| Smoking Initiation | rs11678980 | 2:162101261 | A | G | 0.45 | 0.00893 | 0.001295 | 5.19E-12 |
| Smoking Initiation | rs12474587 | 2:162802993 | T | G | 0.429 | 0.0129 | 0.00162 | 4.83E-21 |
| Smoking Initiation | rs357304 | 2:164862639 | C | T | 0.727 | 0.00933 | 0.00183 | 5.40E-09 |
| Smoking Initiation | rs13007361 | 2:166250244 | A | G | 0.208 | 0.0107 | 0.00201 | 2.29E-08 |
| Smoking Initiation | rs7600835 | 2:172521827 | A | G | 0.342 | -0.00868 | 0.00173 | 1.80E-08 |
| Smoking Initiation | rs6750529 | 2:182027603 | T | C | 0.744 | 0.0114 | 0.00184 | 9.26E-12 |
| Smoking Initiation | rs17229285 | 2:199523122 | T | C | 0.505 | -0.0103 | 0.0016 | 1.27E-09 |
| Smoking Initiation | rs3115418 | 2:200936399 | C | T | 0.454 | -0.00876 | 0.0016 | 2.79E-08 |
| Smoking Initiation | rs62193862 | 2:202843875 | A | G | 0.0999 | 0.0149 | 0.00279 | 1.99E-08 |
| Smoking Initiation | rs1022376 | 2:22067213 | C | T | 0.515821 | -0.0071 | 0.001258 | 1.66E-08 |
| Smoking Initiation | rs4674916 | 2:225365635 | A | C | 0.327671 | -0.00861 | 0.0013 | 3.06E-11 |
| Smoking Initiation | rs61533748 | 2:22582968 | C | T | 0.384 | 0.00915 | 0.00166 | 2.82E-11 |
| Smoking Initiation | rs4674993 | 2:226332033 | G | A | 0.2 | -0.0133 | 0.002 | 4.85E-14 |
| Smoking Initiation | rs114976176 | 2:264621 | C | A | 0.351571 | -0.00746 | 0.00128 | 6.04E-09 |
| Smoking Initiation | rs72790288 | 2:29513404 | A | G | 0.0282 | -0.0225 | 0.00483 | 3.28E-09 |
| Smoking Initiation | rs2710634 | 2:32808804 | C | T | 0.521 | -0.00942 | 0.00162 | 3.36E-12 |
| Smoking Initiation | rs62106258 | 2:417167 | C | T | 0.047329 | -0.0242 | 0.003195 | 3.33E-14 |
| Smoking Initiation | rs62137126 | 2:44250149 | G | A | 0.121094 | -0.0114 | 0.00188 | 1.31E-09 |
| Smoking Initiation | rs1004787 | 2:45159091 | A | G | 0.552 | 0.0152 | 0.00162 | 1.11E-28 |
| Smoking Initiation | rs7598402 | 2:50735943 | G | C | 0.492084 | -0.00709 | 0.00123 | 7.38E-09 |
| Smoking Initiation | rs10490159 | 2:51341259 | T | C | 0.394 | 0.0119 | 0.00164 | 3.86E-11 |
| Smoking Initiation | rs1518393 | 2:58171220 | C | A | 0.619 | 0.00988 | 0.00168 | 1.30E-10 |
| Smoking Initiation | rs17616642 | 2:59022210 | G | A | 0.24687 | -0.00797 | 0.00142 | 2.10E-08 |
| Smoking Initiation | rs6730325 | 2:59315828 | A | G | 0.609782 | -0.00701 | 0.00125 | 2.10E-08 |
| Smoking Initiation | rs2539706 | 2:59819545 | A | G | 0.529947 | 0.00789 | 0.00124 | 1.95E-10 |
| Smoking Initiation | rs7585579 | 2:60024857 | G | C | 0.499 | 0.00998 | 0.00163 | 5.48E-15 |
| Smoking Initiation | rs1863161 | 2:60139524 | A | G | 0.56094 | 0.00746 | 0.00125 | 2.34E-09 |
| Smoking Initiation | rs359247 | 2:60477052 | T | A | 0.638652 | 0.0105 | 0.00127 | 9.89E-17 |
| Smoking Initiation | rs6731872 | 2:624205 | G | T | 0.826 | 0.0182 | 0.00213 | 5.35E-21 |
| Smoking Initiation | rs62180324 | 2:63416606 | A | G | 0.212 | -0.0103 | 0.00193 | 3.91E-10 |
| Smoking Initiation | rs6750107 | 2:80748807 | A | G | 0.386875 | 0.00705 | 0.00127 | 2.60E-08 |
| Smoking Initiation | rs12714017 | 2:80999398 | C | T | 0.511 | 0.00877 | 0.00162 | 3.65E-09 |
| Smoking Initiation | rs56208390 | 2:83247997 | G | A | 0.123 | 0.011 | 0.00242 | 2.68E-08 |
| Smoking Initiation | rs11692435 | 2:98275354 | A | G | 0.0848 | 0.027734 | 0.071217 | 4.47E-08 |
| Smoking Initiation | rs6050446 | 20:25195509 | G | A | 0.971 | 0.0351 | 0.00488 | 8.80E-13 |
| Smoking Initiation | rs6058782 | 20:29946968 | T | C | 0.908 | 0.0144 | 0.00214 | 1.78E-11 |
| Smoking Initiation | rs1555445 | 20:31175258 | T | A | 0.318 | 0.0103 | 0.00174 | 7.75E-12 |
| Smoking Initiation | rs6073075 | 20:42015801 | A | T | 0.824 | -0.0108 | 0.00215 | 2.44E-08 |
| Smoking Initiation | rs910912 | 20:54462393 | C | T | 0.739 | -0.0112 | 0.00183 | 7.82E-09 |
| Smoking Initiation | rs6011779 | 20:61984317 | T | C | 0.806 | -0.0104 | 0.00206 | 2.83E-09 |
| Smoking Initiation | rs3810496 | 20:62406886 | C | T | 0.619436 | 0.00785 | 0.0013 | 1.54E-09 |
| Smoking Initiation | rs4818005 | 21:40588819 | A | G | 0.581 | -0.0106 | 0.00164 | 1.09E-14 |
| Smoking Initiation | rs139896 | 22:38397797 | C | T | 0.648 | 0.00891 | 0.00168 | 7.14E-09 |
| Smoking Initiation | rs4822102 | 22:42698430 | T | C | 0.618 | -0.0083 | 0.00163 | 2.78E-10 |
| Smoking Initiation | rs9627272 | 22:46442288 | C | G | 0.407 | -0.0103 | 0.00175 | 2.42E-09 |
| Smoking Initiation | rs6437769 | 3:107997514 | T | C | 0.581 | 0.00841 | 0.00161 | 3.74E-08 |
| Smoking Initiation | rs9288999 | 3:114147927 | A | G | 0.735 | 0.0105 | 0.00183 | 1.50E-09 |
| Smoking Initiation | rs6438436 | 3:117822149 | T | C | 0.816 | 0.0151 | 0.00209 | 5.33E-14 |
| Smoking Initiation | rs12053870 | 3:118302515 | G | T | 0.541511 | 0.00754 | 0.00123 | 1.02E-09 |
| Smoking Initiation | rs9826984 | 3:131945722 | A | G | 0.542 | -0.0085 | 0.00161 | 3.87E-08 |
| Smoking Initiation | rs2279829 | 3:147106319 | T | C | 0.216 | -0.0087 | 0.00194 | 2.05E-08 |
| Smoking Initiation | rs2319545 | 3:147719648 | A | C | 0.149099 | 0.0112 | 0.00173 | 8.30E-11 |
| Smoking Initiation | rs10935779 | 3:149543102 | T | C | 0.415 | -0.00777 | 0.00162 | 2.95E-08 |
| Smoking Initiation | rs963354 | 3:157393770 | A | C | 0.687 | 0.00733 | 0.001337 | 4.21E-08 |
| Smoking Initiation | rs1714521 | 3:158284861 | C | A | 0.411 | -0.00978 | 0.00164 | 3.07E-10 |
| Smoking Initiation | rs1449012 | 3:159048333 | T | C | 0.463 | -0.00747 | 0.00124 | 1.77E-09 |
| Smoking Initiation | rs9850597 | 3:161761866 | A | G | 0.816 | -0.0103 | 0.00207 | 1.65E-08 |
| Smoking Initiation | rs748832 | 3:16851202 | G | A | 0.371 | 0.00931 | 0.00165 | 6.60E-11 |
| Smoking Initiation | rs1187820 | 3:173072584 | T | C | 0.439 | -0.00706 | 0.00127 | 2.69E-08 |
| Smoking Initiation | rs16828799 | 3:173353739 | T | G | 0.156 | 0.00986 | 0.00215 | 1.83E-08 |
| Smoking Initiation | rs9841807 | 3:175718927 | T | C | 0.273 | 0.00919 | 0.00179 | 1.35E-08 |
| Smoking Initiation | rs7631379 | 3:181409057 | C | T | 0.206 | 0.0117 | 0.00206 | 3.94E-11 |
| Smoking Initiation | rs11713899 | 3:2365026 | C | A | 0.171 | 0.0108 | 0.00215 | 3.15E-08 |
| Smoking Initiation | rs10446419 | 3:25725501 | G | A | 0.207 | -0.011 | 0.00208 | 5.05E-10 |
| Smoking Initiation | rs13319205 | 3:47800216 | A | T | 0.29 | 0.00804 | 0.00136 | 3.77E-09 |
| Smoking Initiation | rs3172494 | 3:48731487 | T | G | 0.115 | -0.014 | 0.00193 | 3.40E-13 |
| Smoking Initiation | rs2526390 | 3:50192760 | T | C | 0.334 | 0.0116 | 0.00171 | 3.62E-14 |
| Smoking Initiation | rs2276825 | 3:52886605 | C | T | 0.245 | 0.0112 | 0.00188 | 1.89E-10 |
| Smoking Initiation | rs2306866 | 3:53766212 | T | A | 0.614 | -0.00801 | 0.001257 | 1.89E-10 |
| Smoking Initiation | rs73831818 | 3:55988394 | G | A | 0.057 | 0.0172 | 0.00337 | 5.46E-09 |
| Smoking Initiation | rs1910236 | 3:59434420 | A | G | 0.469 | 0.0083 | 0.00163 | 9.91E-09 |
| Smoking Initiation | rs7640107 | 3:59966156 | T | C | 0.430789 | -0.00679 | 0.00123 | 3.46E-08 |
| Smoking Initiation | rs2734390 | 3:60459291 | G | A | 0.372 | 0.00774 | 0.00167 | 2.09E-08 |
| Smoking Initiation | rs221988 | 3:64234307 | C | A | 0.384 | -0.00787 | 0.00172 | 1.43E-08 |
| Smoking Initiation | rs2196356 | 3:70890288 | C | G | 0.288886 | -0.00923 | 0.00138 | 2.45E-11 |
| Smoking Initiation | rs11128203 | 3:71064431 | A | T | 0.53 | 0.0109 | 0.00165 | 1.29E-15 |
| Smoking Initiation | rs62246017 | 3:71483084 | A | G | 0.322639 | -0.00791 | 0.00133 | 3.03E-09 |
| Smoking Initiation | rs4543050 | 3:74954560 | T | A | 0.816 | 0.0138 | 0.0021 | 1.45E-11 |
| Smoking Initiation | rs6782116 | 3:77176032 | T | C | 0.415 | -0.00713 | 0.00167 | 1.46E-08 |
| Smoking Initiation | rs13066050 | 3:81325861 | T | C | 0.208 | 0.00947 | 0.00195 | 1.93E-09 |
| Smoking Initiation | rs12633090 | 3:83241365 | C | G | 0.182 | -0.0111 | 0.0016 | 3.16E-12 |
| Smoking Initiation | rs1549979 | 3:85460131 | T | C | 0.615 | -0.0133 | 0.00165 | 8.80E-21 |
| Smoking Initiation | rs57153235 | 3:85902536 | G | T | 0.318 | -0.00949 | 0.001342 | 1.56E-12 |
| Smoking Initiation | rs3934797 | 4:112467612 | A | G | 0.182 | -0.0128 | 0.00212 | 1.12E-10 |
| Smoking Initiation | rs71602617 | 4:136406155 | T | C | 0.216 | -0.0098 | 0.00195 | 2.10E-08 |
| Smoking Initiation | rs7696257 | 4:137474783 | A | G | 0.366 | 0.00737 | 0.00168 | 6.78E-09 |
| Smoking Initiation | rs13109980 | 4:140886963 | A | G | 0.326 | -0.012 | 0.0017 | 3.37E-16 |
| Smoking Initiation | rs1116690 | 4:143510148 | G | A | 0.742 | 0.00963 | 0.00186 | 2.16E-08 |
| Smoking Initiation | rs13110073 | 4:147797913 | C | T | 0.395 | -0.0132 | 0.00164 | 3.24E-21 |
| Smoking Initiation | rs28717373 | 4:147985231 | T | C | 0.356165 | -0.00827 | 0.001337 | 6.16E-10 |
| Smoking Initiation | rs4140932 | 4:15458598 | A | T | 0.431 | -0.00727 | 0.00162 | 4.89E-08 |
| Smoking Initiation | rs62340589 | 4:176875795 | C | G | 0.201 | 0.0102 | 0.00198 | 4.31E-08 |
| Smoking Initiation | rs12642744 | 4:28027176 | T | G | 0.744 | -0.00807 | 0.001454 | 2.82E-08 |
| Smoking Initiation | rs59537158 | 4:28246049 | T | C | 0.214 | 0.0127 | 0.00202 | 4.62E-13 |
| Smoking Initiation | rs1389171 | 4:28822284 | A | T | 0.241 | -0.00843 | 0.001437 | 4.45E-09 |
| Smoking Initiation | rs55944129 | 4:29082156 | C | T | 0.267 | -0.00841 | 0.00138 | 1.06E-09 |
| Smoking Initiation | rs58400863 | 4:31184484 | A | G | 0.347 | -0.0102 | 0.00171 | 4.89E-14 |
| Smoking Initiation | rs7657022 | 4:35501032 | G | A | 0.489 | 0.00914 | 0.0016 | 7.34E-13 |
| Smoking Initiation | rs112725451 | 4:68017710 | T | C | 0.169 | 0.014 | 0.00215 | 1.65E-14 |
| Smoking Initiation | rs1160685 | 4:94052854 | G | C | 0.45 | 0.00992 | 0.00162 | 2.31E-09 |
| Smoking Initiation | rs1435479 | 4:94550450 | T | G | 0.28748 | 0.00784 | 0.001346 | 5.68E-09 |
| Smoking Initiation | rs72780746 | 5:103929588 | C | T | 0.173 | -0.014 | 0.00211 | 2.05E-14 |
| Smoking Initiation | rs10060196 | 5:106455988 | A | C | 0.580606 | 0.00879 | 0.00124 | 1.29E-12 |
| Smoking Initiation | rs72789626 | 5:106825618 | A | T | 0.136 | -0.0157 | 0.00229 | 5.13E-12 |
| Smoking Initiation | rs17165769 | 5:107365642 | G | A | 0.394872 | 0.00762 | 0.001246 | 9.56E-10 |
| Smoking Initiation | rs329124 | 5:133865452 | G | A | 0.428 | -0.00865 | 0.00162 | 1.96E-10 |
| Smoking Initiation | rs1385108 | 5:154839646 | T | C | 0.239 | 0.0123 | 0.00188 | 3.84E-10 |
| Smoking Initiation | rs1173461 | 5:157707571 | T | C | 0.327 | 0.0083 | 0.00172 | 9.51E-10 |
| Smoking Initiation | rs11956866 | 5:161018271 | G | T | 0.567 | -0.00813 | 0.00161 | 7.82E-09 |
| Smoking Initiation | rs3909281 | 5:165096435 | G | T | 0.536 | 0.0101 | 0.001228 | 1.62E-16 |
| Smoking Initiation | rs3843905 | 5:165427280 | T | C | 0.403 | -0.00726 | 0.001245 | 5.41E-09 |
| Smoking Initiation | rs79476395 | 5:166063680 | G | A | 0.0726 | 0.0162 | 0.00238 | 1.04E-11 |
| Smoking Initiation | rs6890961 | 5:166778503 | T | C | 0.624 | -0.00929 | 0.001266 | 2.13E-13 |
| Smoking Initiation | rs4044321 | 5:166989513 | G | A | 0.644 | -0.0139 | 0.00168 | 1.75E-17 |
| Smoking Initiation | rs2173019 | 5:167614971 | A | T | 0.177 | 0.0135 | 0.001601 | 2.98E-17 |
| Smoking Initiation | rs10042827 | 5:170299916 | C | T | 0.681 | 0.00947 | 0.00173 | 9.41E-10 |
| Smoking Initiation | rs359431 | 5:173288534 | T | C | 0.56 | -0.00762 | 0.0016 | 3.16E-08 |
| Smoking Initiation | rs12517438 | 5:30842054 | G | T | 0.538 | 0.0109 | 0.00161 | 1.89E-09 |
| Smoking Initiation | rs35375873 | 5:43190647 | C | G | 0.11 | -0.0162 | 0.00268 | 3.29E-11 |
| Smoking Initiation | rs986714 | 5:50821338 | T | A | 0.445 | -0.0077 | 0.00161 | 4.13E-10 |
| Smoking Initiation | rs71592686 | 5:60121271 | C | T | 0.274 | 0.0121 | 0.00179 | 3.85E-13 |
| Smoking Initiation | rs2028269 | 5:79308315 | A | G | 0.399 | 0.00773 | 0.00124 | 5.19E-10 |
| Smoking Initiation | rs6874731 | 5:80263865 | G | T | 0.484 | 0.00899 | 0.00161 | 1.83E-09 |
| Smoking Initiation | rs6452785 | 5:87685500 | T | C | 0.474 | -0.0152 | 0.00161 | 4.69E-26 |
| Smoking Initiation | rs10805858 | 5:88873832 | T | A | 0.335286 | 0.00874 | 0.001301 | 1.88E-11 |
| Smoking Initiation | rs42417 | 5:94198290 | T | C | 0.691 | 0.0116 | 0.00174 | 8.27E-10 |
| Smoking Initiation | rs6936160 | 6:100347745 | T | C | 0.698 | 0.00963 | 0.00133 | 4.20E-13 |
| Smoking Initiation | rs12530388 | 6:101329173 | C | A | 0.511 | -0.0104 | 0.0016 | 5.83E-13 |
| Smoking Initiation | rs3800227 | 6:108994161 | G | A | 0.742 | 0.0104 | 0.00184 | 3.64E-09 |
| Smoking Initiation | rs118202 | 6:111658371 | T | G | 0.812 | -0.0196 | 0.00204 | 1.90E-29 |
| Smoking Initiation | rs73008357 | 6:156431856 | C | A | 0.121 | -0.0121 | 0.00244 | 2.44E-08 |
| Smoking Initiation | rs9331343 | 6:157738258 | C | T | 0.568 | -0.00875 | 0.00173 | 3.90E-08 |
| Smoking Initiation | rs10698713 | 6:158882320 | A | G | 0.0544 | -0.0184 | 0.00368 | 2.38E-09 |
| Smoking Initiation | rs1737329 | 6:163807748 | G | C | 0.742 | 0.00935 | 0.00182 | 5.08E-09 |
| Smoking Initiation | rs1059490 | 6:26171250 | C | T | 0.367 | -0.0102 | 0.0017 | 2.16E-12 |
| Smoking Initiation | rs6932350 | 6:26571629 | A | T | 0.454655 | 0.00725 | 0.00124 | 5.13E-09 |
| Smoking Initiation | rs1150668 | 6:28129789 | G | T | 0.419 | -0.01 | 0.00173 | 8.54E-13 |
| Smoking Initiation | rs1632941 | 6:29796685 | C | T | 0.46 | -0.00767 | 0.001242 | 6.67E-10 |
| Smoking Initiation | rs3218116 | 6:41901763 | T | C | 0.256 | -0.0105 | 0.00182 | 1.05E-11 |
| Smoking Initiation | rs160631 | 6:52895230 | G | T | 0.731 | -0.00981 | 0.0018 | 1.87E-09 |
| Smoking Initiation | rs7743165 | 6:67521222 | G | T | 0.495 | 0.0102 | 0.0016 | 4.15E-14 |
| Smoking Initiation | rs10945141 | 6:69470709 | A | G | 0.263 | 0.00936 | 0.00181 | 3.59E-10 |
| Smoking Initiation | rs17554906 | 6:92226609 | C | G | 0.444 | 0.00806 | 0.00161 | 3.14E-08 |
| Smoking Initiation | rs619087 | 6:94175279 | G | A | 0.422 | 0.00809 | 0.00162 | 3.10E-08 |
| Smoking Initiation | rs6568832 | 6:97702876 | A | G | 0.753851 | 0.00903 | 0.001415 | 1.74E-10 |
| Smoking Initiation | rs12195240 | 6:98636905 | A | G | 0.285 | 0.013 | 0.00178 | 1.08E-18 |
| Smoking Initiation | rs11766326 | 7:111100585 | C | T | 0.506 | -0.00821 | 0.00161 | 1.79E-11 |
| Smoking Initiation | rs6968380 | 7:114940159 | A | G | 0.681 | -0.013 | 0.00174 | 1.05E-17 |
| Smoking Initiation | rs10233018 | 7:117523709 | G | A | 0.516 | 0.0149 | 0.00161 | 4.77E-22 |
| Smoking Initiation | rs10953957 | 7:121954709 | A | G | 0.386 | 0.00907 | 0.00167 | 3.66E-08 |
| Smoking Initiation | rs77283305 | 7:132593831 | A | G | 0.305819 | -0.00728 | 0.00133 | 3.91E-08 |
| Smoking Initiation | rs10279261 | 7:133589846 | A | G | 0.618 | -0.0114 | 0.00169 | 6.05E-13 |
| Smoking Initiation | rs1561112 | 7:133840652 | C | T | 0.412815 | -0.00742 | 0.00126 | 3.84E-09 |
| Smoking Initiation | rs10272990 | 7:1703675 | C | T | 0.327622 | -0.0102 | 0.00132 | 1.27E-14 |
| Smoking Initiation | rs6948707 | 7:1870794 | G | T | 0.419 | 0.0129 | 0.00163 | 4.24E-21 |
| Smoking Initiation | rs10259715 | 7:3329967 | A | T | 0.209918 | -0.00974 | 0.00168 | 6.42E-09 |
| Smoking Initiation | rs13237637 | 7:3503207 | C | G | 0.485 | -0.0123 | 0.0016 | 1.54E-20 |
| Smoking Initiation | rs7809303 | 7:69484366 | A | G | 0.325 | -0.0115 | 0.0017 | 3.48E-15 |
| Smoking Initiation | rs7802996 | 7:77771983 | T | C | 0.166 | -0.0123 | 0.00211 | 1.06E-09 |
| Smoking Initiation | rs1030015 | 7:78139581 | T | G | 0.519564 | 0.00682 | 0.001218 | 2.15E-08 |
| Smoking Initiation | rs4727189 | 7:88442568 | C | T | 0.344 | 0.00772 | 0.00168 | 3.00E-08 |
| Smoking Initiation | rs76841737 | 7:91281409 | G | C | 0.103 | -0.0118 | 0.00261 | 3.26E-08 |
| Smoking Initiation | rs11768481 | 7:96629103 | A | C | 0.34 | -0.0111 | 0.00176 | 5.23E-12 |
| Smoking Initiation | rs1799068 | 7:97707069 | T | G | 0.379 | 0.00838 | 0.00164 | 2.59E-10 |
| Smoking Initiation | rs13437771 | 7:99071478 | G | A | 0.155 | -0.015 | 0.00221 | 1.39E-14 |
| Smoking Initiation | rs2952251 | 8:10143164 | G | A | 0.74437 | 0.00816 | 0.00149 | 4.24E-08 |
| Smoking Initiation | rs4326350 | 8:10763655 | G | C | 0.493 | -0.00894 | 0.00162 | 5.16E-12 |
| Smoking Initiation | rs290601 | 8:115374642 | T | C | 0.274 | 0.0085 | 0.00178 | 1.14E-08 |
| Smoking Initiation | rs11783093 | 8:27425349 | T | C | 0.158 | -0.0253 | 0.00225 | 2.07E-41 |
| Smoking Initiation | rs7836565 | 8:52569449 | T | C | 0.718 | -0.00923 | 0.00178 | 4.36E-08 |
| Smoking Initiation | rs13261666 | 8:59814666 | T | G | 0.517 | -0.0117 | 0.0016 | 4.36E-15 |
| Smoking Initiation | rs3850736 | 8:64912021 | G | C | 0.474 | 0.0109 | 0.00161 | 6.43E-14 |
| Smoking Initiation | rs2063976 | 8:91096366 | T | C | 0.664955 | -0.00968 | 0.001294 | 7.45E-14 |
| Smoking Initiation | rs6993429 | 8:92733282 | A | C | 0.453 | -0.00984 | 0.00161 | 9.87E-14 |
| Smoking Initiation | rs6986430 | 8:93048104 | C | T | 0.222377 | -0.0117 | 0.00147 | 1.99E-15 |
| Smoking Initiation | rs9987376 | 8:93190014 | G | T | 0.574251 | -0.00982 | 0.001237 | 2.01E-15 |
| Smoking Initiation | rs6474609 | 9:10981069 | A | T | 0.586731 | -0.00757 | 0.001257 | 1.71E-09 |
| Smoking Initiation | rs1931431 | 9:11161799 | C | G | 0.478 | 0.00884 | 0.0016 | 8.56E-13 |
| Smoking Initiation | rs1927901 | 9:120519111 | C | T | 0.553 | -0.00859 | 0.00161 | 3.10E-08 |
| Smoking Initiation | rs4837631 | 9:122061948 | T | C | 0.446 | -0.00878 | 0.0016 | 2.03E-09 |
| Smoking Initiation | rs1759433 | 9:128073097 | A | G | 0.48 | 0.0076 | 0.0016 | 1.69E-09 |
| Smoking Initiation | rs34553878 | 9:134334588 | G | A | 0.111 | 0.027377 | 0.067088 | 1.17E-09 |
| Smoking Initiation | rs7026534 | 9:134907263 | G | T | 0.703821 | -0.00793 | 0.00133 | 2.68E-09 |
| Smoking Initiation | rs10858334 | 9:137989785 | G | C | 0.14 | 0.013 | 0.0024 | 1.18E-09 |
| Smoking Initiation | rs7867822 | 9:20676454 | G | A | 0.673 | -0.00839 | 0.0017 | 2.76E-08 |
| Smoking Initiation | rs10966092 | 9:23831658 | C | T | 0.267 | -0.0104 | 0.00181 | 1.12E-12 |
| Smoking Initiation | rs10969352 | 9:29747488 | A | T | 0.5 | 0.00722 | 0.0016 | 1.82E-08 |
| Smoking Initiation | rs3847244 | 9:3025368 | T | C | 0.47 | 0.01 | 0.00165 | 2.60E-13 |
| Smoking Initiation | rs11791671 | 9:3398679 | T | C | 0.067315 | 0.0134 | 0.00244 | 4.24E-08 |
| Smoking Initiation | rs4877285 | 9:81354129 | A | G | 0.668249 | -0.00881 | 0.00131 | 2.10E-11 |
| Smoking Initiation | rs1930371 | 9:81444104 | T | C | 0.241 | -0.0094 | 0.00189 | 7.09E-09 |
| Smoking Initiation | rs7024924 | 9:8282399 | C | T | 0.174 | 0.0108 | 0.00213 | 1.90E-08 |
| Smoking Initiation | rs2378662 | 9:86707289 | A | G | 0.541 | 0.00879 | 0.00162 | 2.67E-09 |
| Waist circumference | rs7550711 | 1:110082886 | T | C | 0.0339 | 0.058 | 0.0098 | 3.40E-09 |
| Waist circumference | rs633715 | 1:177852580 | C | T | 0.2667 | 0.043 | 0.0043 | 3.30E-23 |
| Waist circumference | rs2820292 | 1:201784287 | A | C | 0.4917 | -0.019 | 0.0034 | 2.40E-08 |
| Waist circumference | rs3127553 | 1:49438005 | G | A | 0.3667 | 0.023 | 0.0035 | 1.60E-10 |
| Waist circumference | rs7531118 | 1:72837239 | T | C | 0.3917 | -0.027 | 0.0035 | 1.50E-14 |
| Waist circumference | rs4130548 | 1:78463868 | C | T | 0.425 | 0.022 | 0.0035 | 3.40E-10 |
| Waist circumference | rs11165623 | 1:96893000 | A | G | 0.4833 | 0.02 | 0.0034 | 5.20E-09 |
| Waist circumference | rs6163 | 10:104596924 | C | A | 0.6083 | -0.019 | 0.0035 | 3.70E-08 |
| Waist circumference | rs7903146 | 10:114758349 | T | C | 0.25 | -0.022 | 0.0037 | 3.90E-09 |
| Waist circumference | rs10767658 | 11:27672252 | C | G | 0.3583 | 0.031 | 0.0037 | 3.30E-17 |
| Waist circumference | rs2293576 | 11:47434986 | A | G | 0.3667 | -0.022 | 0.0036 | 9.40E-10 |
| Waist circumference | rs10840100 | 11:8669437 | G | A | 0.725 | 0.02 | 0.0035 | 5.40E-09 |
| Waist circumference | rs7138803 | 12:50247468 | G | A | 0.5583 | -0.028 | 0.0035 | 1.60E-15 |
| Waist circumference | rs12429545 | 13:54102206 | G | A | 0.9 | -0.031 | 0.0052 | 2.50E-09 |
| Waist circumference | rs10132280 | 14:25928179 | A | C | 0.3333 | -0.022 | 0.0037 | 2.20E-09 |
| Waist circumference | rs12885454 | 14:29736838 | C | A | 0.6333 | 0.02 | 0.0035 | 2.60E-08 |
| Waist circumference | rs7144011 | 14:79940383 | T | G | 0.275 | 0.033 | 0.0041 | 9.40E-16 |
| Waist circumference | rs4776970 | 15:68080886 | T | A | 0.3417 | -0.02 | 0.0035 | 2.30E-08 |
| Waist circumference | rs12446632 | 16:19935389 | A | G | 0.1333 | -0.036 | 0.005 | 5.20E-13 |
| Waist circumference | rs7498665 | 16:28883241 | G | A | 0.3583 | 0.034 | 0.0035 | 1.40E-22 |
| Waist circumference | rs1549293 | 16:31141993 | T | C | 0.3917 | -0.02 | 0.0035 | 7.30E-09 |
| Waist circumference | rs2531992 | 16:4021734 | A | G | 0.1667 | -0.028 | 0.0048 | 3.00E-09 |
| Waist circumference | rs1558902 | 16:53803574 | A | T | 0.45 | 0.074 | 0.0035 | ###### |
| Waist circumference | rs7239883 | 18:40147671 | G | A | 0.3167 | 0.021 | 0.0035 | 2.30E-09 |
| Waist circumference | rs6567160 | 18:57829135 | C | T | 0.2833 | 0.048 | 0.004 | 2.60E-33 |
| Waist circumference | rs11873305 | 18:58049192 | A | C | 0.9667 | 0.058 | 0.0092 | 3.10E-10 |
| Waist circumference | rs2075650 | 19:45395619 | A | G | 0.8583 | 0.031 | 0.005 | 8.90E-10 |
| Waist circumference | rs2287019 | 19:46202172 | C | T | 0.85 | 0.035 | 0.0046 | 1.70E-14 |
| Waist circumference | rs3810291 | 19:47569003 | A | G | 0.625 | 0.026 | 0.004 | 1.70E-10 |
| Waist circumference | rs929641 | 2:58792377 | A | G | 0.6167 | 0.021 | 0.0034 | 1.20E-09 |
| Waist circumference | rs6545714 | 2:59307725 | G | A | 0.375 | 0.022 | 0.0035 | 1.90E-10 |
| Waist circumference | rs6755502 | 2:635721 | T | C | 0.125 | -0.051 | 0.0045 | 2.00E-30 |
| Waist circumference | rs16996700 | 20:50981945 | T | C | 0.7 | 0.023 | 0.0037 | 1.50E-09 |
| Waist circumference | rs6440003 | 3:141094209 | G | A | 0.5167 | -0.021 | 0.0034 | 2.90E-10 |
| Waist circumference | rs1516725 | 3:185824004 | T | C | 0.0917 | -0.031 | 0.0051 | 1.70E-09 |
| Waist circumference | rs3849570 | 3:81792112 | A | C | 0.3667 | 0.021 | 0.0038 | 2.20E-08 |
| Waist circumference | rs2325036 | 3:85819412 | A | C | 0.5917 | 0.023 | 0.0035 | 2.10E-11 |
| Waist circumference | rs10938397 | 4:45182527 | A | G | 0.5667 | -0.032 | 0.0035 | 6.10E-20 |
| Waist circumference | rs2112347 | 5:75015242 | G | T | 0.375 | -0.025 | 0.0035 | 3.20E-13 |
| Waist circumference | rs9400239 | 6:108977663 | C | T | 0.7 | 0.024 | 0.0036 | 1.90E-11 |
| Waist circumference | rs2489623 | 6:127455821 | C | A | 0.5583 | 0.019 | 0.0034 | 3.40E-08 |
| Waist circumference | rs806794 | 6:26200677 | G | A | 0.275 | -0.022 | 0.0037 | 2.10E-09 |
| Waist circumference | rs16894959 | 6:34825662 | C | T | 0.1 | 0.026 | 0.0048 | 3.40E-08 |
| Waist circumference | rs943005 | 6:50865820 | T | C | 0.1 | 0.039 | 0.0044 | 7.20E-19 |
| Waist circumference | rs10968576 | 9:28414339 | G | A | 0.2917 | 0.025 | 0.0036 | 1.20E-11 |
| WAB | rs7536458 | 1:118864602 | G | T | 0.35 | -0.03 | 0.0038 | 1.20E-15 |
| WAB | rs984222 | 1:119503843 | C | G | 0.425 | -0.036 | 0.0035 | 1.50E-25 |
| WAB | rs11205277 | 1:149892872 | G | A | 0.3898 | 0.027 | 0.0036 | 1.30E-13 |
| WAB | rs9435732 | 1:17308158 | T | C | 0.175 | -0.031 | 0.0038 | 4.10E-16 |
| WAB | rs2274432 | 1:184020945 | A | G | 0.3729 | 0.025 | 0.0036 | 1.70E-12 |
| WAB | rs991967 | 1:218615451 | C | A | 0.1897 | 0.026 | 0.0037 | 1.10E-12 |
| WAB | rs12127195 | 1:221309417 | A | G | 0.325 | 0.021 | 0.0037 | 7.70E-09 |
| WAB | rs10748826 | 10:104354804 | C | T | 0.4224 | -0.023 | 0.0037 | 3.40E-10 |
| WAB | rs780159 | 10:80907147 | G | A | 0.525 | 0.021 | 0.0035 | 1.80E-09 |
| WAB | rs606452 | 11:75276178 | A | C | 0.1583 | 0.028 | 0.0048 | 1.10E-08 |
| WAB | rs12317176 | 12:124404718 | T | C | 0.6167 | 0.02 | 0.0035 | 5.90E-09 |
| WAB | rs12372180 | 12:124746863 | A | G | 0.0667 | 0.041 | 0.0073 | 2.70E-08 |
| WAB | rs2638953 | 12:28534415 | C | G | 0.6333 | 0.024 | 0.0036 | 6.50E-11 |
| WAB | rs2071449 | 12:54428011 | A | C | 0.325 | 0.032 | 0.0036 | 2.50E-18 |
| WAB | rs7970350 | 12:66360164 | C | T | 0.5083 | 0.019 | 0.0034 | 3.80E-08 |
| WAB | rs2160077 | 14:92428410 | G | A | 0.6083 | 0.018 | 0.0033 | 4.50E-08 |
| WAB | rs4246302 | 15:100687967 | G | A | 0.3333 | 0.022 | 0.0037 | 5.70E-09 |
| WAB | rs4567683 | 15:100778837 | A | G | 0.2833 | 0.022 | 0.0038 | 7.70E-09 |
| WAB | rs7166081 | 15:67492301 | A | G | 0.8083 | 0.024 | 0.0039 | 2.10E-09 |
| WAB | rs4886782 | 15:74228810 | G | A | 0.7333 | 0.024 | 0.0036 | 6.00E-12 |
| WAB | rs7162542 | 15:84514290 | G | C | 0.5167 | 0.038 | 0.0034 | 9.70E-29 |
| WAB | rs1879529 | 15:89414295 | G | T | 0.7667 | 0.024 | 0.0038 | 2.90E-10 |
| WAB | rs2047937 | 16:49864791 | C | T | 0.4667 | 0.019 | 0.0034 | 4.70E-08 |
| WAB | rs16957304 | 16:67334969 | A | G | 0.95 | 0.059 | 0.011 | 2.50E-08 |
| WAB | rs3760318 | 17:29247715 | G | A | 0.6417 | 0.021 | 0.0035 | 9.00E-10 |
| WAB | rs757608 | 17:59497277 | A | G | 0.3 | 0.027 | 0.0036 | 1.00E-13 |
| WAB | rs4239436 | 18:20731930 | G | A | 0.7417 | 0.04 | 0.0041 | 1.00E-22 |
| WAB | rs12608504 | 19:18389135 | A | G | 0.3417 | 0.02 | 0.0036 | 1.50E-08 |
| WAB | rs3786897 | 19:33893008 | G | A | 0.4083 | 0.02 | 0.0035 | 8.80E-09 |
| WAB | rs4542783 | 19:8642160 | C | T | 0.3879 | -0.023 | 0.004 | 1.70E-08 |
| WAB | rs2124969 | 2:160989486 | C | T | 0.4083 | 0.02 | 0.0034 | 7.10E-09 |
| WAB | rs12991495 | 2:25486770 | T | C | 0.675 | 0.028 | 0.0037 | 6.20E-14 |
| WAB | rs6715793 | 2:33379263 | T | C | 0.45 | 0.019 | 0.0034 | 1.40E-08 |
| WAB | rs3791679 | 2:56096892 | A | G | 0.725 | 0.035 | 0.0039 | 2.10E-19 |
| WAB | rs2052670 | 2:66218481 | G | A | 0.4083 | 0.02 | 0.0035 | 1.50E-08 |
| WAB | rs979012 | 20:6623374 | T | C | 0.3583 | 0.033 | 0.0036 | 5.40E-20 |
| WAB | rs9977276 | 21:47436327 | G | T | 0.75 | 0.022 | 0.004 | 4.40E-08 |
| WAB | rs2179129 | 22:29450923 | A | G | 0.55 | 0.019 | 0.0034 | 2.60E-08 |
| WAB | rs6772896 | 3:134203347 | T | C | 0.6417 | 0.024 | 0.0036 | 1.80E-11 |
| WAB | rs7621331 | 3:135761927 | A | G | 0.6917 | 0.021 | 0.0036 | 9.40E-09 |
| WAB | rs1344674 | 3:141125186 | A | G | 0.5167 | -0.024 | 0.0033 | 4.30E-13 |
| WAB | rs17451107 | 3:156797609 | T | C | 0.625 | 0.026 | 0.0036 | 1.30E-13 |
| WAB | rs12493901 | 3:171922055 | G | A | 0.5417 | 0.021 | 0.0034 | 8.30E-10 |
| WAB | rs13083798 | 3:52649748 | A | G | 0.5417 | 0.02 | 0.0034 | 3.40E-09 |
| WAB | rs9864077 | 3:64704891 | T | C | 0.7583 | 0.022 | 0.0037 | 1.30E-09 |
| WAB | rs12330322 | 3:72455355 | C | T | 0.775 | 0.022 | 0.004 | 3.20E-08 |
| WAB | rs1812175 | 4:145574844 | A | G | 0.1917 | -0.033 | 0.0045 | 4.00E-13 |
| WAB | rs7684221 | 4:17957354 | A | G | 0.1917 | -0.026 | 0.0047 | 4.20E-08 |
| WAB | rs710841 | 4:82149831 | T | C | 0.2417 | 0.029 | 0.0038 | 8.50E-14 |
| WAB | rs10041657 | 5:108152428 | A | G | 0.2167 | 0.025 | 0.004 | 2.90E-10 |
| WAB | rs272869 | 5:131677997 | G | A | 0.6583 | 0.021 | 0.0034 | 6.70E-10 |
| WAB | rs4868125 | 5:171281875 | G | C | 0.6417 | 0.021 | 0.0036 | 2.90E-09 |
| WAB | rs10516107 | 5:173348156 | A | G | 0.2917 | 0.023 | 0.0036 | 8.30E-11 |
| WAB | rs6556301 | 5:176527577 | T | G | 0.375 | 0.028 | 0.0039 | 1.80E-12 |
| WAB | rs12656497 | 5:32831939 | T | C | 0.4833 | 0.022 | 0.0034 | 2.00E-10 |
| WAB | rs459193 | 5:55806751 | A | G | 0.2167 | 0.025 | 0.0038 | 7.70E-11 |
| WAB | rs395962 | 6:105397418 | T | G | 0.3667 | 0.029 | 0.0036 | 1.30E-15 |
| WAB | rs2745353 | 6:127452935 | T | C | 0.55 | 0.029 | 0.0033 | 7.90E-19 |
| WAB | rs9389986 | 6:142661114 | A | T | 0.25 | -0.024 | 0.0037 | 5.70E-11 |
| WAB | rs806794 | 6:26200677 | G | A | 0.275 | -0.03 | 0.0037 | 1.90E-15 |
| WAB | rs1776897 | 6:34195011 | G | T | 0.075 | 0.061 | 0.0067 | 5.60E-20 |
| WAB | rs13210323 | 6:35005084 | A | C | 0.725 | 0.022 | 0.0038 | 1.40E-08 |
| WAB | rs998584 | 6:43757896 | A | C | 0.475 | 0.029 | 0.0038 | 6.40E-15 |
| WAB | rs12207675 | 6:76237741 | C | T | 0.1333 | 0.031 | 0.0052 | 3.10E-09 |
| WAB | rs822531 | 7:148629759 | T | C | 0.7333 | 0.024 | 0.0044 | 3.70E-08 |
| WAB | rs2214442 | 7:20392787 | G | A | 0.4417 | 0.026 | 0.0045 | 3.90E-09 |
| WAB | rs4141278 | 7:25857525 | C | T | 0.1833 | 0.034 | 0.0043 | 3.40E-15 |
| WAB | rs798489 | 7:2801803 | C | T | 0.725 | 0.025 | 0.0037 | 1.30E-11 |
| WAB | rs849140 | 7:28183702 | T | C | 0.4 | 0.029 | 0.0034 | 4.70E-17 |
| WAB | rs12679556 | 8:72514228 | G | T | 0.2083 | 0.026 | 0.0039 | 1.30E-11 |
| WAB | rs11144688 | 9:78542286 | G | A | 0.9083 | 0.034 | 0.006 | 1.90E-08 |
| WAB | rs473902 | 9:98256235 | T | G | 0.9417 | 0.049 | 0.0071 | 4.30E-12 |

Chr, chromosome; EA, effect allele; EAF, effect allele frequency; NEA, non-effect allele; WAB, waist circumference adjusted for body mass index.

Supplementary Table 3. Variance explained, average F-statistic, and power calculation

| Exposure or outcome | Variance explained | Average F-statistic | OR at 80% power in UK Biobank | OR at 80% power in FinnGen |
| --- | --- | --- | --- | --- |
| Obesity |  |  |  |  |
| Waist circumference | 1.52% | 75.8 | ≤0.78 or ≥1.22 | ≤0.70 or ≥1.34 |
| Waist circumference adjusted for BMI | 2.01% | 62.5 | ≤0.81 or ≥1.20 | ≤0.77 or ≥1.32 |
| Body mass index | 5.62% | 83.5 | ≤0.89 or ≥1.12 | ≤0.89 or ≥1.11 |
| Lifestyle factors |  |  |  |  |
| Relative carbohydrate intake | 0.20% | 39.6 | ≤0.56 or ≥1.56 | ≤0.52 or ≥1.62 |
| Relative fat intake | 0.17% | 76.7 | ≤0.55 or ≥1.57 | ≤0.51 or ≥1.63 |
| Relative protein intake | 0.15% | 45.8 | ≤0.53 or ≥1.59 | ≤0.50 or ≥1.64 |
| Relative sugar intake | 0.21% | 34.7 | ≤0.58 or ≥1.54 | ≤0.55 or ≥1.60 |
| Smoking initiation | 1.57% | 53.1 | ≤0.78 or ≥1.22 | ≤0.80 or ≥1.22 |
| Smoking per day | 1.18% | 122 | ≤0.75 or ≥1.26 | ≤0.76 or ≥1.25 |
| Lifetime smoking index | 0.54% | 19.7 | ≤0.63 or ≥1.38 | ≤0.66 or ≥1.38 |
| Alcohol per week | 0.65% | 156 | ≤0.67 or ≥1.35 | ≤0.69 or ≥1.35 |
| Sedentary behavior | 0.70% | 22.2 | ≤0.67 or ≥1.34 | ≤0.70 or ≥1.33 |

R^2^ was calculated as follows: R^2^ =2×EAF*(1−EAF)*Beta^2^;

The F-statistic for each SNP was calculated as follows: F=(N−2)*R^2^/(1−R^2^);

N represents the sample size; EAF represents effect allele frequency.

Supplementary Table 4. The results of pleiotropy test, Cochrane’s Q and MR-PRESSO

| Exposure | *P* for pleiotropy^a^ | Cochrane’s Q | *P* for Cochrane’s Q | MR-PRESSO (Raw) | | | MR-PRESSO (Outlier-corrected) | | | *P* for global test | *P* for distortion test^b^ |
| --- | --- | --- | --- | --- | --- | --- | --- | --- | --- | --- | --- |
|  |  |  |  | **OR** | **95% Cl** | ***P*** | **OR** | **95% Cl** | ***P*** |  |  |
| UK Biobank |  |  |  |  |  |  |  |  |  |  |  |
| Obesity |  |  |  |  |  |  |  |  |  |  |  |
| Waist circumference | 0.163 | 87.1 | <0.001 | 1.10 | 1.01-1.52 | 0.0418 | 1.19 | 1.00-1.41 | 0.0494 | <0.001 | 0.469 |
| Waist circumference adjusted for BMI | 0.947 | 564.0 | <0.001 | 1.18 | 1.11-1.55 | 0.0018 | 1.26 | 1.08-1.47 | 0.00293 | <0.001 | 0.591 |
| Body mass index | 0.392 | 715 | <0.001 | 1.14 | 1.05-1.26 | 0.0018 | 1.14 | 1.05-1.25 | 0.00173 | <0.001 | 0.872 |
| Lifestyle factors |  |  |  |  |  |  |  |  |  |  |  |
| Relative carbohydrate intake | 0.974 | 10.8 | 0.547 | 0.80 | 0.55-1.17 | 0.273 | NA | NA | NA | 0.572 | NA |
| Relative fat intake | 0.243 | 3.5 | 0.620 | 1.09 | 0.75-1.60 | 0.659 | NA | NA | NA | 0.630 | NA |
| Relative protein intake | 0.482 | 2.5 | 0.820 | 1.05 | 0.77-1.42 | 0.774 | NA | NA | NA | 0.774 | NA |
| Relative sugar intake | 0.805 | 5.2 | 0.820 | 0.96 | 0.71-1.29 | 0.795 | NA | NA | NA | 0.824 | NA |
| Smoking initiation | 0.559 | 471.1 | <0.001 | 1.08 | 0.88-1.22 | 0.631 | 1.01 | 0.86-1.18 | 0.885 | <0.001 | 0.142 |
| Smoking per day | 0.461 | 19.4 | 0.494 | 1.08 | 0.84-1.17 | 0.997 | NA | NA | NA | 0.522 | NA |
| Lifetime smoking index | 0.252 | 175.4 | 0.002 | 1.16 | 0.98-1.76 | 0.0641 | NA | NA | NA | 0.005 | NA |
| Alcohol per week | 0.670 | 38.4 | 0.407 | 0.81 | 0.63-1.04 | 0.104 | NA | NA | NA | 0.417 | NA |
| Sedentary behavior | 0.452 | 715 | <0.001 | 1.29 | 1.01-1.65 | 0.0399 | 1.25 | 1.00-1.59 | 0.0456 | 0.017 | 0.755 |
| FinnGen consortium |  |  |  |  |  |  |  |  |  |  |  |
| Obesity |  |  |  |  |  |  |  |  |  |  |  |
| Waist circumference | 0.934 | 61.3 | 0.043 | 1.11 | 0.99-1.51 | 0.0609 | 1.19 | 0.97-1.45 | 0.0919 | 0.0410 | 0.681 |
| Waist circumference adjusted for BMI | 0.290 | 115.7 | <0.001 | 1.10 | 0.96-1.44 | 0.1079 | 1.22 | 1.01-1.48 | 0.0401 | <0.001 | 0.718 |
| Body mass index | 0.087 | 629.3 | <0.001 | 1.05 | 0.89-1.09 | 0.840 | 1.02 | 0.92-1.09 | 0.7750 | <0.001 | 0.948 |
| Lifestyle factors |  |  |  |  |  |  |  |  |  |  |  |
| Relative carbohydrate intake | 0.802 | 11.7 | 0.306 | 1.29 | 0.72-2.31 | 0.407 | NA | NA | NA | 0.318 | NA |
| Relative fat intake | 0.735 | 4.0 | 0.407 | 1.94 | 0.95-3.95 | 0.143 | NA | NA | NA | 0.425 | NA |
| Relative protein intake | 0.479 | 2.3 | 0.799 | 1.62 | 0.98-2.45 | 0.069 | NA | NA | NA | 0.0694 | NA |
| Relative sugar intake | 0.909 | 9.8 | 0.371 | 1.18 | 0.71-1.97 | 0.537 | NA | NA | NA | 0.388 | NA |
| Smoking initiation | 0.395 | 431.1 | <0.001 | 1.11 | 0.86-1.30 | 0.558 | 1.03 | 0.84-1.27 | 0.726 | <0.001 | 0.522 |
| Smoking per day | 0.667 | 31.4 | 0.037 | 1.13 | 1.06-1.75 | 0.0237 | NA | NA | NA | 0.044 | NA |
| Lifetime smoking index | 0.406 | 127 | 0.385 | 1.17 | 0.85-1.61 | 0.316 | NA | NA | NA | 0.367 | NA |
| Alcohol per week | 0.152 | 23.8 | 0.966 | 1.13 | 0.67-1.13 | 0.322 | NA | NA | NA | 0.963 | NA |
| Sedentary behavior | 0.984 | 154.7 | 0.077 | 1.16 | 0.99-1.79 | 0.0596 | NA | NA | NA | 0.093 | NA |

a P-values for pleiotropy were derived from MR-Egger test and *P*-value<0.05 indicates a possible pleiotropic effect.

b P-values for distortion were derived from MR-PRESSO test and *P*-value<0.05 indicates a difference between estimates before and after outlier removal. P of distortion test was not available for the analysis of relative carbohydrate intake, relative fat intake, relative protein intake, relative sugar intake, smoking per day and lifetime smoking index based on UK Biobank and relative carbohydrate intake, relative fat intake, relative protein intake, relative sugar intake, smoking per day, lifetime smoking index, alcohol per week and sedentary behavior on FinnGen consortium due to no outlier detected.

NA, not available; OR, Odds ratio; CI, confidence interval


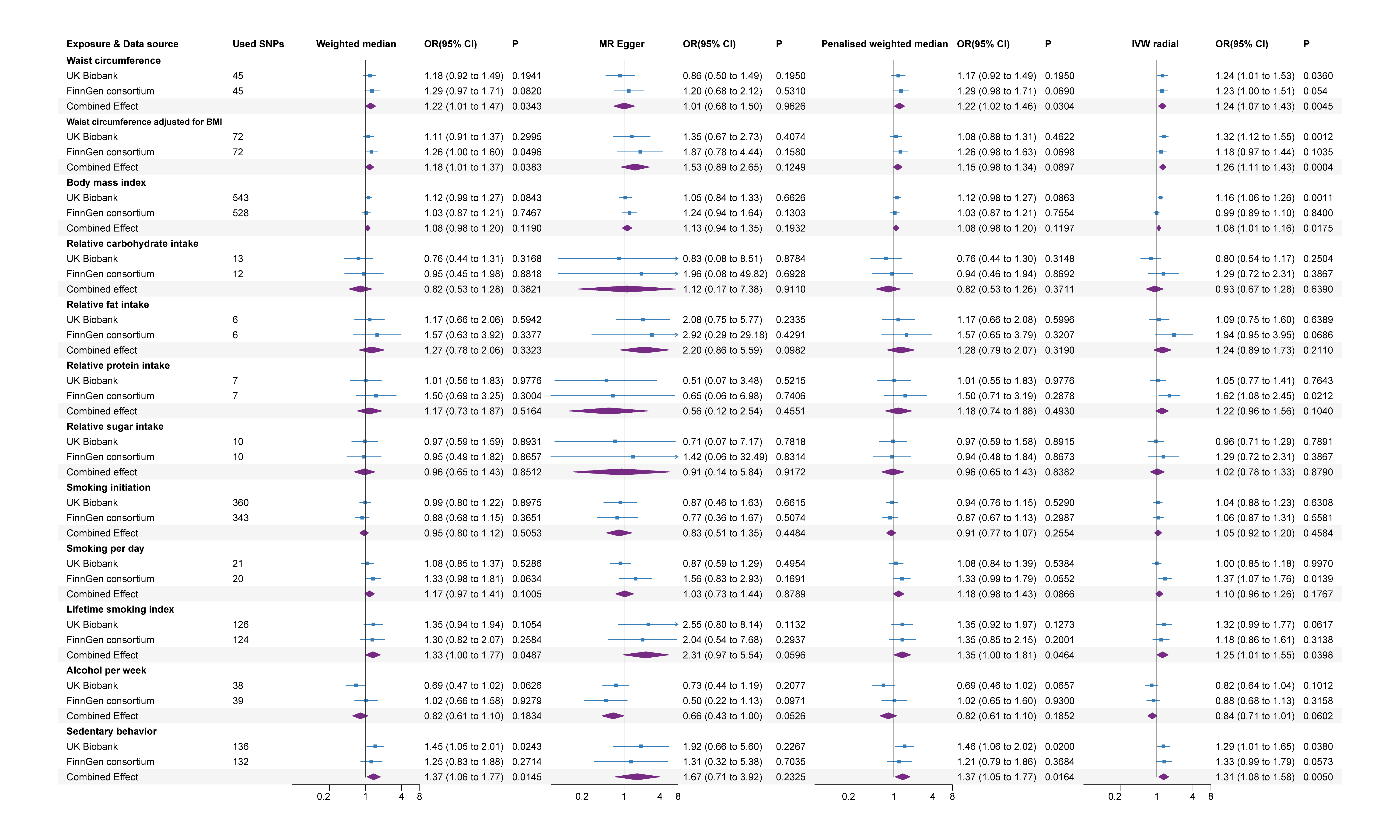


Supplementary Figure 1. Associations of genetically predicted risk factors with benign prostatic hyperplasia using multiple MR sensitivity analyses.

IVW, inverse-variance weighted; OR, odds ratio; CI, confidence interval; BMI, body mass index; SNP, single nucleotide polymorphism; MR, Mendelian randomization.
